# Photochemical Access to Substituted β-Lactams and β-Lactones via the Zimmerman–O’Connell–Griffin Rearrangement

**DOI:** 10.1021/acs.orglett.3c01990

**Published:** 2023-07-18

**Authors:** August Runemark, Mario Martos, Martin Nigríni, Francoise M. Amombo Noa, Lars Öhrström, Henrik Sundén

**Affiliations:** † Department of Chemistry and Chemical Engineering, Chalmers University of Technology, Kemivägen 10, 412 96 Gothenburg, Sweden; ‡ Organic Chemistry Department and Institute of Organic Synthesis (ISO), University of Alicante, ctra. San Vicente del Raspeig s/n, 03690 Alicante, Spain; § Department of Organic Chemistry, Faculty of Science, Charles University, Hlavova 2030, 128 43 Prague, Czech Republic; ∥ Department of Chemistry and Molecular Biology, University of Gothenburg, Kemivägen 10, 412 96 Gothenburg, Sweden

## Abstract

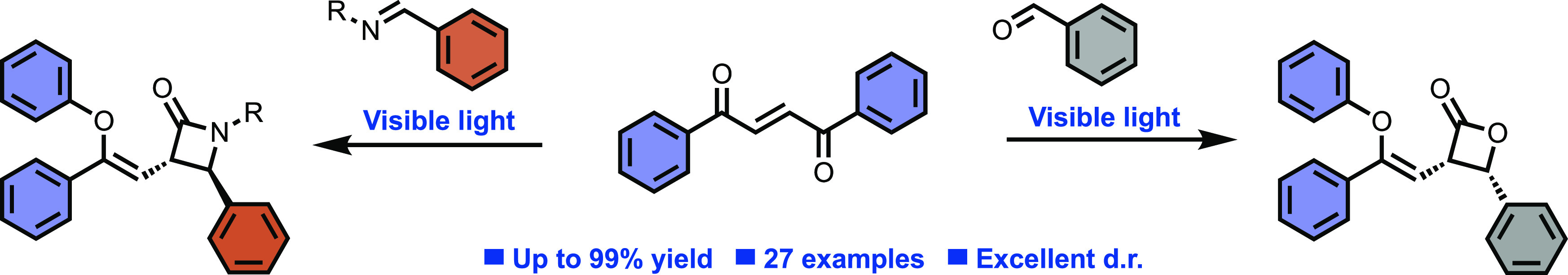

A photomediated protocol giving facile access to substituted β-lactam and β-lactones is presented. The method realizes, for the first time, the use of the Zimmerman–O’Connell–Griffin (ZOG) rearrangement in [2 + 2] cycloaddition. Products are obtained in high yield with excellent diastereoselectivity under visible light irradiation and under mild reaction conditions.

β-Lactam and β-lactone rings stand out as key structural scaffolds found in many drugs and leading bioactive candidates (Scheme 1B). The most famous members of this family are different penicillins, such as penicillin G. Another example is ezetimibe, a strong cholesterol absorption inhibitor and clinically proven reductant of plasma low-density lipoprotein fraction (LDL-C).^1^ In turn, orlistat, containing a β-lactone ring, has found use as a lipase inhibitor and as a drug prescribed for weight loss.^2^ These compounds, containing β-lactam and β-lactone rings as structural scaffolds, clearly show through clinical trials their important role as pharmaceutically interesting motifs.

**Scheme 1:**
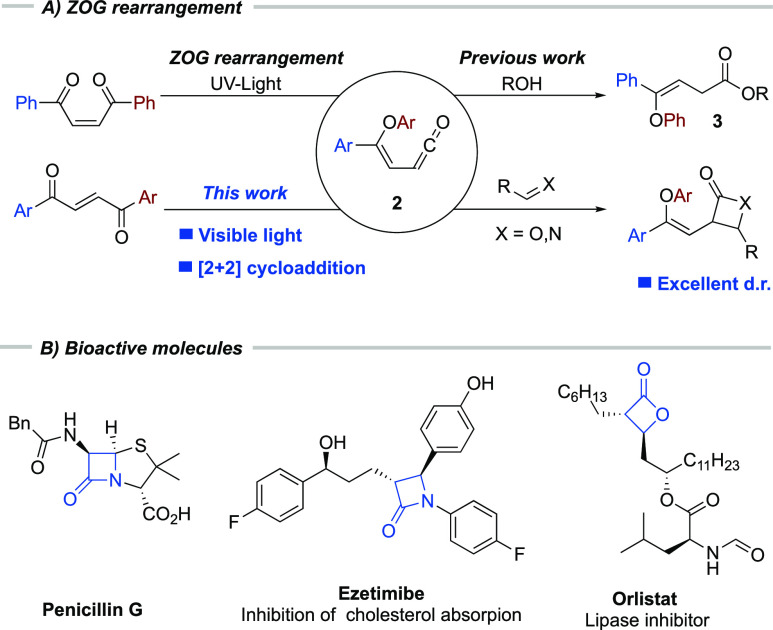
Previous Work on the ZOG Rearrangement and Biologically Active Candidates

Given the high importance of these classes of compounds, their syntheses have been thoroughly investigated. Most commonly, β-lactams are synthesized using the Staudinger synthesis, an imine-ketene [2 + 2] cycloaddition, and a range of different methods relying on this reaction have been developed.^3,4^ Ketenes are reactive compounds that typically are formed in situ from the α-deprotonation of acyl chlorides using a stoichiometric amount of base. This limits the structure of the ketenes and the reaction conditions available for the synthesis of β-lactams. An alternative method of producing ketenes *in situ* in a highly efficient manner is the photoinduced rearrangement of the dibenzoyl ethylene scaffold (**1** in Scheme 1A), which was first discovered by Zimmerman, O’Connell, and Griffin in the 1960s (Scheme 1A).^5−7^ Despite thorough mechanistic investigations, this rearrangement has only been utilized in the synthesis of 4-oxo-butanoic esters and acids (**3** in Scheme 1A) to date.^8−12^ Therefore, using the Zimmerman–O’Connell–Griffin (ZOG) rearrangement in combination with imines for the synthesis of substituted β-lactams is a novel and attractive strategy but potentially challenging. Typically, high-energy UV light is employed to carry out the ZOG rearrangement,^5−7^ and under these reaction conditions, several potential side reactions can occur, such as imine *E/Z*-isomerization and imine cycloadditions.^13,14^ A method relying on visible light could circumvent this problem, thereby making the ZOG rearrangement a powerful tool for the construction of four-membered rings under mild conditions.

Inspired by these opportunities, we initiated the study by choosing the model reaction between (*E*)-1,2-dibenzoyl ethylene **4** and the imine **5**. It is well established that upon irradiation with visible light, (*E*)-1,2-dibenzoyl ethylene undergoes isomerization to the *Z*-isomer **1**.^15^ We, therefore, envisaged that utilizing a high-intensity blue LED lamp as the irradiation source would make the isomerization go faster and be directly followed by the rearrangement reaction. To test this, a solution of 1,2-dibenzoyl ethylene (**4**) and imine **5** was irradiated with a 440 nm LED. After exploring various reaction conditions, it was discovered that the use of ethyl acetate as the solvent and a slight excess of the imine under ambient atmosphere led to the formation of the desired cycloaddition product **6** in 72% yield (Table 1, entry 4). While toluene did give a higher yield, the benefits of using a greener and more polar solvent made ethyl acetate the preferred choice for this reaction.^16,17^ To further optimize the reaction conditions, we tested different light sources. The blue LED (440 nm) was found to be effective in promoting both the isomerization and the rearrangement reaction, which led to the desired product formation in the optimized yield. However, the use of an LED with a longer wavelength, specifically 525 nm, did not promote the rearrangement reaction (Table 1, entry 5), which can be explained by the low absorbance of the (*Z*)-dibenzoyl ethylene at this wavelength (Figure S1).

**Table 1:**
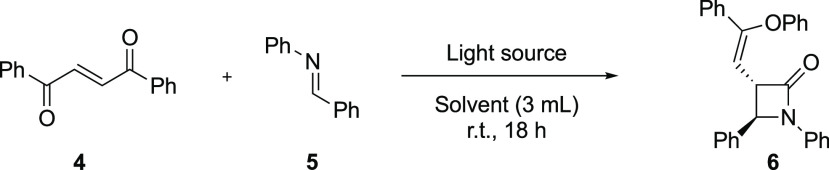
Optimization of the Photoinduced Synthesis of β-Lactams

entry	light source	solvent	**5** (equiv)	yield **6** (%)^a^
1	440 nm LED	ethyl acetate	1.0	78
2	440 nm LED	toluene	1.0	90
3	440 nm LED	acetonitrile	1.0	51
4	440 nm LED	ethyl acetate	1.2	86 (72^b^, 86^c^)
5	525 nm LED	ethyl acetate	1.2	n.d.

a Yield determined by ^1^H NMR using durene as the internal standard;

b Isolated yield.

c Conducted on 1 mmol scale.

With our optimized reaction conditions for the imine cyclization reaction obtained, a set of aryl–aryl imines was tested as reaction partners (Schemes 2 and 3). The introduction of electron-donating or electron-withdrawing groups in either the *para*, *meta*, or *ortho* position of the *N*-aryl group did not affect the reaction outcome significantly and gave the products **7**–**16** in 69–99% yield. Even the sterically demanding phenyl group in the *ortho* position was very well tolerated in the reaction and produced compound **16** in 89% yield. Imines with *N*-benzyl and *N*-butyl groups were also compatible with this methodology, which yielded both β-lactams **17** and **18** in 80% yield.

**Scheme 2:**
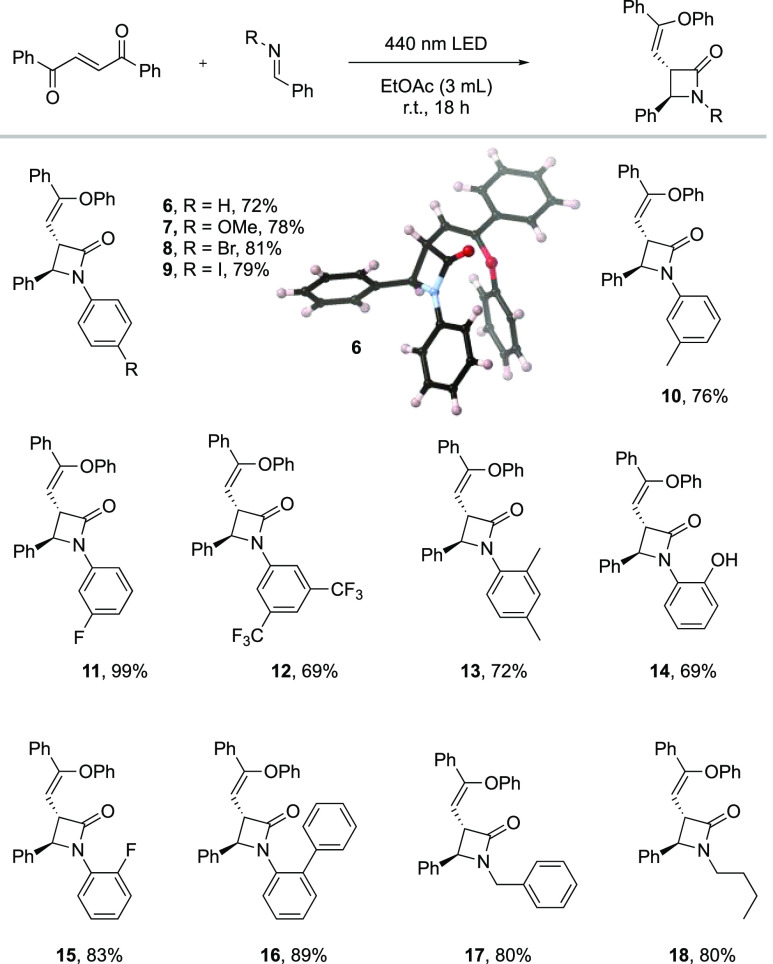
Scope of *N*-Substituted Phenyl Imines and Single Crystal X-ray Structure of **6** Reaction conditions: **4** (0.1 mmol) and imine (0.12 mmol) in EtOAc (3 mL), 440 nm LED, 18 h. Isolated yield.

**Scheme 3:**
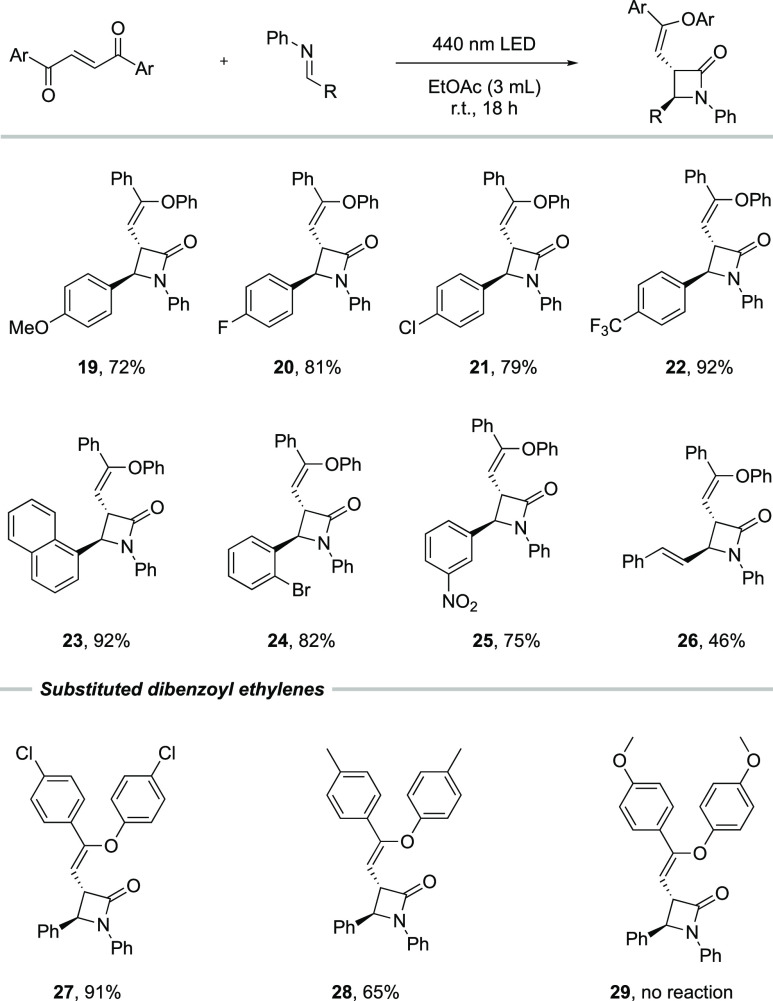
Scope of Substituted *N*-Phenyl Imines Reaction conditions: dibenzoyl ethylene (0.1 mmol) and imine (0.12 mmol) in EtOAc (3 mL), 440 nm LED, 18 h. Isolated yield.

It is noteworthy that the compatibility with phenols gave product **14** in 69% yield. This illustrates the excellent selectivity toward the cyclization reaction over nucleophilic attack of the ketene intermediate by the phenol.

When the aldehyde group of the imine was changed by introducing electron-withdrawing or -donating functional groups, the reaction outcome was not significantly affected, thereby affording the products **19**–**26** in 46–92% yield. Compound **26** was obtained selectively, albeit in a diminished yield of 46%, over the potential competing [4 + 2] cycloaddition, which showcases the selectivity of the reaction. Furthermore, the 1,2-dibenzoyl ethylene can also be varied. When 1,2-dibenzoyl ethylene was substituted with chlorine or methyl in the *para* position of the phenyl rings, products **27** and **28** could be obtained in good yields. However, introduction of the strongly electron-donating methoxy group suppressed the reaction completely with no conversion of the 1,2-dibenzoyl ethylene starting material. This observation can be explained by the fact that the bis-*para*-methoxy dibenzoyl ethylene does not undergo isomerization from the *E*- to the *Z*-isomer under the present conditions. The use of unsymmetrical 1,2-dibenzoyl ethylenes, that is, where two different aryl groups on the alkene were installed, was shown to result in sluggish reaction mixtures with no selectivity of the aryl transfer. To further investigate this reactivity, the simpler nucleophile methanol was attempted, which resulted in similar results (for discussion, see the Supporting Information).

All the β-lactam products were obtained as single diastereomers, and the orientation of the substituents on the four-membered rings was determined to be in an *anti*-configuration by solving the crystal structure of compound **6** using single crystal X-ray diffraction (Scheme 2).

To investigate the generality of our efficient method for the synthesis of β-lactams, we turned our attention to the reaction between 1,2-dibenzoyl ethylene and benzaldehydes. However, when we attempted the additive-free reaction between **4** and 3-nitro benzaldehyde under the conditions developed for the synthesis of β-lactams (Table S4, entry 1), no product was obtained. The addition of a Brönsted acid did not improve the results (Table S4, entry 2). However, we achieved successful synthesis of targeted lactone **30** with an 85% yield by employing boron trifluoride etherate and a suitable quenching procedure using triethylamine (Table S4, entry 4).

With an optimized procedure, different benzaldehydes were tested as substrates in the reaction (Scheme 4). Generally, electron-poor benzaldehydes gave the desired cycloaddition product in good to excellent yields (entries **30**–**33**), except for *para*-nitro benzaldehyde. Electron-rich benzaldehydes, however, were not tolerated and either did not take part in the cycloaddition reaction or decomposed rapidly via decarboxylation to yield the diene **34** in the case of *para*-*tert*-butyl benzaldehyde. The stereochemical outcome of the reaction was determined by single-crystal X-ray diffraction of compound **30**, and it was found that the lactones are formed with *syn* stereochemistry (Scheme 4).

**Scheme 4:**
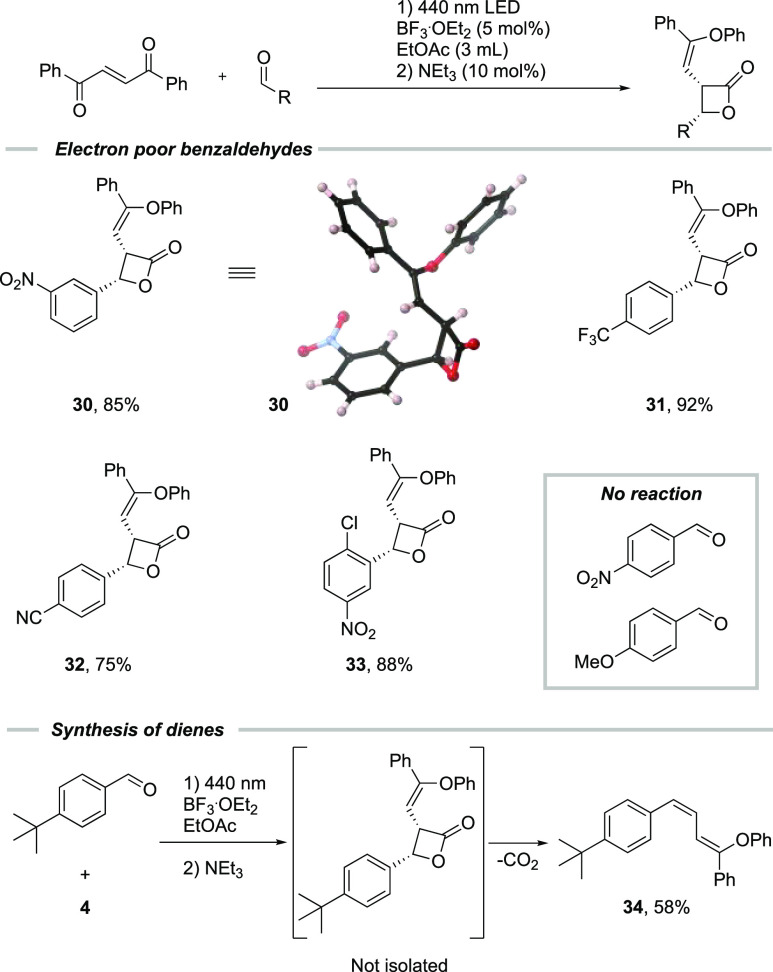
Scope of Substituted Benzaldehydes and Single Crystal X-ray Structure of **30** Reaction conditions: (1) 1,2-dibenzoyl ethylene (0.1 mmol), substituted aldehyde (0.12 mmol), and BF_3_·OEt_2_ (5 mol %), 440 nm LED, 18 h under N_2_; (2) Et_3_N (10 mol %), 30 min. Isolated yield.

To showcase the synthetic utility of the protocol, the reaction was performed on a 1 mmol scale to furnish model product **6** in 86% yield (Table 1, entry 4). Compound **6** was then further modified (Scheme 5). First, the phenoxy moiety could be hydrolyzed to reveal ketone **35** in 85% yield. Reduction of this compound with sodium borohydride gave alcohol **36** as a mixture of diastereomers in 78% yield. Compound **36** bears a structural resemblance to the pharmaceutically active drug ezetimibe, and the two-step procedure developed in this work could be used to produce a variety of analogues of this drug (Scheme 1B). Hydrogenolysis of compound **35** with Pd/C and H_2_ provided phenylpropylene amide **37**. The phenylpropylene backbone of **37** can be found in drugs such as trandolapril.^18^

**Scheme 5:**
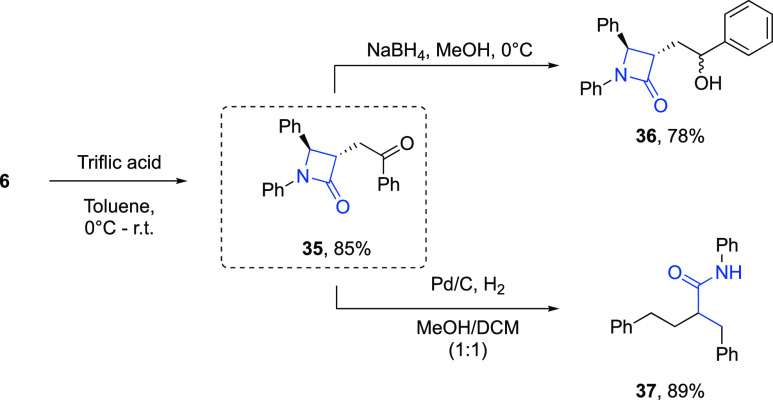
Further Transformations

The mechanism of the formation of the intermediate ketene has been thoroughly investigated in previous reports of this reaction and follows the pathway presented in Scheme 6. The *E*-1,2-dibenzoyl ethylene **4** is first isomerized to the *Z*-1,2-dibenzoyl ethylene **1**, which upon further excitation undergoes an intramolecular *ipso* attack of one of the oxygens to one of the phenyl rings.^5,6,20,7−12,15,19^ The formed spiro radical **I** collapses to form the ketene intermediate **2**, which is intercepted by an imine to form the final product via the Staudinger synthesis.^21,22^ To further support the formation of the ketene **2**, ethanol was used as a nucleophile, thereby resulting in the formation of the butanoate ester (for discussion, see the Supporting Information).

**Scheme 6:**
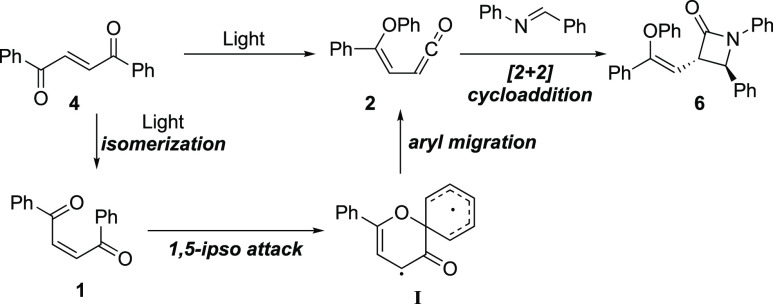
Proposed Mechanism

In summary, the photomediated 1,5-aryl rearrangement of 1,2-dibenzoyl ethylenes, known as the Zimmerman–O’Connell–Griffin (ZOG) rearrangement, has proven to be a highly efficient method for synthesizing β-lactams and β-lactones. The developed protocol offers a readily accessible pathway to substituted β-lactams and β-lactones with excellent diastereoselectivity utilizing commercially available starting materials. The reported protocol demonstrates high yields and operational simplicity, and the diastereoselectivity is supported by the analysis of single-crystal X-ray diffraction of two representative examples. Considering the significance of β-lactams and β-lactones in medicinal chemistry, we firmly believe that this reaction holds significant potential, for instance, in the preparation of combinatorial libraries.

## Data Availability

The data underlying this study are available in the published article and its Supporting Information.

## Supporting Information Available

Experimental details and NMR spectra of synthesized compounds (PDF)
