# Supplementary material for: Photochemical Access to Substituted β-Lactams and β-Lactones via the Zimmerman–O’Connell–Griffin Rearrangement
Source: Org Lett. 2023 Jul 18;25(29):5520–4. doi: 10.1021/acs.orglett.3c01990 (PMC10391623; doi:10.1021/acs.orglett.3c01990)
Supplement: Supplementary file 1 — ol3c01990_si_001.pdf [file ol3c01990_si_001.pdf]

Supporting Information

Photochemical access to substituted β-lactams and β-lactones via the Zimmerman-O'Connell-Griffin rearrangement

August Runemark[a], Mario Martos[b], Martin Nigrini[c], Francoise M. Amombo Noa[a], Lars Öhrström[a], Henrik Sundén[a,d*]

[a]*Department of Chemistry and Chemical Engineering, Chalmers University of Technology, Kemivägen 10, 412 96 Gothenburg, Sweden*

[b]*Organic Chemistry Department and Institute of Organic Synthesis (ISO), University of Alicante, ctra. San Vicente del Raspeig s/n, 03690 Alicante, Spain*

[c]*Department of Organic Chemistry, Faculty of Science, Charles University, Hlavova 2030, 128 43 Prague, Czech Republic*

[d] *Department of Chemistry and Molecular Biology, University of Gothenburg, Kemivägen 10, 412 96, Gothenburg, Sweden*

** E-mail: henrik.sunden@chem.gu.se.*

Table of contents

General information S3

UV-vis spectra of components and reaction mixture S4

Emission spectrum of irradiation source S5

Single crystal X-ray diffraction S6

Trapping of the ketene intermediate with ethanol S10

Unsuccessful substrates S12

Investigation of unsymmetrical 1,2-dibenzoyl ethylenes S13

General procedure for the optimization of the photoinduced synthesis of β-lactams. S15

General procedure for the synthesis of β-lactams S16

Table S4. Optimization of the synthesis of β-lactones S30

General procedure for the synthesis of β-lactones S31

Further transformations S35

NMR spectra of products S38

References S75

General information

All reagents and solvents were purchased from Sigma-Aldrich and Alfa Aesar and used without any further purification unless specified notice. Purifications were performed using an automated column chromatography system Biotage Isolera™ Spektra One with Biotage SNAP®-10 g KP-silica columns together with a 1 g samplet® cartridge using n-heptane or petroleum ether (40–60 °C)/ethyl acetate as solvent mixture, or using preparative TLC (SiO_2 60 micron, 20x20 cm) with pentane/ethyl acetate as the solvent system. ^1H (400 MHz) and ^{13}C (101 MHz) NMR spectra were acquired on an Agilent NMR machine at 25 °C. The chemical shifts for ^1H and ^{13}C NMR spectra are reported in parts per million (ppm) relative to the residual peak from solvent $CDCl_3$ as the internal standard; ^1H NMR at δ 7.26 ppm and ^{13}C NMR at δ 77.16 ppm. All coupling constants (*J*) are reported in Hertz (Hz) and multiplicities are indicated by s (singlet), d (doublet), dd (doublet of doublet), td (triplet of doublet), ddd (doublet of doublets of doublets), triplet (t), dt (doublet of triplet), and m (multiplet). Infrared (IR) spectra were recorded on a Bruker ATR FT-IR Spectrometer and are reported in wavenumber (cm^{-1}). High resolution mass spectrometry (HRMS) measurements were performed by CMSI service at Chalmers University of Technology using an Agilent QTOF 6520 equipped with an electrospray interface operated in positive ionization mode. UV-Vis absorption spectra were recorded on a Cary 4000 UV/Vis spectrometer, using 1x1 cm quartz cuvettes. All light promoted reactions were carried out in Biotage microwave vials (2-5 mL) under irradiation with a Kessil PR160L-440 LED lamp (λ_{max} 440 nm). Gas chromatography studies were performed using an Agilent 7820A equipped with a flame ionization detector and an Agilent HP-5 19091J-413 column. Emission spectra of light sources were measured using an AvaSpec-2048-2. Melting points were determined using a Büchi B-545 and are not corrected.

UV-vis spectra of components and reaction mixture

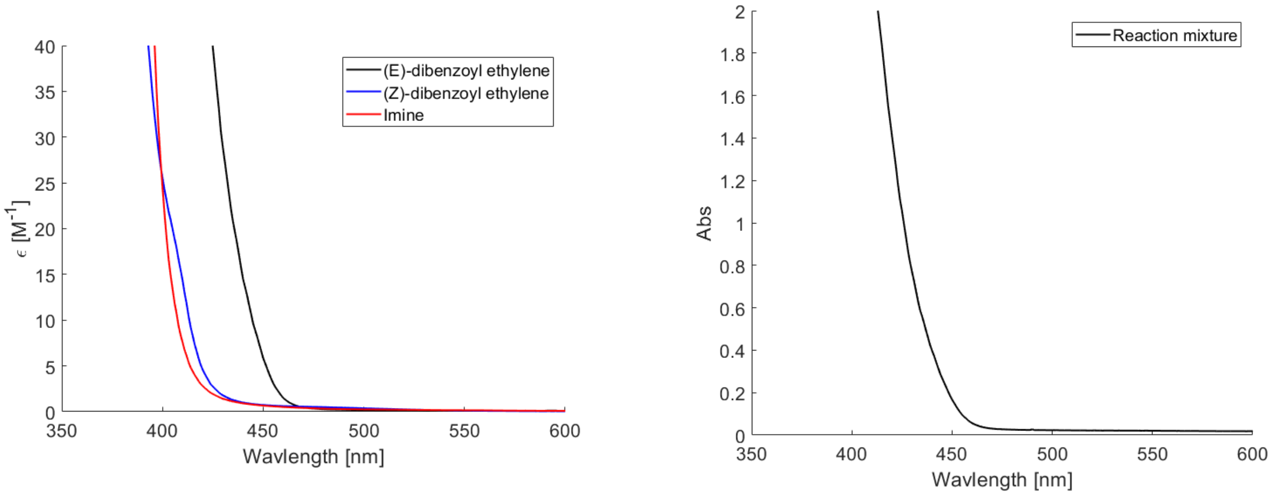

Figure S1. Left: Absorbance normalized to concentration of (*E*)-dibenzoyl ethylene (33 mM), (*Z*)-dibenzoyl ethylene (33 mM) and *N*-benzylideneaniline (41 mM). Measured in 1x1 cm quartz cuvettes in ethyl acetate. Right: Absorbance of the model reaction mixture in ethyl acetate.

Emission spectrum of irradiation source

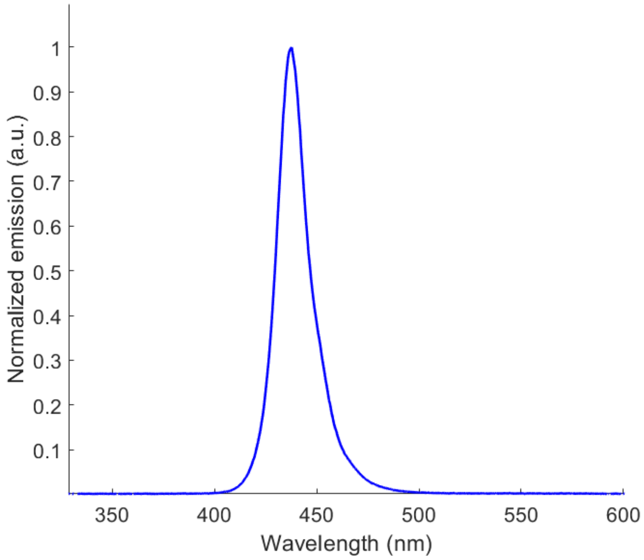

Figure S2. Normalized emission of the 440 nm Kessil LED (λ_{max} = 438 nm).

Single crystal X-ray diffraction

Compound 6

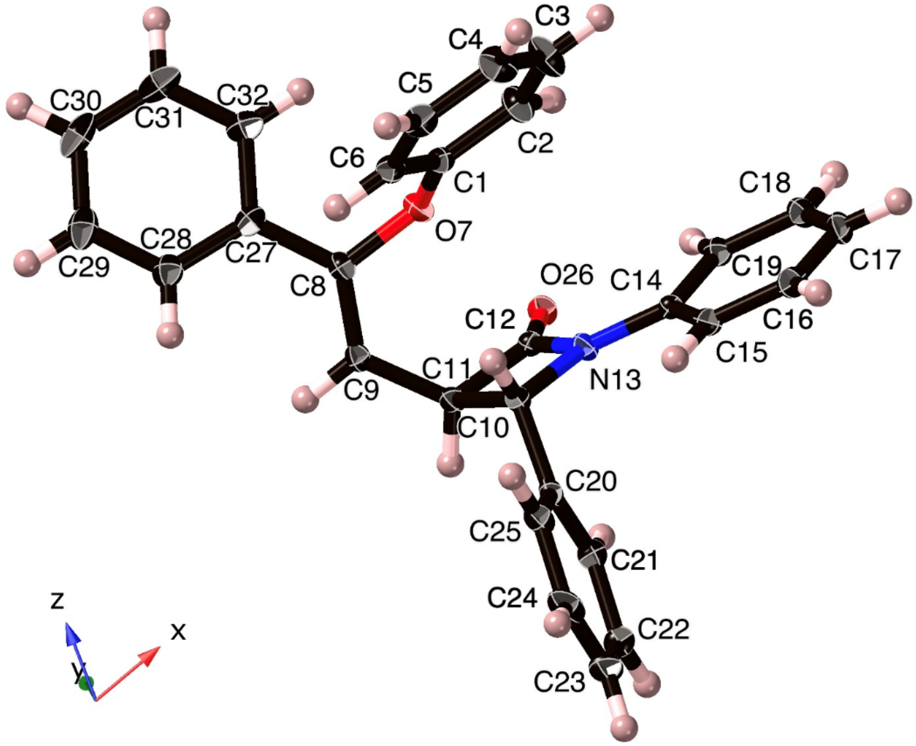

Experimental. colorless single block-shaped crystals of compound **6** were obtained by crystallization from n-pentane/EtOAc (20:1). A suitable crystal (0.05×0.04×0.03 mm) was selected and mounted on a support on an XtaLAB Synergy R, HyPix diffractometer. The crystal was kept at a steady *T* = 100.0(7) K during data collection. The structure was solved with the ShelXT[1] structure solution program using the Intrinsic Phasing solution method and by using **Olex2**[2] as the graphical interface. The model was refined with version 2016/6 of ShelXL 2016/6[1] using Least Squares minimization. Displacement ellipsoids are drawn at 50% probability level.

Table S1. Crystal data for compound **6**

**Compound**	**6**
Formula	$C_{29}H_{23}NO_2$
$D_{calc.}$/ g cm^{-3}	1.278
μ/mm^{-1}	0.628
Formula Weight	417.48
Colour	clear light colourless
Shape	block
Size/mm^3	0.05×0.04×0.03
T/K	100.0(7)
Crystal System	triclinic
Space Group	*P-1*
a/Å	9.3566(3)
b/Å	11.2225(4)
c/Å	11.7464(4)
α/°	68.784(3)
β/°	71.022(3)
γ/°	86.816(3)
V/Å3	1084.62(7)
Z	2
Z'	1
Wavelength/Å	1.54184
Radiation type	Cu K_{α}
Θ_{min}/°	4.236
Θ_{max}/°	75.402
Measured Refl.	20361
Independent Refl.	4330
Reflections with I > 2(I)	3703
Rint	0.0353
Parameters	305
Restraints	0
Largest Peak	0.207
Deepest Hole	-0.227
GooF	1.039
wR_2 *(all data)*	0.0829
wR_2	0.0792
R_1 *(all data)*	0.0414
R_1	0.0336

Compound 30

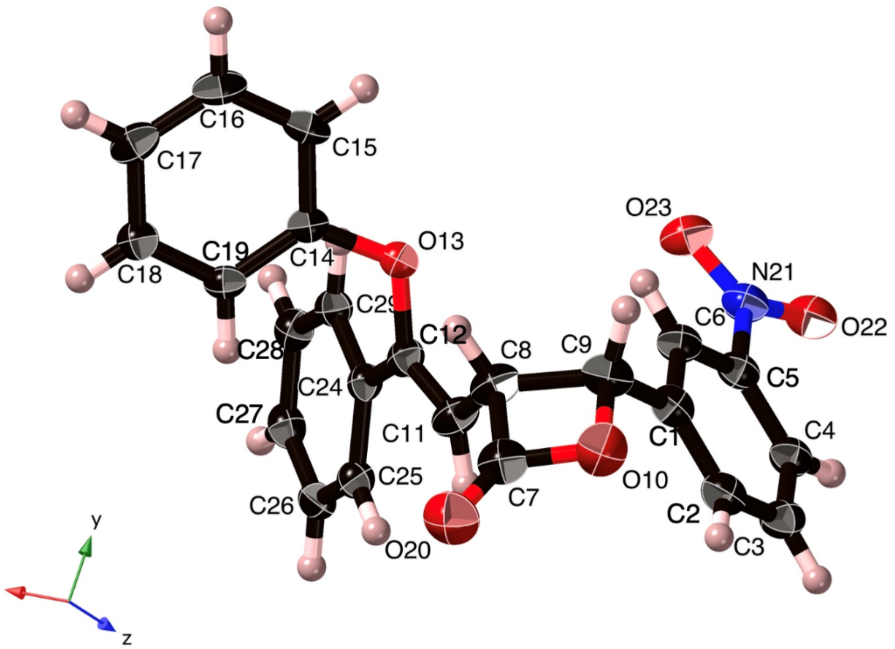

Experimental. Single clear whiteish colorless plate-shaped crystals of **30** were obtained from crystallization from n-pentane/EtOAc (20:1). A suitable crystal 0.15×0.03×0.01 mm3 was selected and mounted on a support on an XtaLAB Synergy R, HyPix diffractometer. The crystal was kept at a steady T = 102(4) K during data collection. The structure was solved with the ShelXT[1] structure solution program using the Intrinsic Phasing solution method and by using Olex2[2] as the graphical interface. The model was refined with version 2016/6 of ShelXL 2016/6[1] using Least Squares minimization. Displacement ellipsoids are drawn at 50% probability level.

Table S2. Crystal data for compound **30**

**Compound**	**30**
Formula	$C_{23}H_{17}NO_5$
$D_{calc.}$/ g cm^{-3}	1.400
μ/mm^{-1}	0.821
Formula Weight	387.38
Colour	clear whiteish colourless
Shape	plate
Size/mm^3	0.15×0.03×0.01
T/K	102(4)
Crystal System	monoclinic
Space Group	*P21/c*
a/Å	19.2412(10)
b/Å	5.7559(3)
c/Å	17.8102(9)
α/°	90
β/°	111.292(6)
γ/°	90
V/Å3	1837.85(18)
Z	4
Z'	1
Wavelength/Å	1.54184
Radiation type	Cu K[illegible]
Θ_{min}/°	4.934
Θ_{max}/°	78.267
Measured Refl.	18436
Independent Refl.	3681
Reflections with I > 2(I)	2642
Rint	0.0840
Parameters	274
Restraints	0
Largest Peak	0.293
Deepest Hole	-0.251
GooF	1.047
wR_2 *(all data)*	0.1482
wR_2	0.1331
R_1 *(all data)*	0.0793
R_1	0.0525

Trapping of the ketene intermediate with ethanol

To further support the formation of the proposed intermediate ketene (**2**), ethanol was used as a nucleophile under the optimized conditions (Scheme S1). The outcome of this reaction was the formation of the butanoate ester (**S1**), in accordance with the seminal investigations by Zimmerman.[3] This confirms that the reaction proceeds *via* the formation of the ketene (**2**).

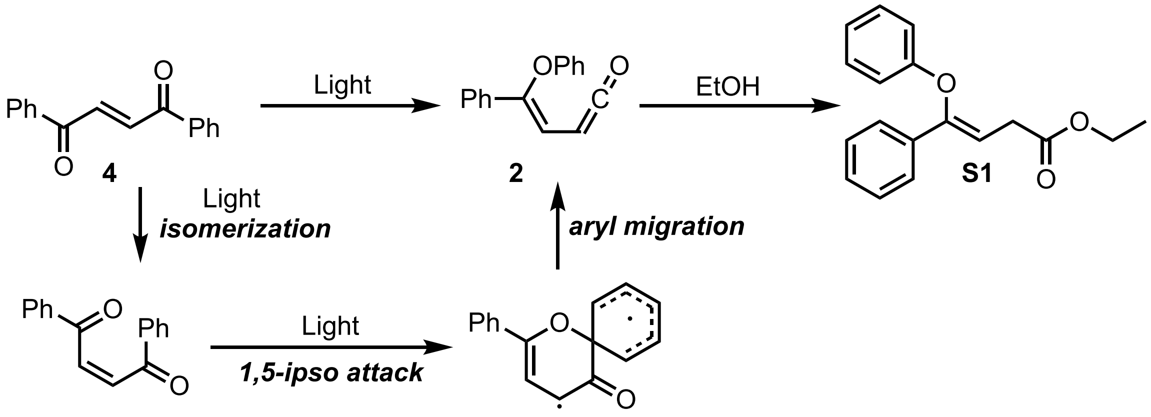

Scheme S1: Proposed mechanism for the formation of **S1**

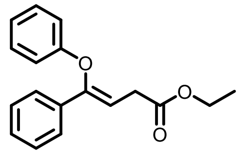

Compound **S1** was obtained after column chromatography (0-10% ethyl acetate in petroleum ether) as a colorless oil (20.1 mg, 72%). Spectroscopic data was in accordance with the literature[3]; **^{1}H NMR (400 MHz, $CDCl_3$)** δ = 7.56 – 7.46 (m, 3H), 7.34 – 7.17 (m, 8H), 6.06 (t, *J* = 7.0 Hz, 1H), 4.13 (q, *J* = 7.1 Hz, 3H), 3.29 (d, *J* = 7.0 Hz, 3H), 1.33 – 1.18 (m, 3H) ppm; **^{13}C NMR (101 MHz, $CDCl_3$)** δ = 171.7, 157.0, 151.0, 134.7, 129.7, 128.6, 128.6, 125.8, 121.9, 115.8, 109.5, 61.0, 31.7, 14.3 ppm.

NMR spectra of compound **S1**.

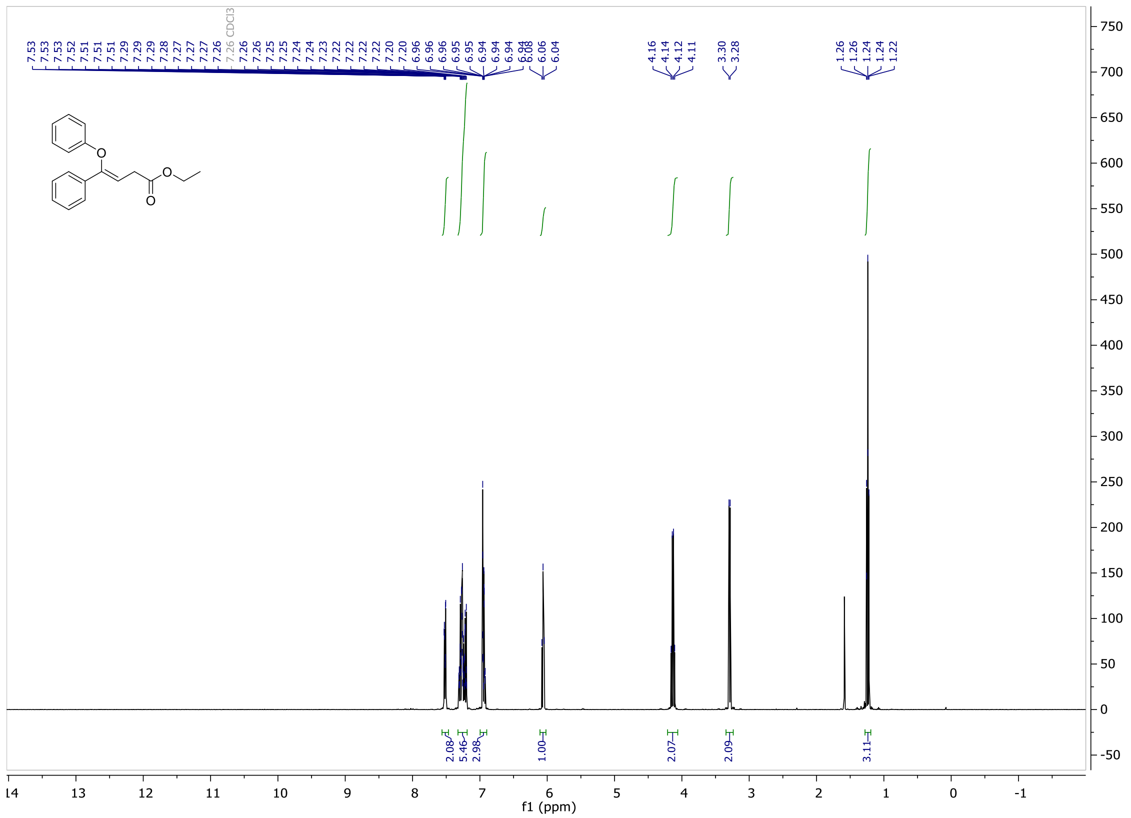

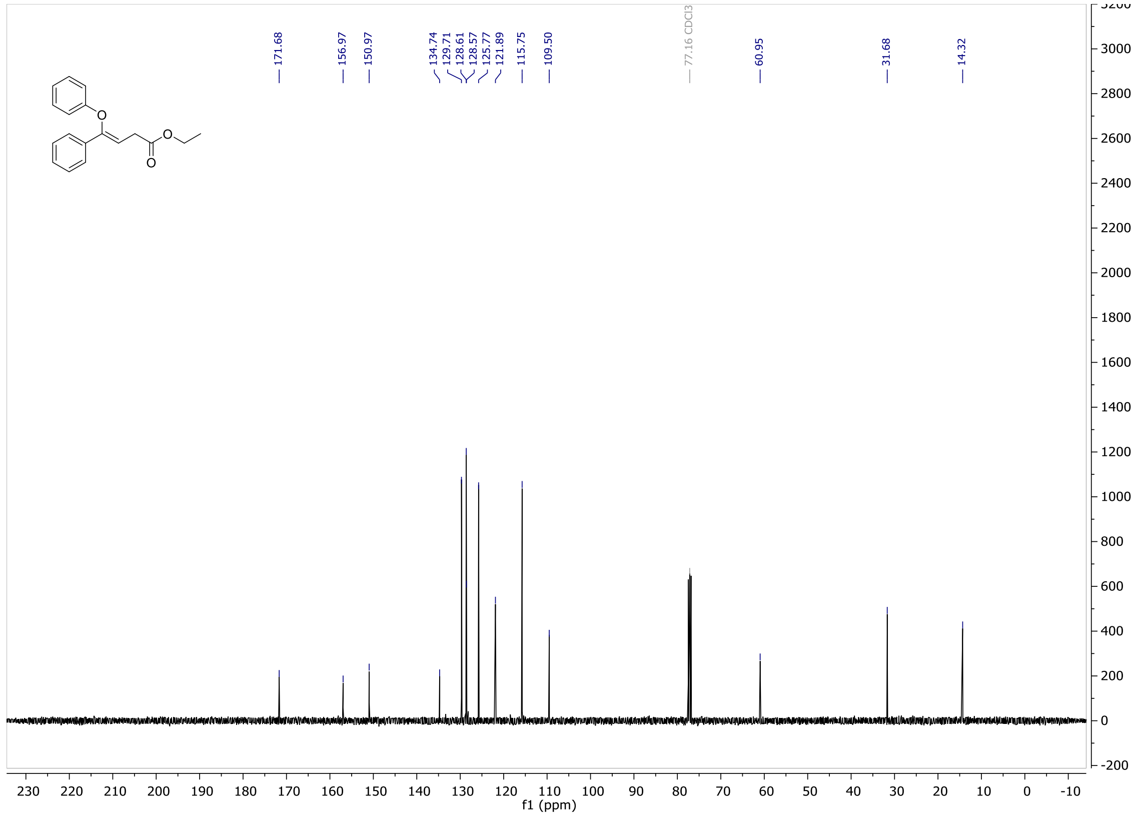

Unsuccessful substrates

Several imines and aldehydes were attempted as substrates for the synthesis of β-lactams and β-lactones under our optimized conditions without successful outcome. The substrates presented in Figure S3 resulted in either no conversion of the imine or a sluggish mixture of unidentified products.

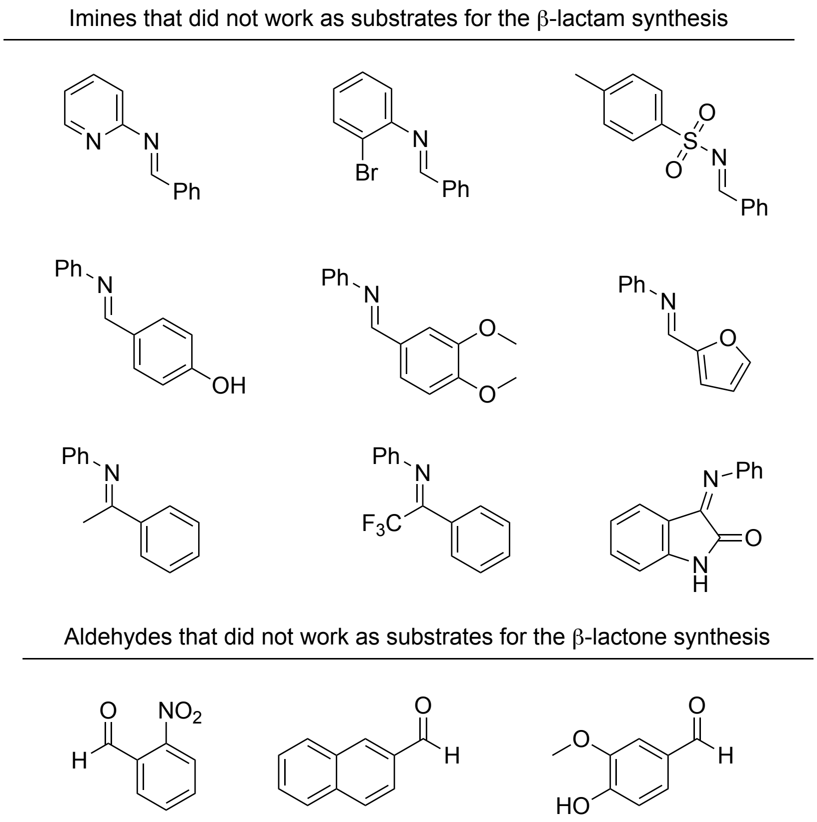

Figure S3. Unsuccessful substrates attempted in the synthesis of β-lactams and β-lactones.

Investigation of unsymmetrical 1,2-dibenzoyl ethylenes

1,2-Dibenzoyl ethylenes equipped with two differently substituted phenyl were attempted under the optimized conditions using imine **5**, however resulting in a sluggish reaction mixture with very low to no selectivity regarding the aryl transfer. To further investigate this reaction, the simpler nucleophile methanol was used in combination with OMe-H-1,2-dibenzoyl ethylene (**S2**) to exclude any influence from the imine (Scheme S1). This experiment also resulted in a reaction with no selectivity with esters (**S3** and **S3'**) obtained in a 1:1 ratio. Furthermore, when the alkene Br-H-1,2-dibenzoyl ethylene (**S4**) was used, the esters **S5** and **S5'** were formed in 1:1 ratio. This result indicates that the aryl transfer is not sensitive to electronic effects.

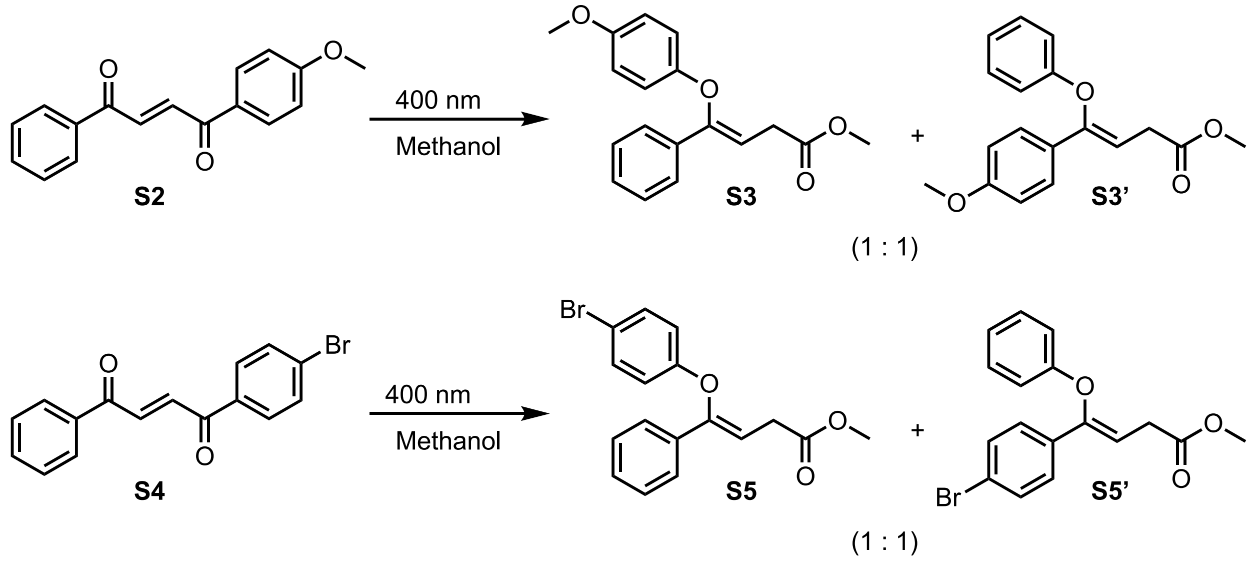

Scheme S2. Reaction between unsymmetrical 1,2-dibenzoyl ethylenes with methanol under optimized conditions.

^{1}H NMR of the mixture of **S3** and **S3'**.

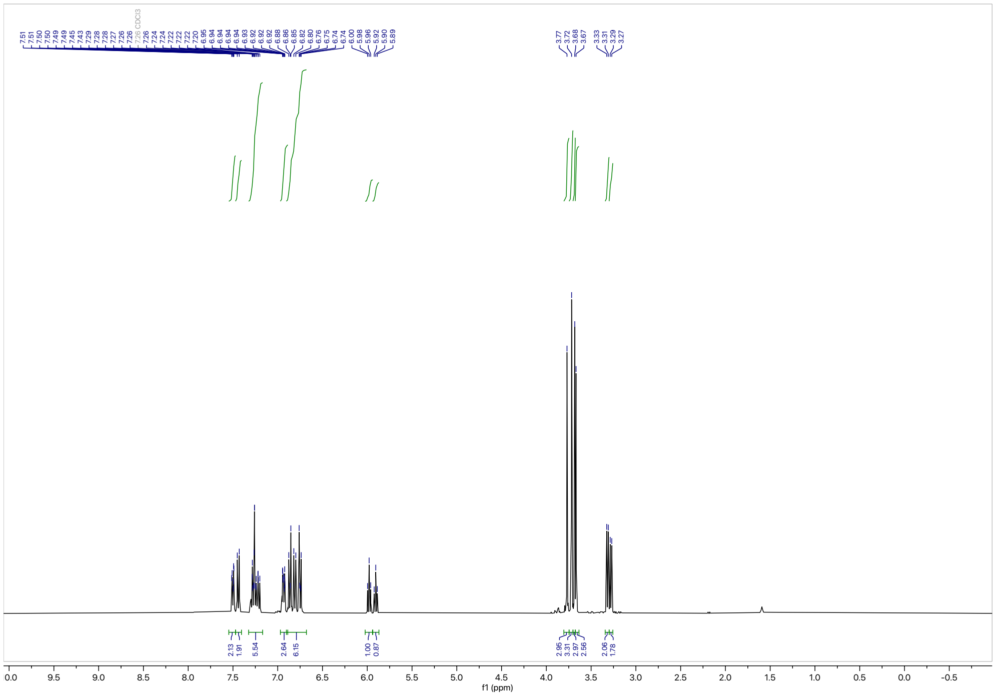

^{1}H NMR of the mixture of **S5** and **S5'**.

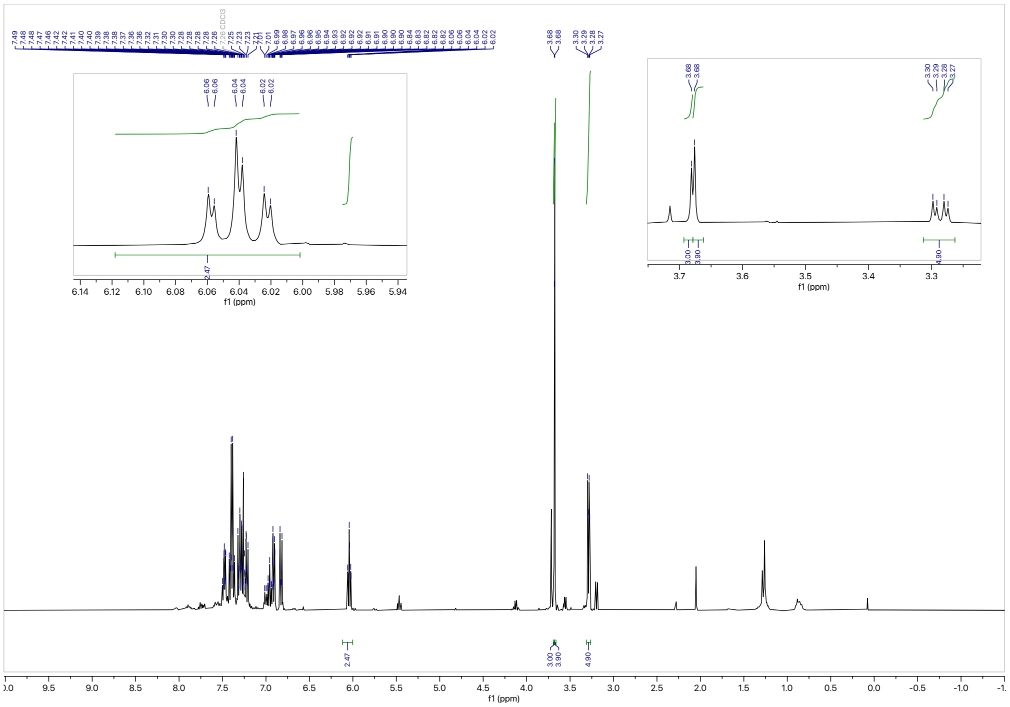

General procedure for the optimization of the photoinduced synthesis of β-lactams.

To a 2-5 mL Biotage microwave vial equipped with a magnetic stirrer was added (*E*)-1,4-diphenylbut-2-ene-1,4-dione (0.1 mmol), corresponding imine (1.0 or 1.2 equivalents) and solvent (3 mL). The flask was irradiated with a light source (440 nm or 525 nm LED) and stirred for 18 hours. The reaction mixture was concentrated *in vacuo* and to the crude residue was added durene as internal standard prior to ^{1}H NMR analysis in $CDCl_3$ to determine the NMR yield of the reaction. Yields from different reaction conditions are presented in Table S3.

Table S3. Optimization of the photoinduced synthesis of β-lactams.

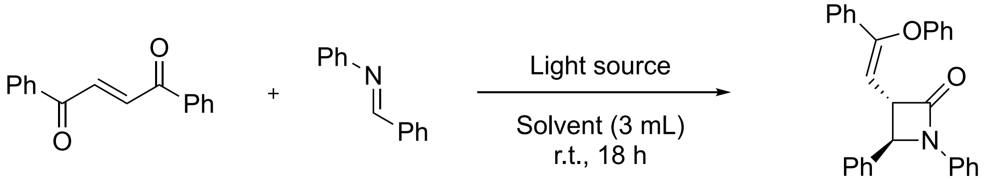

4 **5** **6**

Entry	Light source	Solvent	Equiv. **5**	Yield (%) **6**
1	440 nm LED	Ethyl acetate	1.0	78
2	440 nm LED	Toluene	1.0	90
3	440 nm LED	Acetonitrile	1.0	51
4	440 nm LED	Ethyl acetate	1.2	86 (72[b])
5	525 nm LED	Ethyl acetate	1.2	n.d.[c]

a) Yield determined by ^{1}H NMR using durene as the internal standard; b) isolated yield; c) product not detected.

General procedure for the synthesis of β-lactams

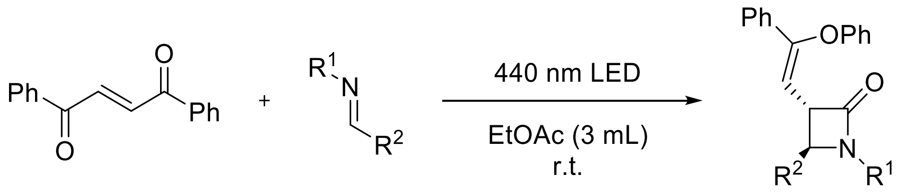

To a 2-5 mL Biotage microwave vial equipped with a magnetic stirrer was added (*E*)-1,4-diphenylbut-2-ene-1,4-dione (0.1 mmol, 1 equiv.), corresponding imine (0.12 mmol, 1.2 equiv.) and ethyl acetate (0.03 M). The flask was irradiated with a Kessil 440 nm LED and stirred until full conversion of starting material (monitored by GC-FID or TLC). The reaction mixture was concentrated *in vacuo* and separated on column chromatography or preparative TLC to obtain the products **6-28**.

Synthesis of compound 6 on 1 mmol scale

To a Schlenk tube equipped with a magnetic stirrer was added (*E*)-1,4-diphenylbut-2-ene-1,4-dione **4** (236 mg, 1.0 mmol, 1 equiv.), **5** (217 mg, 1.2 mmol, 1.2 equiv.) and ethyl acetate (30 mL). The flask was irradiated with a Kessil 440 nm LED and stirred until full conversion of starting material (monitored by TLC). The reaction mixture was concentrated *in vacuo* and separated using column chromatography (SiO_2, pentane/ethyl acetate 9:1) to obtain compound **6** (359 mg, 86%).

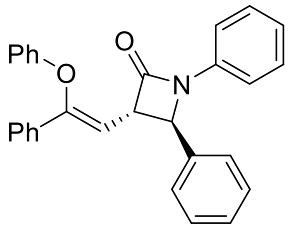

3-((Z)-2-phenoxy-2-phenylvinyl)-1,4-diphenylazetidin-2-one (6)

Purified by preparative column chromatography (pentane/ethyl acetate 9:1), colorless oil, 30 mg (72%).

^{1}H NMR (400 MHz, $CDCl_3$) δ = 7.54 – 7.45 (m, 2H), 7.35 – 7.19 (m, 12H), 7.14 (ddd, *J* = 9.1, 7.3, 2.0 Hz, 2H), 7.07 – 7.01 (m, 1H), 6.92 – 6.86 (m, 1H), 6.84 (dd, *J* = 7.6, 1.6 Hz, 2H), 6.07 (dd, *J* = 8.7, 1.8 Hz, 1H), 4.88 (t, *J* = 2.1 Hz, 1H), 4.17 (d, *J* = 8.7 Hz, 1H) ppm.

^{13}C NMR (101 MHz, $CDCl_3$) δ = 166.0, 156.8, 153.7, 137.8, 137.5, 129.6 (2C), 129.2 (2C), 129.1 (2C), 129.0, 128.7 (2C), 128.6, 126.2 (2C), 126.1 (2C), 124.1, 122.2, 117.2, 117.2 (2C), 116.2 (2C), 109.8, 62.2, 58.1 ppm.

HRMS m/z: $(M+H)^+$ Calcd. for $C_{29}H_{24}NO_2$ = 418.1807; **Found:** = 418.1807.

IR: 1735, 1653, 1591, 1559, 1540, 1488, 1457, 1380, 1327, 1215, 1155, 1118, 1074, 1045, 1025, 987, 937, 868, 750, 735, 688, 577, 506 cm^{-1}.

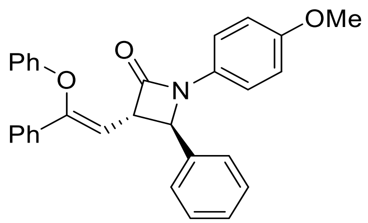

(Z)-1-(4-methoxyphenyl)-3-(2-phenoxy-2-phenylvinyl)-4-phenylazetidin-2-one (7).

Purified by preparative TLC (pentane/ethyl acetate 7:3), yellow solid, 35 mg (78%).

m.p. 125.8 – 126.5 °C

^{1}H NMR (400 MHz, CDCl$_3$) δ = 7.53-7.51 (m, 2H), 7.31-7.22 (m, 10H), 7.17-7.13 (m, 2H), 6.92-6.85 (m, 3H), 6.79-6.77 (m, 2H), 6.09 (d, *J* = 8.7 Hz, 1H), 4.86 (d, *J* = 2.4 Hz, 1H), 4.17 (dd, *J* = 8.7, 2.4 Hz, 1H), 3.74 (s, 3H) ppm.

^{13}C NMR (100 MHz, CDCl$_3$) δ = 165.3, 156.8, 156.1, 153.5, 137.4, 134.3, 131.3, 129.6 (2C), 129.0 (2C), 128.9, 128.7 (2C), 128.5, 126.1 (2C), 126.0 (2C), 122.1, 118.4 (2C), 116.2 (2C), 114.3 (2C), 109.9, 62.1, 57.9, 55.5 ppm.

HRMS m/z: (M+H)$^+$ Calcd. for $C_{30}H_{26}NO_3$ = 448.1913; **Found:** = 448.1915.

IR: 1734, 1700, 1685, 1653, 1636, 1617, 1595, 1540, 1513, 1489, 1472, 1456, 1438, 1394, 1299, 1240, 1212, 1183, 1162, 1114, 1070, 1043, 1025, 984, 867, 835, 805, 771, 745, 722, 690, 668, 569 cm^{-1}.

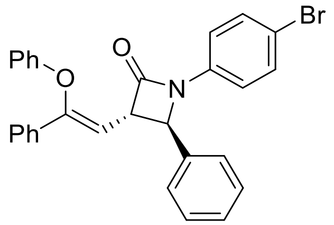

(Z)-1-(4-bromophenyl)-3-(2-phenoxy-2-phenylvinyl)-4-phenylazetidin-2-one (8).

Purified by column chromatography (pentane/ethyl acetate 9:1), yellow solid, 40 mg (81%).

m.p. 118.0 – 120.0 °C

^{1}H NMR (400 MHz, CDCl$_3$) δ = 7.50-7.48 (m, 2H), 7.34-7.23 (m, 10H), 7.15-7.11 (m, 4H), 6.90-6.86 (m, 1H), 6.82-6.80 (m, 2H), 6.04 (d, *J* = 8.7 Hz, 1H), 4.86 (d, *J* = 2.5 Hz, 1H), 4.16 (dd, *J* = 8.7, 2.5 Hz, 1H) ppm.

^{13}C NMR (100 MHz, CDCl$_3$) δ = 165.9, 156.7, 153.8, 136.9, 136.7, 134.2, 132.2 (2C), 129.6 (2C), 129.2 (2C), 129.1, 128.7 (3C), 126.10 (2C), 126.09 (2C), 122.2, 118.7 (2C), 116.7, 116.2 (2C), 109.3, 62.3, 58.3 ppm.

HRMS m/z: $(M+H)^+$ Calcd. for $C_{29}H_{23}BrNO_2$ = 496.0912; **Found:** = 496.0911.

IR: 1734, 1685, 1653, 1590, 1540, 1521, 1507, 1488, 1457, 1446, 1418, 1378, 1326, 1219, 1153, 1115, 1073, 1026, 990, 936, 910, 886, 818, 752, 739, 707 cm^{-1}.

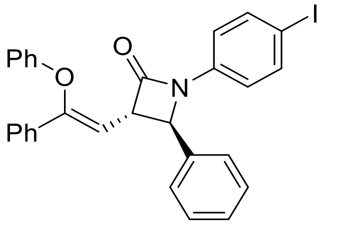

(Z)-1-(4-iodophenyl)-3-(2-phenoxy-2-phenylvinyl)-4-phenylazetidin-2-one (9).

Purified by column chromatography (pentane/ethyl acetate 97:3), yellow oil, 43 mg (79%).

^{1}H NMR (400 MHz, CDCl$_3$) δ = 7.54-7.49 (m, 4H), 7.32-7.23 (m, 8H), 7.15-7.11 (m, 2H), 7.04-7.01 (m, 2H), 6.91-6.87 (m, 1H), 6.83-6.81 (m, 2H), 6.05 (d, *J* = 8.7 Hz, 1H), 4.86 (d, *J* = 2.5 Hz, 1H), 4.16 (dd, *J* = 8.7, 2.5 Hz, 1H) ppm.

^{13}C NMR (100 MHz, CDCl$_3$) δ = 165.9, 156.7, 153.8, 138.1 (2C), 137.3, 136.9, 134.2, 129.6 (2C), 129.2 (2C), 129.1, 128.7 (3C), 126.10 (2C), 126.08 (2C), 122.2, 119.1 (2C), 116.2 (2C), 109.3, 87.3, 62.2, 58.3 ppm.

HRMS m/z: $(M+H)^+$ Calcd. for $C_{29}H_{23}INO_2$ = 544.0773; **Found:** = 544.0771.

IR: 3854, 3082, 1700, 1685, 1670, 1559, 1507, 1489, 1473, 1457, 1437, 1419, 668 cm^{-1}.

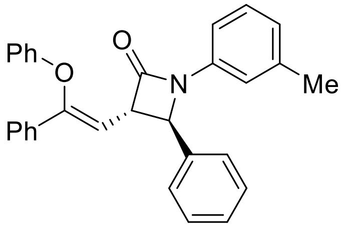

(Z)-3-(2-phenoxy-2-phenylvinyl)-4-phenyl-1-(m-tolyl)azetidin-2-one (10).

Purified by preparative TLC (pentane/ethyl acetate 9:1), white solid, 33 mg (76%).

m.p. 124.0 – 124.3 °C

^{1}H NMR (400 MHz, CDCl$_3$) δ = 7.53-7.50 (m, 2H), 7.29-7.27 (m, 9H), 7.17-7.09 (m, 3H), 6.95-6.84 (m, 5H), 6.08 (d, *J* = 8.7 Hz, 1H), 4.86 (d, *J* = 2.2 Hz, 1H), 4.15 (dd, *J* = 8.7, 2.2 Hz, 1H), 2.28 (s, 3H) ppm.

^{13}C NMR (100 MHz, CDCl$_3$) δ = 165.9, 156.8, 153.5, 139.2, 137.7, 137.5, 134.3, 129.6 (2C), 129.1 (2C), 129.0, 128.9, 128.7 (2C), 128.5, 126.09 (2C), 126.07 (2C), 124.9, 122.1, 118.1, 116.2 (2C), 114.0, 109.9, 62.1, 57.9, 21.6 ppm.

HRMS m/z: $(M+H)^+$ Calcd. for $C_{30}H_{26}NO_2$ = 432.1964; **Found:** = 432.1964.

IR: 1742, 1653, 1589, 1488, 1447, 1381, 1332, 1256, 1212, 1151, 1114, 1070, 1025, 988, 889, 865, 770, 749, 730, 687 cm^{-1}.

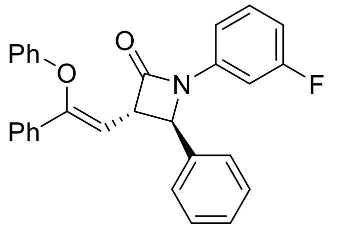

(Z)-1-(3-fluorophenyl)-3-(2-phenoxy-2-phenylvinyl)-4-phenylazetidin-2-one (11).

Purified by column chromatography (pentane/ethyl acetate 95:5), yellow solid, 43 mg (99%).

m.p. 123.8 – 125.0 °C

^{1}H NMR (400 MHz, CDCl$_3$) δ = 7.51-5.48 (m, 2H), 7.31-7.25 (m, 8H), 7.17-7.04 (m, 3H), 7.06 (dt, *J* = 10.3, 2.2 Hz, 1H), 6.97-6.96 (m, 1H), 6.90-6.87 (m, 1H), 6.83-6.80 (m, 2H), 6.73 (tdd, *J* = 8.4, 2.5, 0.6 Hz, 1H), 6.04 (d, *J* = 8.7 Hz, 1H), 4.86 (d, *J* = 2.6 Hz, 1H), 4.17 (dd, *J* = 8.7, 2.6 Hz, 1H) ppm.

^{13}C NMR (100 MHz, CDCl$_3$) δ = 166.1, 163.0 (d, $^1J_{C-F}$ = 245.9 Hz), 156.7, 153.8, 139.1 (d, $^3J_{C-F}$ = 10.4 Hz), 136.9, 134.2, 130.5 (d, $^3J_{C-F}$ = 9.3 Hz), 129.6 (2C), 129.2 (2C), 129.1, 128.7 (2C), 126.09 (2C), 126.08 (2C), 122.2, 116.2 (2C), 112.6 (d, $^4J_{C-F}$ = 3.0 Hz), 110.9 (d, $^2J_{C-F}$ = 21.3 Hz), 109.3 (2C), 104.8 (d, $^2J_{C-F}$ = 25.9 Hz), 62.5, 58.2 ppm.

^{19}F NMR (564 MHz, CDCl$_3$) δ = -111.00 (dddd, *J* = 10.0, 8.5, 6.4, 1.0 Hz) ppm.

HRMS m/z: $(M+H)^+$ Calcd. for $C_{29}H_{23}FNO_2$ = 436.1713; **Found:** = 436.1711.

IR: 3870, 1749, 1718, 1700, 1636, 1576, 1489, 1457, 1387, 1209, 1155, 867, 747, 690 cm^{-1}.

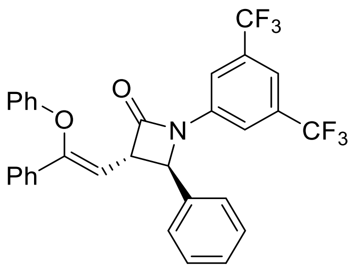

(Z)-1-(3,5-bis(trifluoromethyl)phenyl)-3-(2-phenoxy-2-phenylvinyl)-4-phenylazetidin-2-one (12).

Purified by preparative TLC (pentane/ethyl acetate 9:1), yellow oil, 38 mg (69%). The product was obtained contaminated with a small amount of water.

^{1}H NMR (400 MHz, CDCl$_3$) δ = 7.65 (s, 2H), 7.52-7.49 (m, 3H), 7.36-7.26 (m, 8H), 7.15 (m, 2H), 6.92-6.88 (m, 1H), 6.81 (m, 2H), 6.04 (d, J = 8.6 Hz, 1H), 4.98 (d, J = 2.7 Hz, 1H), 4.26 (dd, J = 8.6, 2.7 Hz, 1H) ppm.

^{13}C NMR (100 MHz, CDCl$_3$) δ = 166.5, 156.6, 154.3, 138.9, 136.0, 133.9, 132.6 (q, $^{2}J_{C\text{-}F}$ = 33.6 Hz, 2C), 129.6 (2C), 129.5 (2C), 129.3, 129.2, 128.8 (2C), 126.13 (2C), 126.06 (2C), 122.9 (q, $^{1}J_{C\text{-}F}$ = 272.9 Hz, 2C), 117.2-117.1 (m), 116.8-116.7 (m, 2C), 116.1 (2C), 108.5 (2C), 62.8, 58.6 ppm.

^{19}F NMR (564 MHz, CDCl$_3$) δ = -63.17 – (-63.18) (m).

HRMS m/z: (M+H)$^+$ Calcd. for $C_{31}H_{22}F_6NO_2$ = 554.1555; **Found:** = 554.1553.

IR: 1758, 1619, 1593, 1490, 1474, 1402, 1276, 1213, 1179, 1131, 1070, 1047, 1026, 907, 881, 844, 728, 697, 649, 507 cm^{-1}.

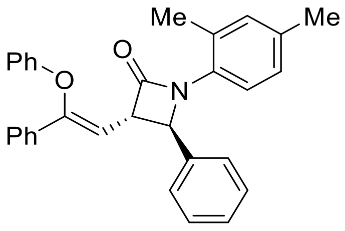

(Z)-1-(2,4-dimethylphenyl)-3-(2-phenoxy-2-phenylvinyl)-4-phenylazetidin-2-one (13).

Purified by preparative TLC (pentane/ethyl acetate 4:1), yellow oil, 32 mg (72%).

^{1}H NMR (400 MHz, CDCl$_3$) δ = 7.55-7.53 (m, 2H), 7.33-7.22 (m, 8H), 7.17-7.13 (m, 2H), 7.02 (d, J = 8.0 Hz, 1H), 6.97 (br s, 1H), 6.91-6.86 (m, 4H), 6.13 (d, J = 8.7 Hz, 1H), 5.05 (d, J = 2.5 Hz, 1H), 4.24 (dd, J = 8.7, 2.5 Hz, 1H),2.37 (s, 3H), 2.24 (s, 3H) ppm.

^{13}C NMR (100 MHz, CDCl$_3$) δ = 166.4, 156.8, 153.4, 137.8, 136.3, 134.4, 132.4, 132.3, 132.1, 129.6 (2C), 128.9, 128.8 (2C), 128.7 (2C), 128.4, 127.1, 126.5 (2C), 126.1 (2C), 122.7, 122.1, 116.2 (2C), 110.2, 63.6, 56.7, 21.0, 19.5 ppm.

HRMS m/z: (M+H)$^+$ Calcd. for $C_{31}H_{28}NO_2$ = 446.2120; **Found:** = 446.2124.

IR: 1747, 1592, 1504, 1488, 1456, 1373, 1333, 1213, 1165, 1120, 1074, 1025, 984, 907, 818, 720, 691, 647 cm^{-1}.

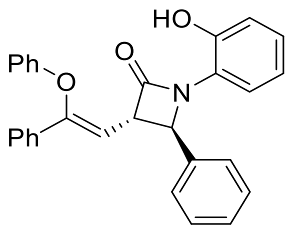

(Z)-1-(2-hydroxyphenyl)-3-(2-phenoxy-2-phenylvinyl)-4-phenylazetidin-2-one (14).

Purified by preparative TLC (pentane/ethyl acetate 4:1), yellow solid, 30 mg (69%).

m.p. 153.3 – 155.0 °C

^{1}HNMR (400 MHz, $CDCl_3$) δ = 9.73 (br s, 1H), 7.54-7.51 (m, 2H), 7.33-7.26 (m, 8H), 7.17-7.13 (m, 2H), 7.05-7.00 (m, 2H), 6.93-6.90 (m, 1H), 6.85-6.83 (m, 2H), 6.63 (ddd, *J* = 8.4, 6.1, 2.6 Hz, 1H), 6.45 (d, *J* = 7.7 Hz, 1H), 6.03 (d, *J* = 8.6 Hz, 1H), 4.98 (d, *J* = 2.2 Hz, 1H), 4.15 (dd, *J* = 8.6, 2.2 Hz, 1H) ppm.

^{13}CNMR (100 MHz, $CDCl_3$) δ = 167.6, 156.6, 154.4, 147.8, 136.2, 134.0, 129.7 (2C), 129.2 (3C), 128.9, 128.8 (2C), 126.8, 126.2 (2C), 126.1 (2C), 125.4, 122.3, 119.8, 119.1, 117.9, 116.2 (2C), 108.6, 62.1, 54.7 ppm.

HRMS m/z: $(M+H)^+$ Calcd. for $C_{29}H_{24}NO_3$ = 434.1756; **Found:** = 434.1757.

IR: 3061, 1700, 1589, 1559, 1490, 1452, 1386, 1333, 1276, 1250, 1211, 1148, 1135, 1079, 1027, 983, 929, 861, 778, 750, 735 cm^{-1}.

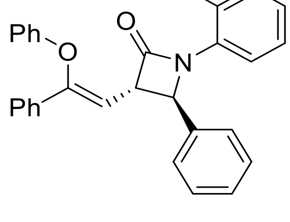

(Z)-1-(2-fluorophenyl)-3-(2-phenoxy-2-phenylvinyl)-4-phenylazetidin-2-one (15).

Purified by preparative TLC (pentane/ethyl acetate 9:1), yellow oil, 36 mg (83%).

^{1}H NMR (400 MHz, $CDCl_3$) δ = 7.92 (td, *J* = 7.9, 1.7 Hz, 1H), 7.54-7.52 (m, 2H), 7.32-7.25 (m, 8H), 7.17-7.09 (m, 3H), 7.06-7.01 (m, 1H), 6.97-6.85 (m, 4H), 6.12 (d, *J* = 8.8 Hz, 1H), 5.19 (dd, *J* = 3.6, 2.5 Hz, 1H), 4.26 (dd, *J* = 8.8, 2.5 Hz, 1H) ppm.

^{13}C NMR (100 MHz, CDCl$_3$) δ = 166.5, 156.8, 153.5, 152.9 (d, $^1J_{C-F}$ = 247.5 Hz), 138.2, 134.3, 129.6 (2C), 129.0, 128.8 (2C), 128.7 (2C), 128.4, 126.1 (2C), 126.0 (2C), 125.8 (d, $^3J_{C-F}$ = 7.4 Hz), 124.7 (d, $^4J_{C-F}$ = 3.5 Hz), 124.6 (d, $^3J_{C-F}$ = 11.2 Hz), 123.0 (d, *J* = 2.5 Hz), 122.1, 116.6 (d, $^2J_{C-F}$ = 19.4 Hz), 116.2 (2C), 109.8, 64.8 (d, $^4J_{C-F}$ = 4.7 Hz), 58.9 ppm.

^{19}F NMR (564 MHz, CDCl$_3$) δ = -125.92 (ddt, *J* = 11.8, 8.2, 4.3 Hz) ppm.

HRMS m/z: $(M+H)^+$ Calcd. for $C_{29}H_{23}FNO_2$ = 436.1713; **Found:** = 436.1713.

IR: 3034, 1751, 1653, 1591, 1504, 1488, 1457, 1373, 1331, 1212, 1164, 1120, 1068, 1043, 1025, 985, 907, 859 cm^{-1}.

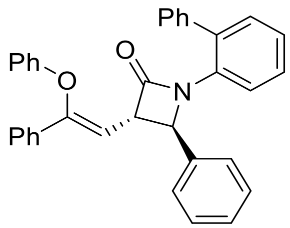

(Z)-1-([1,1'-biphenyl]-2-yl)-3-(2-phenoxy-2-phenylvinyl)-4-phenylazetidin-2-one (16).

Purified by recrystallization (methanol), white solid, 44 mg (89%)

m.p. 209.5 – 210.2 °C

^{1}H NMR (400 MHz, CDCl$_3$) δ = 7.73 (dd, *J* = 8.0, 0.6 Hz, 1H), 7.48-7.42 (m, 5H), 7.35-7.08 (m, 13H), 6.88-6.84 (m, 1H), 6.79-6.75 (m, 4H), 5.86 (d, *J* = 8.8 Hz, 1H), 4.11 (dd, *J* = 8.8, 2.3 Hz, 1H), 3.97 (d, *J* = 2.3 Hz, 1H) ppm.

^{13}C NMR (100 MHz, CDCl$_3$) δ = 167.5, 156.7, 153.0, 139.3, 137.4, 135.9, 134.4, 133.3, 131.1, 129.53 (2C), 129.51 (2C), 128.9, 128.7 (2C), 128.5 (2C), 128.4 (2C), 128.3, 128.2, 127.9, 126.63 (2C), 126.6, 126.1 (2C), 125.7, 122.1, 116.2 (2C), 110.1, 64.5, 57.7 ppm.

HRMS m/z: $(M+H)^+$ Calcd. for $C_{35}H_{28}NO_2$ = 494.2120; **Found:** = 494.2121.

IR: 1750, 1653, 1595, 1477, 1454, 1436, 1378, 1352, 1332, 1216, 1156, 1117, 1069, 1023, 984, 865, 784, 751, 729, 707, 693, 582, 556 cm^{-1}.

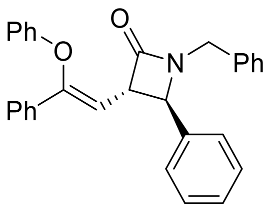

(Z)-1-benzyl-3-(2-phenoxy-2-phenylvinyl)-4-phenylazetidin-2-one (17).

Purified by column chromatography (pentane/ethyl acetate 9:1), white solid, 32 mg (80%). The isolated product contains trace amount (4%) of inseparable *(Z)*-1,2-dibenzoyl ethylene (7.94 ppm, 4H, integrated to 0.18 relative to the product).

m.p. 103.3 – 104.0 °C

^{1}H NMR (400 MHz, $CDCl_3$): δ = 7.47-7.45 (m, 2H), 7.31-7.23 (m, 9H), 7.16-7.07 (m, 6H), 6.86-6.84 (m, 1H), 6.79-6.77 (m, 2H), 5.95 (dd, *J* = 8.8, 0.9 Hz, 1H), 4.83 (d, *J* = 15.0 Hz, 1H), 4.29 (d, *J* = 1.5 Hz, 1H), 4.11 (m, 1H), 3.77 (d, *J* = 15.0 Hz, 1H) ppm.

^{13}C NMR (100 MHz, $CDCl_3$) δ = 168.4, 156.7, 153.1, 137.1, 135.6, 134.4, 129.5 (2C), 128.90 (2C), 128.87 (2C), 128.8, 128.62 (2C), 128.59 (2C), 128.5, 127.8, 126.7 (2C), 126.0 (2C), 122.0, 116.2 (2C), 110.0, 61.5, 57.9, 44.7 ppm.

HRMS m/z: $(M+H)^+$ Calcd. for $C_{30}H_{26}NO_2$ = 432.1964; **Found:** = 432.1962.

IR: 1739, 1654, 1590, 1488, 1456, 1394, 1336, 1287, 1231, 1212, 1183, 1117, 1074, 1007, 912, 872, 751, 728, 702, 689, 648, 587 cm^{-1}.

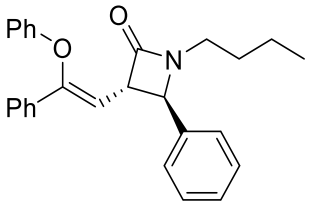

(Z)-1-butyl-3-(2-phenoxy-2-phenylvinyl)-4-phenylazetidin-2-one (18).

Purified by column chromatography (pentane/ethyl acetate 9:1), yellow oil, 32 mg (80%). The isolated product contains trace amount (3%) of inseparable *(Z)*-1,2-dibenzoyl ethylene (7.94 ppm, 4H, integrated to 0.17 relative to the product).

^{1}H NMR (400 MHz, $CDCl_3$) δ = 7.51-7.48 (m, 2H), 7.32-7.21 (m, 8H), 7.14-7.10 (m, 2H), 6.89 – 6.77 (m, 3H), 6.01 (d, *J* = 8.6 Hz, 1H), 4.43 (d, *J* = 2.0 Hz, 1H), 4.01 (dd, *J* = 8.6, 2.0 Hz, 1H), 3.48-3.45 (m, 1H), 2.84-2.80 (m, 1H), 1.45-1.39 (m, 2H), 1.32-1.26 (m, 2H), 0.87 (t, 3H, *J* = 7.2 Hz) ppm.

^{13}C NMR (100 MHz, CDCl$_3$) δ = 168.6, 156.8, 153.0, 137.7, 134.5, 129.5 (2C), 128.9 (2C), 128.8, 128.6 (2C), 128.5, 126.6 (2C), 126.0 (2C), 122.0, 116.2 (2C), 110.4, 62.1, 57.8, 40.5, 29.9, 20.3, 13.7 ppm.

HRMS m/z: $(M+H)^+$ Calcd. for $C_{27}H_{28}NO_2$ = 398.2120; **Found:** = 398.2121.

IR: 1744, 1592, 1488, 1456, 1399, 1277, 1212, 1165, 1119, 1074, 1024, 862, 749, 600 cm^{-1}.

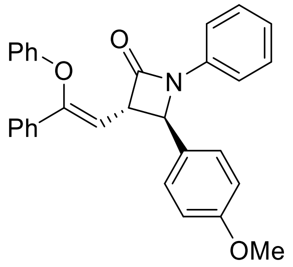

4-(4-methoxyphenyl)-3-((Z)-2-phenoxy-2-phenylvinyl)-1-phenylazetidin-2-one (19):

Purified by column chromatography (SiO_2, pentane/ethyl acetate 9:1), yellow solid, 32.2 mg (72%). The isolated product contains trace amount (5%) of inseparable *(Z)*-1,2-dibenzoyl ethylene (7.94 ppm, 4H, integrated to 0.22 relative to the product).

m.p. 135.5 – 136.4 °C

^{1}H NMR (400 MHz, CDCl$_3$): δ = 7.52 – 7.48 (m, 2H), 7.32 – 7.26 (m, 5H), 7.26 – 7.22 (m, 2H), 7.20 (dt, *J* = 9.7, 2.3 Hz, 2H), 7.17 – 7.12 (m, 2H), 7.06 – 7.00 (m, 1H), 6.93 – 6.87 (m, 1H), 6.84 (ddd, *J* = 8.8, 7.8, 1.5 Hz, 4H), 6.06 (d, *J* = 8.7 Hz, 1H), 4.84 (d, *J* = 2.4 Hz, 1H), 4.15 (dd, *J* = 8.7, 2.5 Hz, 1H), 3.78 (s, 3H) ppm.

^{13}C NMR (400 MHz, CDCl$_3$): δ = 166.1, 159.7, 156.9, 153.5, 137.8, 134.3, 129.6 (2C), 129.3, 129.1 (2C), 128.9, 128.7 (2C), 127.5 (2C), 126.1 (2C), 123.9, 122.1, 117.2 (2C), 116.2 (2C), 114.5 (2C), 109.9, 61.8, 58.1, 55.4 ppm.

HRMS m/z: $(M+H)^+$ Calcd. for $C_{30}H_{26}NO_3$ = 448.1913; **Found:** = 448.1911.

IR: 1738, 1653, 1616, 1593, 1559, 1540, 1515, 1501, 1487, 1458, 1448, 1381, 1337, 1308, 1258, 1221, 1174, 1153, 1130, 1093, 1034, 983, 937, 895, 781, 750 cm^{-1}.

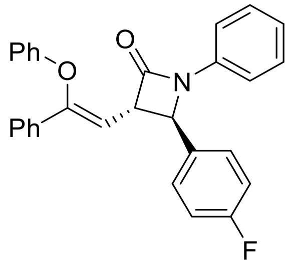

4-(4-fluorophenyl)-3-((Z)-2-phenoxy-2-phenylvinyl)-1-phenylazetidin-2-one (20):

Purified by column chromatography (SiO_2, pentane/ethyl acetate 9:1), yellow oil, 35.3 mg (81%).

^{1}H NMR (400 MHz, $CDCl_3$): δ = 7.55 – 7.47 (m, 2H), 7.33 – 7.27 (m, 4H), 7.26 – 7.21 (m, 5H), 7.19 – 7.12 (m, 2H), 7.06 (tt, *J* = 5.3, 3.1 Hz, 1H), 7.02 – 6.95 (m, 2H), 6.94 – 6.88 (m, 1H), 6.86 – 6.81 (m, 2H), 6.08 (d, *J* = 8.6 Hz, 1H), 4.87 (d, *J* = 2.5 Hz, 1H), 4.15 (dd, *J* = 8.5, 2.5 Hz, 1H) ppm.

^{13}C NMR (400 MHz, $CDCl_3$): δ = 165.8, 162.7 (d, $^1J_{C-F}$ = 247 Hz), 156.8, 153.7, 137.6, 134.2, 133.2 (d, $^4J_{C-F}$ = 3.1 Hz), 129.7 (2C), 129.2 (2C), 129.1, 128.7 (2C), 127.9 (d, $^3J_{C-F}$ = 8.3 Hz, 2C), 126.1 (2C), 124.2, 122.2, 117.1 (2C), 116.1 (d, $^2J_{C-F}$ = 22 Hz, 2C), 116.1 (2C), 109.5, 61.5, 58.1 ppm.

^{19}F NMR (564 MHz, $CDCl_3$) δ = -113.33 – (-113.37) (m).

HRMS m/z: $(M+H)^+$ Calcd. for $C_{29}H_{23}FNO_2$ = 536.1713; **Found:** = 436.1714.

IR: 1747, 1653, 1595, 1488, 1447, 1423, 1377, 1329, 1287, 1212, 1155, 1120, 1089, 1024, 984, 908, 839 cm^{-1}.

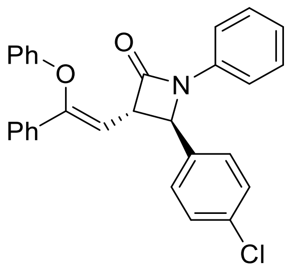

4-(4-chlorophenyl)-3-((Z)-2-phenoxy-2-phenylvinyl)-1-phenylazetidin-2-one (21):

Purified by column chromatography (SiO_2, pentane/ethyl acetate 9:1), yellow solid, 35.7 mg (79%).

m.p. 140.0 – 140.5 °C

^{1}H NMR (400 MHz, $CDCl_3$): δ = 7.52 – 7.47 (m, 2H), 7.32 – 7.21 (m, 9H), 7.20 – 7.11 (m, 4H), 7.04 (dt, *J* = 8.7, 4.2 Hz, 1H), 6.90 (t, *J* = 7.4 Hz, 1H), 6.85 – 6.79 (m, 2H), 6.05 (d, *J* = 8.6 Hz, 1H), 4.84 (d, *J* = 2.4 Hz, 1H), 4.13 (dd, *J* = 8.5, 2.5 Hz, 1H) ppm.

^{13}C NMR (400 MHz, $CDCl_3$): δ = 165.7, 156.8, 153.8, 137.5, 136.0, 134.3, 134.1, 129.7 (2C), 129.3 (2C), 129.2 (2C), 129.1, 128.7 (2C), 127.5 (2C), 126.1 (2C), 124.2, 122.2, 117.1 (2C), 116.1 (2C), 109.4, 61.4, 58.1 ppm.

HRMS m/z: $(M+H)^+$ Calcd. for $C_{29}H_{23}ClNO_2$ = 452.1417; **Found:** = 452.1417.

IR: 3854, 3745, 1844, 1772, 1734, 1717, 1521, 1507, 1457, 1220, 1091, 687 cm^{-1}.

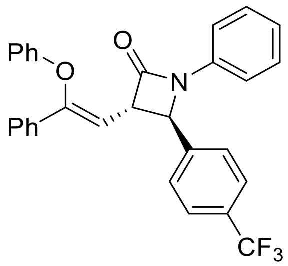

3-((Z)-2-phenoxy-2-phenylvinyl)-1-phenyl-4-(*p*-tolyl)azetidin-2-one (22):

Purified by column chromatography (SiO_2, pentane/ethyl acetate 9:1), yellow oil, 44.7 mg (92%). The isolated product contains trace amount (6%) of inseparable *(E)*-1,2-dibenzoyl ethylene (8.07 ppm, 4H, integrated to 0.29 relative to the product).

^{1}H NMR (400 MHz, $CDCl_3$): δ = 7.53 (d, *J* = 8.2 Hz, 2H), 7.49 (dt, *J* = 5.2, 3.2 Hz, 2H), 7.35 (d, *J* = 8.1 Hz, 2H), 7.31 – 7.26 (m, 3H), 7.22 (dd, *J* = 8.9, 2.2 Hz, 4H), 7.15 – 7.09 (m, 2H), 7.09 – 7.03 (m, 1H), 6.92 – 6.86 (m, 1H), 6.82 – 6.77 (m, 2H), 6.07 (d, *J* = 8.5 Hz, 1H), 4.91 (d, *J* = 2.5 Hz, 1H), 4.18 – 4.08 (m, 1H) ppm.

^{13}C NMR (400 MHz, $CDCl_3$): δ = 165.5, 156.8, 154.1, 141.6, 137.4, 134.1, 134.0, 129.7 (2C), 129.3 (2C), 129.2, 129.06 (d, $^4J_{C\text{-}F}$ = 1.9 Hz), 128.8 (2C), 126.5 (2C), 126.1 (q, $^3J_{C\text{-}F}$ = 3.8 Hz, 2C), 126.1 (2C), 124.4, 122.3, 117.1 (2C), 116.1 (2C), 109.1, 61.5, 58.1 ppm.

^{19}F NMR (564 MHz, $CDCl_3$) δ = -62.66– (-62.67) (m).

HRMS m/z: $(M+H)^+$ Calcd. for $C_{30}H_{23}F_3NO_2$ = 486.1681; **Found:** = 486.1681.

IR: 1752, 1653, 1620, 1596, 1489, 1447, 1422, 1378, 1323, 1212, 1164, 1121, 1067, 1017, 982, 849, 749, 689, 606 cm^{-1}.

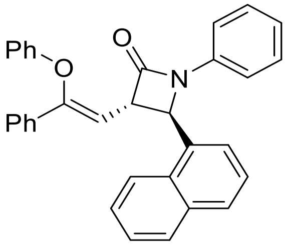

4-(naphthalen-1-yl)-3-((Z)-2-phenoxy-2-phenylvinyl)-1-phenylazetidin-2-one (23).

Purified by column chromatography (SiO_2, pentane/ethyl acetate 9:1), yellow solid, 43 mg (92 %).

m.p. 153.0 – 155.0 °C

^{1}H NMR (400 MHz, $CDCl_3$): δ = 7.84 – 7.81 (m, 1H), 7.79 (d, *J* = 8.9 Hz, 1H), 7.76 – 7.72 (m, 1H), 7.70 (d, *J* = 1.7 Hz, 1H), 7.54 – 7.46 (m, 4H), 7.39 (dd, *J* = 8.5, 1.8 Hz, 1H), 7.34 – 7.27 (m, 5H), 7.25 – 7.19 (m, 2H), 7.12

– 7.06 (m, 2H), 7.05 – 7.00 (m, 1H), 6.87 – 6.79 (m, 3H), 6.11 (d, *J* = 8.5 Hz, 1H), 5.04 (d, *J* = 2.5 Hz, 1H), 4.27 (dd, *J* = 8.5, 2.5 Hz, 1H) ppm.

^{13}C NMR (101 MHz, $CDCl_3$): δ = 166.0, 156.8, 153.7, 137.9, 134.9, 134.3, 133.5, 133.3, 129.6 (2C), 129.3, 129.2 (3C), 129.0, 128.7 (2C), 128.1, 127.9, 126.6, 126.5, 126.1, 125.9, 124.1, 123.2, 122.2, 117.2 (2C), 116.2 (2C), 109.7, 62.4, 58.1 ppm.

HRMS m/z: $(M+H)^+$ Calcd. for $C_{33}H_{26}NO_2$ = 468.1964; **Found:** = 468.1964.

IR: 1685, 1675, 1647, 1636, 1540, 1507, 1327, 1211, 1156, 1118, 1024, 898, 824, 750 cm^{-1}.

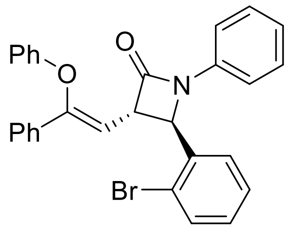

4-(2-bromophenyl)-3-((Z)-2-phenoxy-2-phenylvinyl)-1-phenylazetidin-2-one (24):

Purified by column chromatography (SiO_2, pentane/ethyl acetate 9:1), yellow oil, 40.7 mg (82%)

^{1}H NMR (400 MHz, $CDCl_3$): δ = 7.60 (d, *J* = 7.7 Hz, 1H), 7.54 – 7.49 (m, 2H), 7.32 – 7.24 (m, 7H), 7.24 – 7.19 (m, 2H), 7.18 – 7.04 (m, 4H), 6.91 – 6.82 (m, 3H), 6.08 (d, *J* = 9.8 Hz, 1H), 5.37 (d, *J* = 2.6 Hz, 1H), 4.12 (dd, *J* = 9.8, 2.6 Hz, 1H) ppm.

^{13}C NMR (400 MHz, $CDCl_3$): δ 165.6, 156.8, 153.6, 137.5, 136.5, 134.4, 133.3, 129.8, 129.5 (2C), 129.3 (2C), 128.9, 128.7 (2C), 128.2, 126.9, 126.3 (2C), 124.2, 122.7, 122.1, 117.2 (2C), 116.3 (2C), 110.1, 60.6, 57.4 ppm.

HRMS m/z: $(M+H)^+$ Calcd. for $C_{29}H_{23}BrNO_2$ = 496.0912; **Found:** = 496.0920.

IR: 3854, 1753, 1653, 1594, 1487, 1567, 1380, 1292, 1246, 1223, 1178, 1163, 1121, 1093, 1045, 1024, 988, 865, 767, 749, 737 cm^{-1}.

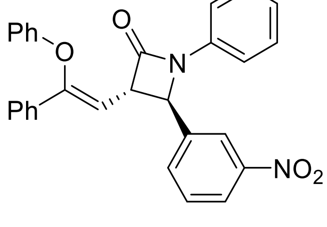

4-(3-nitrophenyl)-3-((*Z*)-2-phenoxy-2-phenylvinyl)-1-phenylazetidin-2-one (25):

Purified by column chromatography (SiO_2, pentane/ethyl acetate 9:1), yellow oil, 34.7 mg (75%).

^{1}H NMR (400 MHz, CDCl$_3$): δ = 8.16 – 8.10 (m, 2H), 7.59 (d, *J* = 7.8 Hz, 1H), 7.53 – 7.43 (m, 3H), 7.33 – 7.28 (m, 4H), 7.23 (dd, *J* = 6.1, 4.3 Hz, 3H), 7.16 – 7.04 (m, 3H), 6.86 (t, *J* = 7.4 Hz, 1H), 6.82 – 6.78 (m, 2H), 6.10 (d, *J* = 8.1 Hz, 1H), 4.97 (d, *J* = 2.5 Hz, 1H), 4.18 (dd, *J* = 8.1, 2.5 Hz, 1H) ppm.

^{13}C NMR (400 MHz, CDCl$_3$): δ = 165.4, 156.7, 154.3, 148.7, 139.9, 137.2, 133.9, 131.6, 130.3, 129.8 (2C), 129.4 (2C), 129.3, 128.8 (2C), 126.1 (2C), 124.6, 123.6, 122.4, 121.7, 117.0 (2C), 116.0 (2C), 108.7, 61.2, 58.2 ppm.

HRMS m/z: $(M+H)^+$ Calcd. for $C_{29}H_{23}N_2O_4$ = 463.1658; **Found:** = 463.1658.

IR: 1751, 1653, 1594, 1529, 1488, 1446, 1378, 1348, 1211, 1165, 1119, 1043, 981, 908, 808, 752, 731, 600, 504 cm^{-1}.

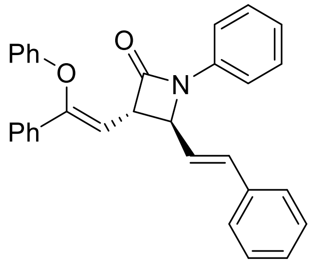

3-((*Z*)-2-phenoxy-2-phenylvinyl)-1-phenyl-4-((*E*)-styryl)azetidin-2-one (26):

Purified by column chromatography (SiO$_2$, pentane/ethyl acetate 95:5), yellow oil, 21.1 mg (46%).

^{1}H NMR (400 MHz, CDCl$_3$): δ = 7.54 – 7.49 (m, 2H), 7.47 – 7.41 (m, 2H), 7.29 (ddt, *J* = 8.2, 5.8, 2.3 Hz, 10H), 7.21 – 7.13 (m, 2H), 7.07 (t, *J* = 7.5 Hz, 1H), 6.97 – 6.92 (m, 2H), 6.90 (td, *J* = 7.4, 1.9 Hz, 1H), 6.61 (d, *J* = 15.9 Hz, 1H), 6.17 (ddt, *J* = 16.1, 8.3, 1.8 Hz, 1H), 6.06 (dt, *J* = 8.5, 1.8 Hz, 1H), 4.52 (dd, *J* = 8.1, 2.7 Hz, 1H), 4.19 (dt, *J* = 8.5, 1.8 Hz, 1H) ppm.

^{13}C NMR (400 MHz, CDCl$_3$): δ = 165.6, 157.1, 153.5, 138.3, 135.9, 134.3, 134.2, 129.8 (2C), 129.2 (2C), 129.1, 128.9, 128.7 (3C), 128.9, 126.8 (2C), 126.3, 126.0 (2C), 124.1, 122.2, 117.1 (2C), 116.2 (2C), 109.6, 61.2, 55.3 ppm.

HRMS m/z: $(M+H)^+$ Calcd. for $C_{31}H_{26}NO_2$ = 444.1964; **Found:** = 444.1965.

IR: 1745, 1653, 1596, 1488, 1447, 1379, 1328, 1212, 1165, 1074, 1042, 1024, 966, 907, 731, 680, 506 cm^{-1}.

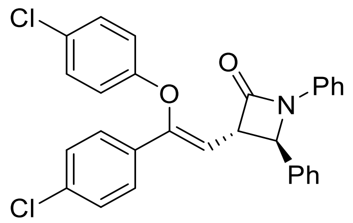

3-((Z)-2-(4-chlorophenoxy)-2-(4-chlorophenyl)vinyl)-1,4-diphenylazetidin-2-one (27):

Purified by column chromatography (SiO_2, pentane/ethyl acetate 9:1), yellow solid, 44.3 mg (91%).

m.p. 88.5 – 89.5 °C

^{1}H NMR (400 MHz, $CDCl_3$): δ = 7.40 – 7.36 (m, 2H), 7.32 – 7.28 (m, 3H), 7.26 (s, 8H), 7.09 – 7.00 (m, 3H), 6.76 – 6.69 (m, 2H), 6.04 (d, *J* = 8.9 Hz, 1H), 4.84 (d, *J* = 2.5 Hz, 1H), 4.11 (dd, *J* = 8.9, 2.5 Hz, 1H) ppm.

^{13}C NMR (400 MHz, $CDCl_3$): δ = 165.5, 155.2, 152.6, 137.6, 137.2, 135.2, 132.4, 129.7 (2C), 129.2 (2C), 129.2 (2C), 129.1 (2C), 128.7, 127.5, 127.3 (2C), 126.1 (2C), 124.2, 117.4 (2C), 117.2 (2C), 110.6, 61.9, 57.9 ppm.

HRMS m/z: $(M+H)^+$ Calcd. for $C_{29}H_{22}Cl_2NO_2$ = 486.1028; **Found:** = 486.1026.

IR: 3854, 3751, 1751, 1540, 1521, 1507, 1497, 1490, 1473, 1457, 1437, 1419 cm^{-1}.

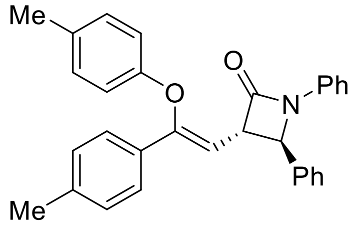

(*Z*)-1,4-diphenyl-3-(2-(p-tolyl)-2-(*p*-tolyloxy)vinyl)azetidin-2-one (28).

Purified by preparative TLC (pentane/ethyl acetate 9:1), yellow oil, 29 mg (65%).

^{1}H NMR (400 MHz, $CDCl_3$): δ = 7.39-7.37 (m, 2H), 7.30-7.21 (m, 9H), 7.10-7.03 (m, 3H), 6.93-6.91 (m, 2H), 6.73-6.71 (m, 2H), 5.98 (d, *J* = 8.7 Hz, 1H), 4.87 (d, *J* = 2.5 Hz, 1H), 4.15 (dd, *J* = 8.7, 2.5 Hz, 1H), 2.30 (s, 3H), 2.20 (s, 3H) ppm.

^{13}C NMR (100 MHz, $CDCl_3$) δ = 166.2, 154.7, 153.9, 138.9, 137.8, 137.5, 131.6, 131.3, 130.0 (2C), 129.4 (2C), 129.14 (2C), 129.05 (2C), 128.5, 126.2 (2C), 126.1(2C), 123.9, 117.2 (2C), 116.0 (2C), 108.6, 62.2, 58.1, 21.4, 20.7 ppm.

HRMS m/z: $(M+H)^+$ Calcd. for $C_{31}H_{28}NO_2$ = 446.2120; **Found:** = 446.2120.

IR: 2922, 2853, 1750, 1653, 1599, 1501, 1456, 1378, 1326, 1286, 1213, 1168, 1117, 1077, 1038, 1015, 983, 908, 813, 751, 691, 499 cm^{-1}.

Table S4. Optimization of the synthesis of β-lactones

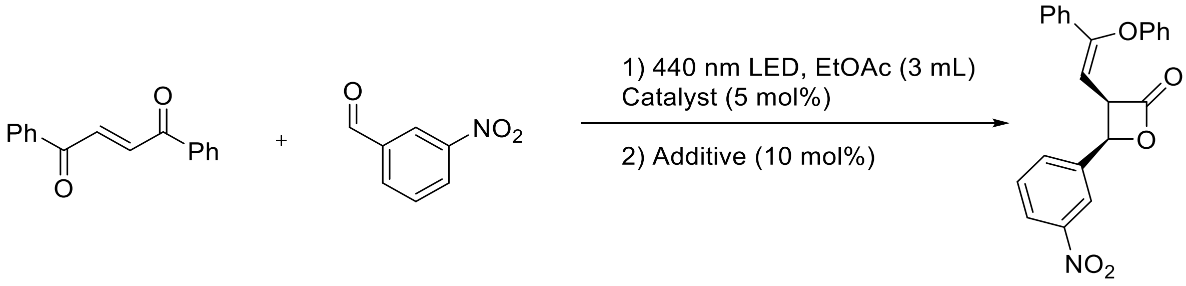

Entry	Catalyst	Additive[a]	Reaction time (h)	Yield (%)[b] **30**
1	-	-	48	0
2	*p*-TsOH	-	48	0
3	BF_3•OEt_3	-	48	0
4	BF_3•OEt_3	Et_3N	24	85

a) Additive added after full conversion of alkene; b) isolated yield.

General procedure for the synthesis of β-lactones

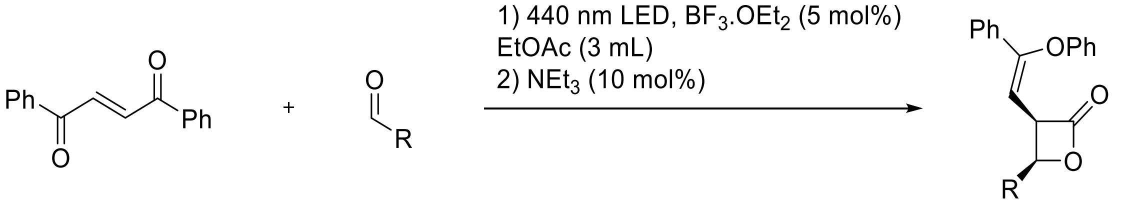

To a 2-5 mL Biotage microwave vial equipped with a magnetic stirrer was added (*E*)-1,4-diphenylbut-2-ene-1,4-dione (0.1 mmol, 1 equiv.) and corresponding aldehyde (0.12 mmol, 1.2 equiv.) under nitrogen atmosphere. Then, a solution of $BF_3 * OEt_2$ (0.05 equiv.) in ethyl acetate (0.03 M) was added under nitrogen. The flask was irradiated with a Kessil 440 nm LED and stirred until full conversion of starting material. A solution of NEt_3 (0.1 equiv.) in EtOAc was then added to the solution and it was stirred for an additional 30 min. The reaction mixture was concentrated *in vacuo* and separated on column chromatography to obtain the products **30-33**.

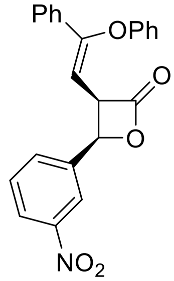

4-(3-nitrophenyl)-3-((*Z*)-2-phenoxy-2-phenylvinyl)oxetan-2-one (30):

Purified by column chromatography (SiO_2, pentane/ethyl acetate 9:1), yellow oil, 32.9 mg (85%).

^{1}H NMR (400 MHz, $CDCl_3$): δ = 8.24 (dd, *J* = 9.9, 1.5 Hz, 2H), 7.71 (d, *J* = 7.7 Hz, 1H), 7.66 – 7.59 (m, 1H), 7.25 – 7.15 (m, 7H), 6.99 (t, *J* = 7.4 Hz, 1H), 6.85 (dt, *J* = 9.2, 1.8 Hz, 2H), 5.82 – 5.74 (m, 1H), 5.31 – 5.23 (m, 2H) ppm.

^{13}C NMR (400 MHz, $CDCl_3$): δ = 168.9, 156.2, 155.0, 148.4, 137.3, 133.3, 131.7, 129.9, 129.8 (2C), 129.5, 128.7 (2C), 126.3 (2C), 123.8, 122.8, 121.2, 116.3 (2C), 103.9, 74.5, 54.1 ppm.

HRMS m/z: $(M+Na)^+$ Calcd. for $C_{23}H_{17}NO_5Na$= 410.1004; **Found:** = 410.1007.

IR: 1824, 1685, 1647, 1592, 1559, 1533, 1489, 1457, 1448, 1346, 1283, 1247, 1185, 1138, 1103, 1077, 1045, 1026, 979 cm^{-1}.

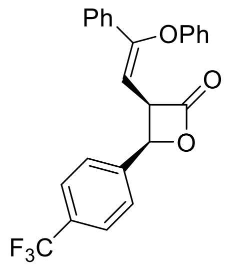

3-((Z)-2-phenoxy-2-phenylvinyl)-4-(4-(trifluoromethyl)phenyl)oxetan-2-one (31):

Purified by column chromatography (SiO_2, pentane/ethyl acetate 9:1), yellow oil, 37.8 mg (92%).

^{1}H NMR (400 MHz, CDCl$_3$): δ = 7.70 (d, *J* = 8.2 Hz, 2H), 7.47 (d, *J* = 8.5 Hz, 2H), 7.26 – 7.16 (m, 7H), 7.04 – 6.95 (m, 1H), 6.89 – 6.81 (m, 2H), 5.75 (d, *J* = 5.0 Hz, 1H), 5.31 – 5.22 (m, 2H) ppm.

^{13}C NMR (400 MHz, CDCl$_3$): δ 169.3, 156.5, 154.7, 139.1, 133.5, 131.3, 129.9 (2C), 129.5 (2C), 128.7 (2C), 126.4 (3C), 126.1 (q, $^1J_{C-F}$ = 265 Hz), 125.8 (q, $^3J_{C-F}$ = 3.5 Hz, 2C), 122.7, 116.3 (2C), 104.5, 74.9, 54.0 ppm.

^{19}F NMR (564 MHz, CDCl$_3$) δ = -62.66 – (-62.67) (m).

HRMS m/z: $(M+H)^+$ Calcd. for $C_{24}H_{18}F_3O_3$ = 411.1208; **Found:** = 411.1208.

IR: 1831, 1753, 1653, 1622, 1592, 1289, 1448, 1420, 1379, 1323, 1211, 1165, 1122, 1067, 1044, 1018, 950, 895, 848, 750, 689, 606 cm^{-1}.

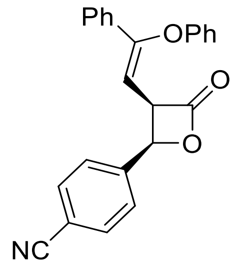

4-((4-oxo-3-((Z)-2-phenoxy-2-phenylvinyl)oxetan-2-yl)benzonitrile (32):

Purified by column chromatography (SiO_2, pentane/ethyl acetate 9:1), yellow oil, 27.6 mg (75%). The isolated product contains trace amount of inseparable unidentified side products.

^{1}H NMR (400 MHz, CDCl$_3$): δ = 7.73 (d, *J* = 8.3 Hz, 2H), 7.47 (d, *J* = 8.3 Hz, 2H), 7.26 – 7.19 (m, 7H), 6.99 (tt, *J* = 7.4, 1.0 Hz, 1H), 6.88 – 6.80 (m, 2H), 5.74 (d, *J* = 5.7 Hz, 1H), 5.30 – 5.19 (m, 2H) ppm.

^{13}C NMR (400 MHz, CDCl$_3$): δ = 169.0, 156.3, 154.9, 140.4, 133.3 (2C), 132.6, 129.9 (2C), 129.6, 128.8 (2C), 126.7 (2C), 126.3 (2C), 122.8, 118.4, 116.3 (2C), 112.8, 104.1, 74.7, 54.2 ppm.

HRMS m/z: $(M+H)^+$ Calcd. for $C_{24}H_{18}NO_3$ = 368.1287; **Found:** = 368.1279.

IR: 3060, 2230, 1820, 1653, 1591, 1488, 1447, 1413, 1333, 1238, 1209, 1165, 1099, 1044, 1024, 949, 895, 859, 752, 733, 691 cm^{-1}.

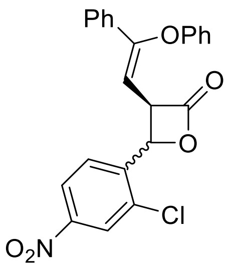

4-(2-chloro-4-nitrophenyl)-3-((Z)-2-phenoxy-2-phenylvinyl)oxetan-2-one (33):

Purified by column chromatography (SiO_2, pentane/ethyl acetate 9:1). The product was obtained as inseparable mixture of two isomers as a yellow oil 37.1 mg (88%).

^{1}H NMR (400 MHz, $CDCl_3$): Isomer **A**: δ 8.54 (dd, *J* = 2.6, 0.8 Hz, 1H), 8.19 (ddd, *J* = 8.7, 2.7, 0.6 Hz, 1H), 7.56 (d, *J* = 8.7 Hz, 1H), 7.21 – 7.11 (m, 6H), 6.93 (ddt, *J* = 7.0, 6.5, 1.1 Hz, 1H), 6.85 – 6.80 (m, 2H), 5.92 (dt, *J* = 6.5, 0.7 Hz, 1H), 5.28 (dd, *J* = 9.9, 6.5 Hz, 1H), 5.15 (d, *J* = 9.9 Hz, 1H) ppm.

Isomer **B**: δ 8.40 (dd, *J* = 2.7, 0.8 Hz, 1H), 8.18 – 8.15 (m, 1H), 7.58 (d, *J* = 8.7 Hz, 2H), 7.52 – 7.47 (m, 2H), 7.33 – 7.28 (m, 3H), 7.21 – 7.13 (m, 2H), 6.90 – 6.87 (m, 2H), 5.98 (d, *J* = 9.6 Hz, 1H), 5.70 (dt, *J* = 4.5, 0.7 Hz, 1H), 4.56 (dd, *J* = 9.6, 4.5 Hz, 1H) ppm.

^{13}C NMR (400 MHz, $CDCl_3$): Isomer **A**: δ 168.0, 155.9, 155.7, 138.5, 135.6, 133.5, 130.6, 129.7 (2C), 129.5, 128.7 (2C), 126.6 (3C), 124.8, 122.9, 122.8, 116.8 (2C), 103.1, 73.2, 54.3 ppm.

Isomer **B**: δ = 167.5, 156.2, 155.3, 147.0, 137.2, 130.8, 129.8 (2C), 129.6, 129.4, 128.8 (2C), 126.6 (2C), 124.8, 122.9, 122.7, 121.8, 116.5 (2C), 106.1, 74.5, 57.6 ppm.

HRMS m/z: $(M+H)^+$ Calcd. for $C_{23}H_{17}ClNO_5$ = 422.0795; **Found:** = 422.0791.

IR: 3027, 1837, 1653, 1592, 1525, 1488, 1447, 1344, 1284, 1208, 1165, 1093, 1044, 1025, 984, 955, 911, 835, 741 690 cm^{-1}.

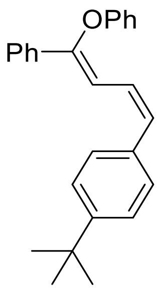

1-(*tert*-butyl)-4-((*1Z,3Z*)-4-phenoxy-4-phenylbuta-1,3-dien-1-yl)benzene (34):

Purified by column chromatography (SiO_2, pentane/ethyl acetate 9:1), yellow oil, 25.5 mg (58%).

^{1}H NMR (400 MHz, $CDCl_3$): δ = 7.60 – 7.52 (m, 2H), 7.37 – 7.27 (m, 6H), 7.23 (dd, *J* = 7.1, 1.5 Hz, 2H), 7.10 (dd, *J* = 15.7, 10.9 Hz, 1H), 7.01 (dt, *J* = 7.8, 1.1 Hz, 2H), 6.95 (tt, *J* = 7.3, 1.1 Hz, 1H), 6.75 – 6.64 (m, 2H), 1.29 (s, 9H) ppm.

^{13}C NMR (400 MHz, $CDCl_3$): δ = 158.0, 151.1, 149.2, 135.1, 134.7, 133.2, 129.8 (2C), 128.7 (2C), 128.4 (2C), 126.4, 125.7 (2C), 125.5 (2C), 122.5, 121.9, 117.8, 115.9 (2C), 34.8, 31.4 (3C) ppm.

HRMS m/z: $(M+H)^+$ Calcd. for $C_{26}H_{27}O$ = 355.2062; **Found:** = 355.2056.

IR: 2961, 1591, 1488, 1446, 1346, 1319, 1271, 1212, 1164, 1107, 1024, 1013, 968, 876, 751, 690 cm^{-1}.

Further transformations

Acid catalyzed hydrolysis of compound 6

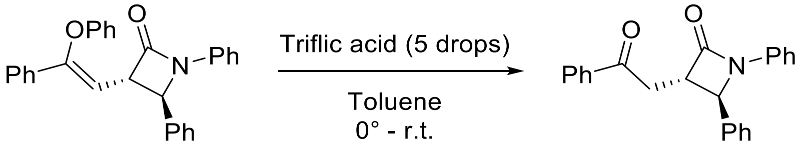

To the small 5 mL flask with magnetic stirrer was added **6** (41 mg, 0.1 mmol) in 1 mL of toluene. After that, 5 drops of trifluoromethanesulfonic acid were added at 0° and the mixture was stirred until full conversion of starting material (monitored by TLC). After that the reaction crude was quenched with aqueous solution of $NaHCO_3$ and extracted to EtOAc (3x) and the organic layers were concentrated *in vacuo* and separated using column chromatography (SiO_2, 5:1 hexane/EtOAc) to obtain the ketone **35** (29.0 mg, 85%)**.**

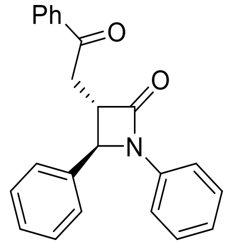

3-(2-oxo-2-phenylethyl)-1,4-diphenylazetidin-2-one (35):

Purified by column chromatography (SiO_2, hexane/ethyl acetate 5:1), yellow oil, 29.0 mg (85%).

^{1}H NMR (400 MHz, $CDCl_3$): δ = 8.01 – 7.96 (m, 2H), 7.60 (dd, *J* = 10.5, 4.3 Hz, 1H), 7.49 (t, *J* = 7.6 Hz, 2H), 7.46 – 7.40 (m, 3H), 7.38 – 7.27 (m, 4H), 7.24 (d, *J* = 8.5 Hz, 2H), 7.08 – 7.02 (m, 1H), 4.81 (d, *J* = 2.2 Hz, 1H), 3.79 (dd, *J* = 17.6, 3.3 Hz, 1H), 3.59 – 3.53 (m, 1H), 3.47 (dd, *J* = 17.6, 10.5 Hz, 1H) ppm.

^{13}C NMR (400 MHz, $CDCl_3$): δ = 196.8, 167.0, 137.8, 137.7, 133.8, 129.2 (2C), 129.1 (2C), 129.0 (2C), 128.5, 128.3 (2C), 126.4 (2C), 124.1, 117.2 (2C), 115.4, 61.7, 55.6, 37.8 ppm.

HRMS m/z: $(M+H)^+$ Calcd. For $C_{23}H_{19}NO_2$ = 342.1494; **Found:** = 342.1496.

IR: 1734, 1684, 1653, 1636, 1617, 1593, 1577, 1559, 1540, 1521, 1506, 1491, 1447, 1387, 1354, 1326, 1308, 1226, 1145, 1002, 795, 764, 749, 724, 701, 691, 668 cm^{-1}.

Reduction of compound 35

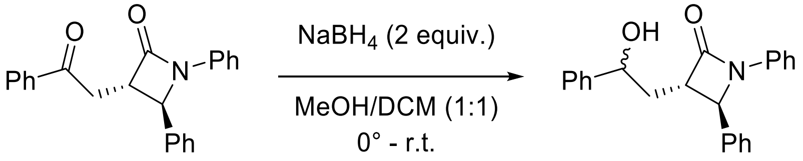

To a 5 mL flask with magnetic stirrer was added **35** (31 mg, 0.1 mmol, 1 equiv.) in 2 mL of MeOH/DCM (1:1). After that, $NaBH_4$ (7.6 mg, 0.2 mmol, 2 equiv.) was added at 0° and the mixture was stirred until full conversion of starting material. After that the reaction crude was quenched with water and extracted to EtOAc (3x) and the organic layers washed with brine, concentrated in vacuo and separated using column chromatography (SiO_2, 5:1 hexane/EtOAc) to obtain compound **36** (26.8 mg, 78%).

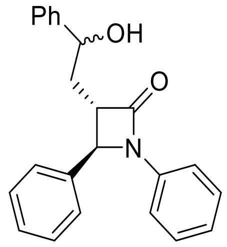

3-(2-hydroxy-2-phenylethyl)-1,4-diphenylazetidin-2-one (36):

Purified by column chromatography (SiO_2, hexane/ethyl acetate 5:1). The product was obtained as a colorless oil as an inseparable mixture of diastereomers (1:1), 26.8 mg (78%).

^{1}H NMR (400 MHz, $CDCl_3$): δ = 7.40 – 7.34 (m, 3H), 7.34 – 7.30 (m, 4H), 7.30 – 7.26 (m, 4H), 7.26 – 7.18 (m, 3H), 7.05 (qt, *J* = 7.1, 1.6 Hz, 1H), 5.02 – 4.89 (m, 1H), 4.77 (dd, *J* = 24.0, 2.4 Hz, 1H), 3.24 (dtd, *J* = 9.6, 7.7, 7.3, 2.5 Hz, 1H), 2.43 – 2.22 (m, 2H) ppm.

^{13}C NMR (400 MHz, $CDCl_3$): δ = 173.7 (2C), 145.0, 143.8, 137.8, 137.7, 137.53, 137.46, 129.3 (2C), 129.23 (2C), 129.18 (4C), 128.8, 128.7 (2C), 128.64 (2C), 128.60, 127.84, 127.80, 126.3 (2C), 126.1 (2C), 125.9 (2C), 125.6 (2C), 124.3, 124.1, 117.22 (2C), 117.18 (2C), 73.2, 73.0, 61.7, 61.2, 58.3, 57.8, 38.9, 38.2 ppm.

HRMS m/z: $(M+H)^+$ Calcd. For $C_{23}H_{22}NO_2$ = 344.1651; **Found:** = 344.1631.

IR: 2919, 2850, 1717, 1653, 1598, 1500, 1455, 1386, 1354, 1142, 1057, 1026, 898, 797, 750, 608, 508 cm^{-1}.

Hydrogenolysis of compound 35

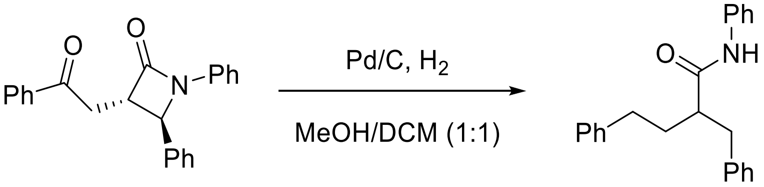

To a 5 mL flask with magnetic stirrer was added **35** (31 mg, 0.1 mmol, 1 equiv.) in 2 mL of MeOH/DCM (1:1). After that Pd/C (1.05 mg, 10 mol. % of 10% Pd/C) was added under hydrogen atmosphere and the mixture was stirred until full conversion of starting material. After that the reaction crude was filtered through Celite ® and concentrated in vacuo and separated using column chromatography (SiO_2, 5:1 hexane/EtOAc) to obtain compound **37** (29.3 mg, 89%).

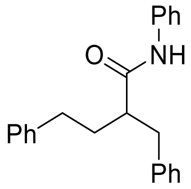

2-benzyl-*N*,4-diphenylbutanamide (37):

Purified by column chromatography (SiO_2, pentane/ethyl acetate 9:1), yellow oil, 29.3 mg (89%).

^{1}H NMR (400 MHz, $CDCl_3$): δ = 7.34 – 7.27 (m, 6H), 7.26 – 7.20 (m, 3H), 7.17 (tt, *J* = 5.9, 1.4 Hz, 4H), 7.09 (tt, *J* = 7.1, 1.5 Hz, 1H), 6.70 (s, 1H), 3.02 (dd, *J* = 13.4, 9.4 Hz, 1H), 2.88 – 2.75 (m, 2H), 2.63 (dt, *J* = 13.9, 8.0 Hz, 1H), 2.39 (tt, *J* = 9.6, 5.0 Hz, 1H), 2.27 – 2.14 (m, 1H), 1.91 (dddd, *J* = 13.5, 8.9, 7.5, 4.6 Hz, 1H) ppm.

^{13}C NMR (400 MHz, $CDCl_3$): δ = 173.1, 141.5, 139.6, 137., 129.0, 129.0, 128.7, 128.6, 128.6, 126.6, 126.2, 124.5, 120.2, 50.4, 39.6, 34.0, 33.6 ppm.

HRMS m/z: $(M+H)^+$ Calcd. for $C_{23}H_{23}O$ = 330.1858; **Found:** = 330.1858.

IR: 3027, 2920, 2854, 1734, 1654, 1597, 1531, 1454, 1443, 1384, 1307, 1252, 1208, 1160, 1076, 1029, 942, 910, 809 740, 691 cm^{-1}.

NMR spectra of products

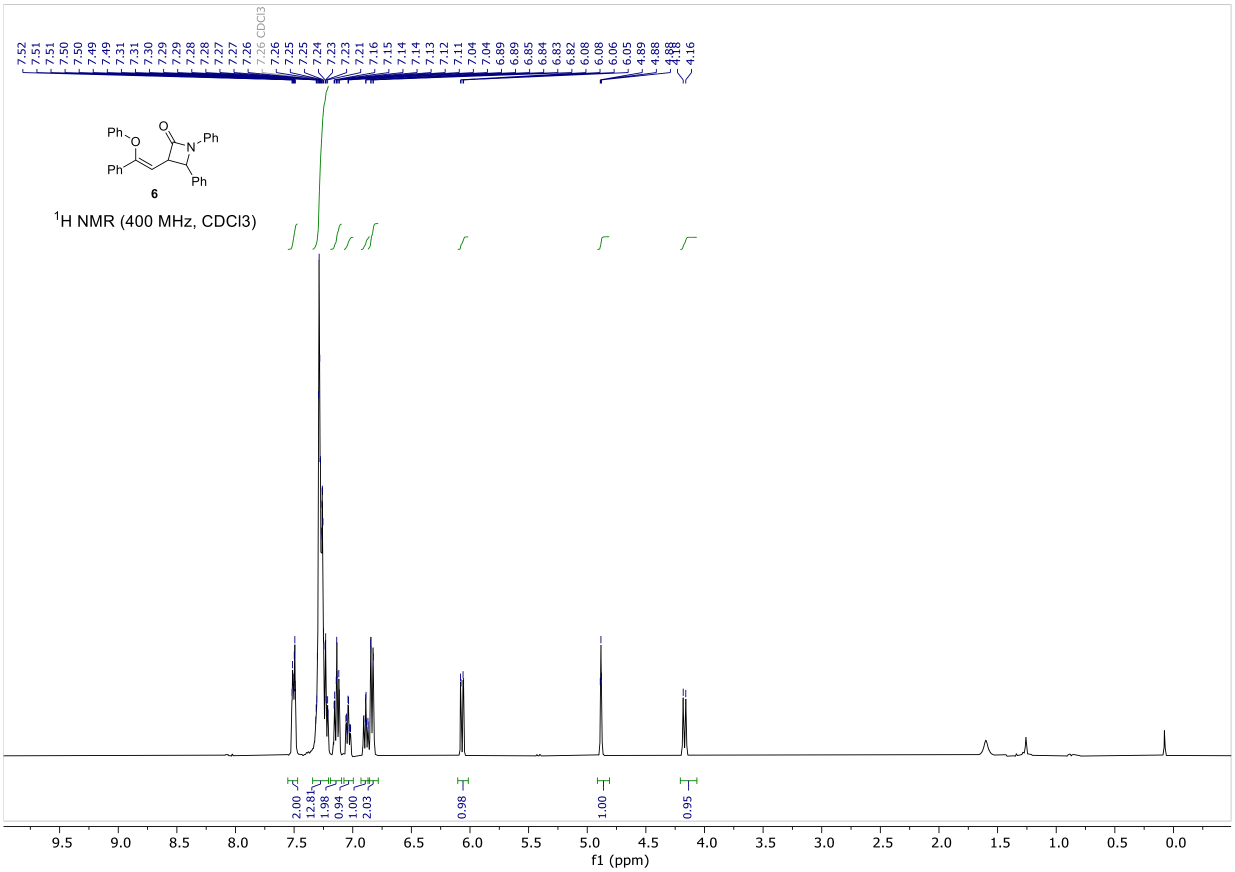

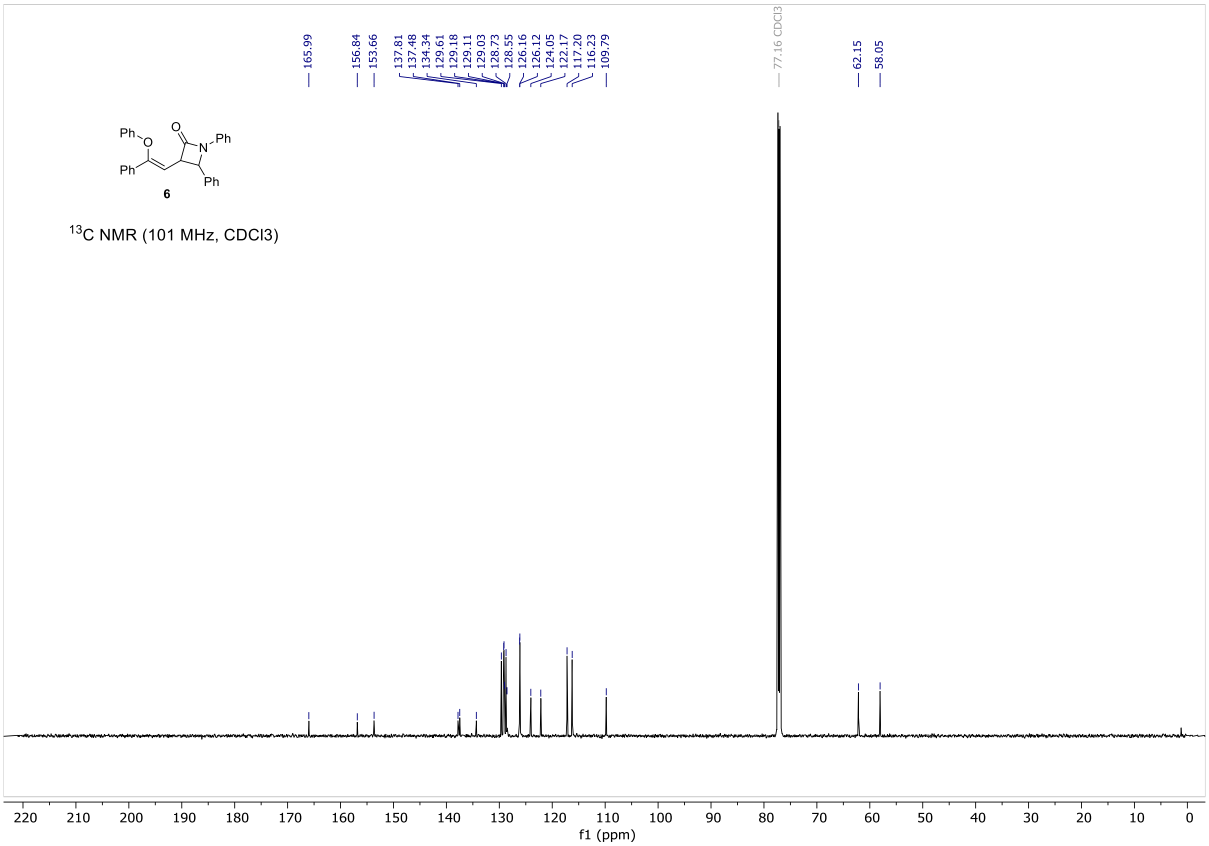

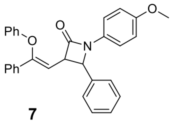

7

^{1}H NMR (400 MHz, CDCl3)

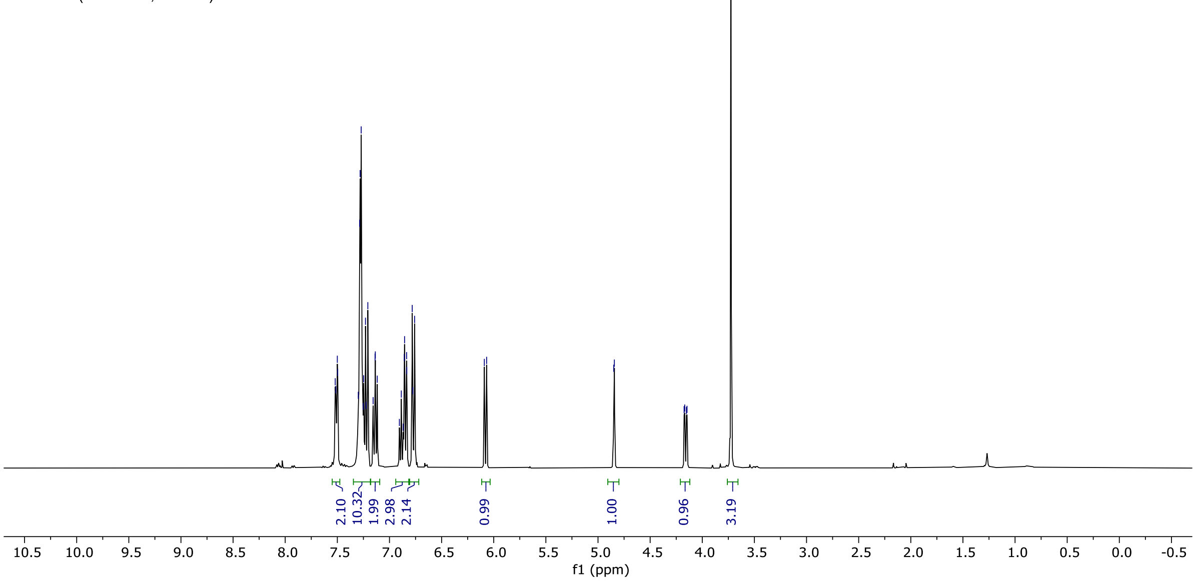

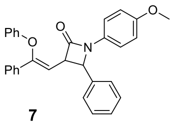

7

^{13}C NMR (101 MHz, CDCl3)

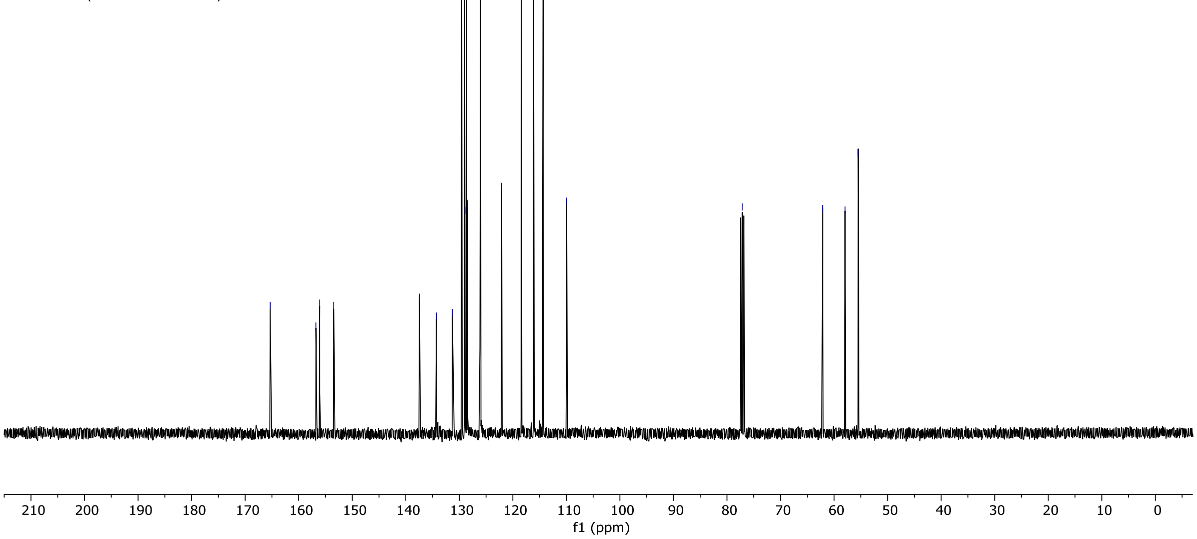

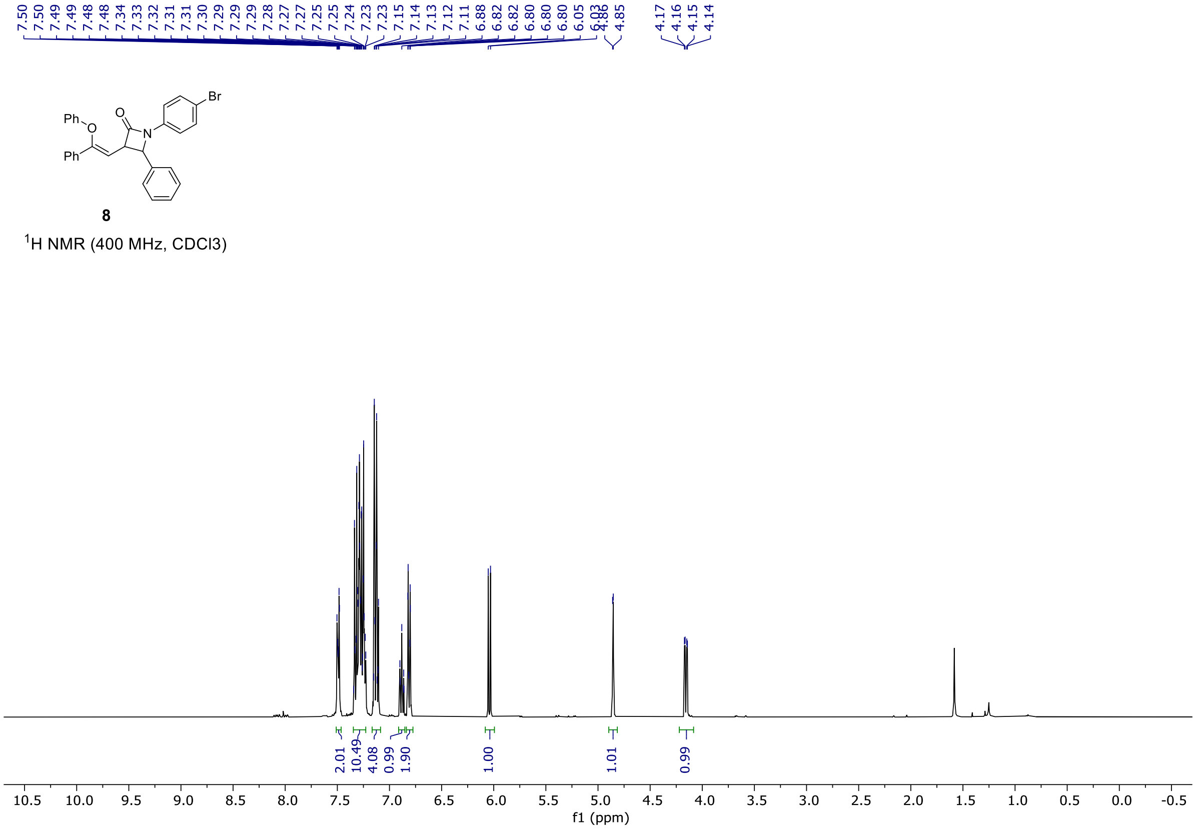

7.50 7.50 7.49 7.49 7.48 7.48 7.34 7.33 7.32 7.31 7.31 7.30 7.29 7.29 7.29 7.28 7.27 7.27 7.25 7.25 7.24 7.23 7.23 7.23 7.15 7.14 7.13 7.12 7.11 6.88 6.82 6.82 6.80 6.80 6.80 6.05 6.03 4.86 4.85 4.17 4.16 4.15 4.14
Ph
O
O
N
Br
Ph
8
^{1}H NMR (400 MHz, CDCl3)
2.01 10.49 4.08 0.99 1.90 1.00 1.01 0.99
f1 (ppm)

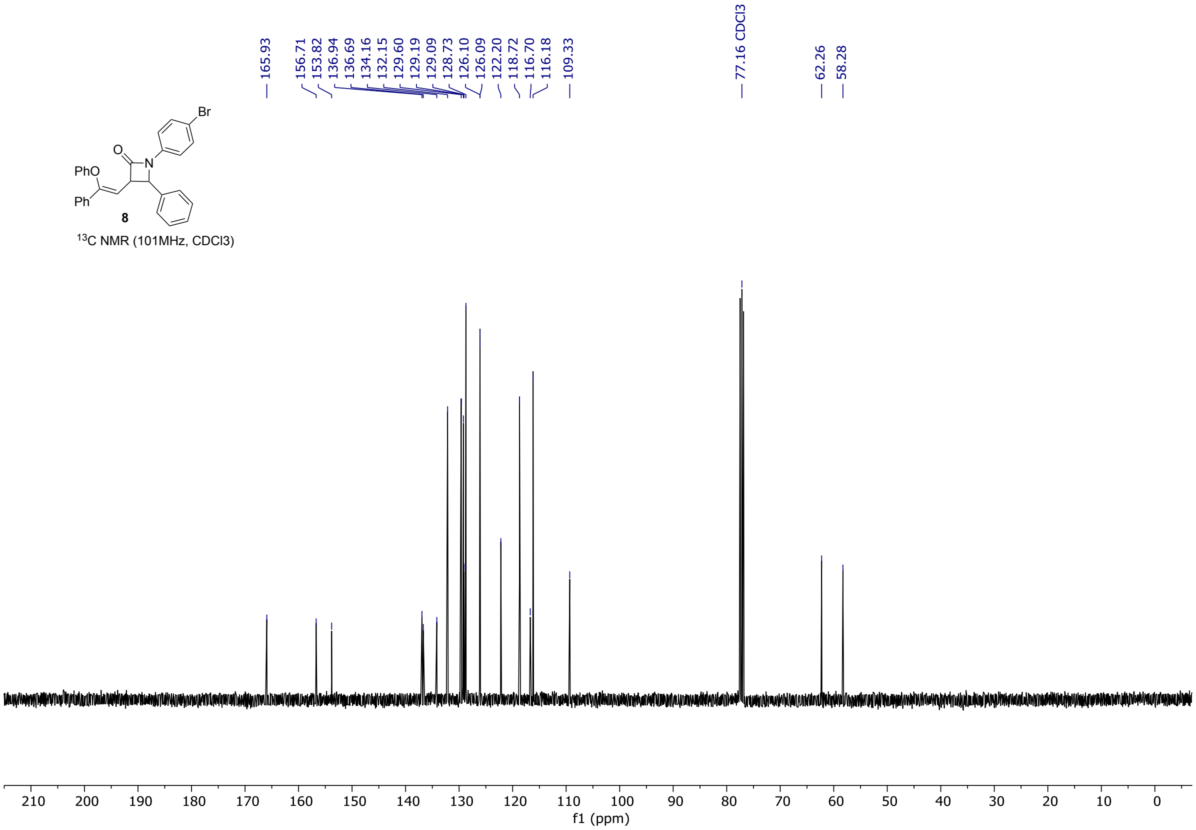

165.93 156.71 153.82 136.94 136.69 134.16 132.15 129.60 129.19 129.09 128.73 126.10 126.09 122.20 118.72 116.70 116.18 109.33
77.16 CDCl3
62.26 58.28
O
N
Br
PhO
Ph
8
^{13}C NMR (101MHz, CDCl3)
f1 (ppm)

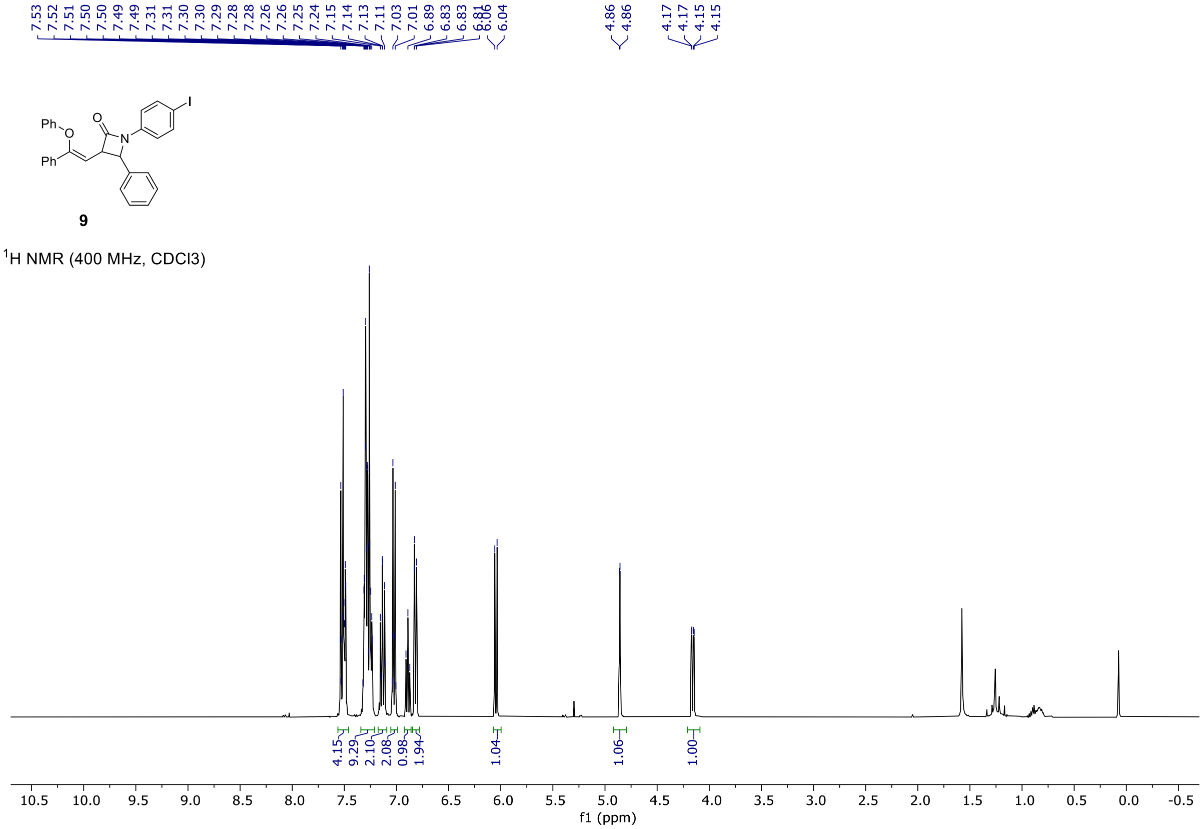
7.53
7.52
7.51
7.50
7.50
7.49
7.49
7.31
7.31
7.30
7.30
7.29
7.28
7.28
7.26
7.26
7.25
7.24
7.15
7.14
7.13
7.11
7.03
7.01
6.89
6.83
6.83
6.81
6.06
6.04
4.86
4.86
4.17
4.17
4.15
4.15
Ph
O
O
N
I
Ph
9
1H NMR (400 MHz, CDCl3)
4.15
9.29
2.10
2.08
0.98
1.94
1.04
1.06
1.00
10.5 10.0 9.5 9.0 8.5 8.0 7.5 7.0 6.5 6.0 5.5 5.0 4.5 4.0 3.5 3.0 2.5 2.0 1.5 1.0 0.5 0.0 -0.5
f1 (ppm)

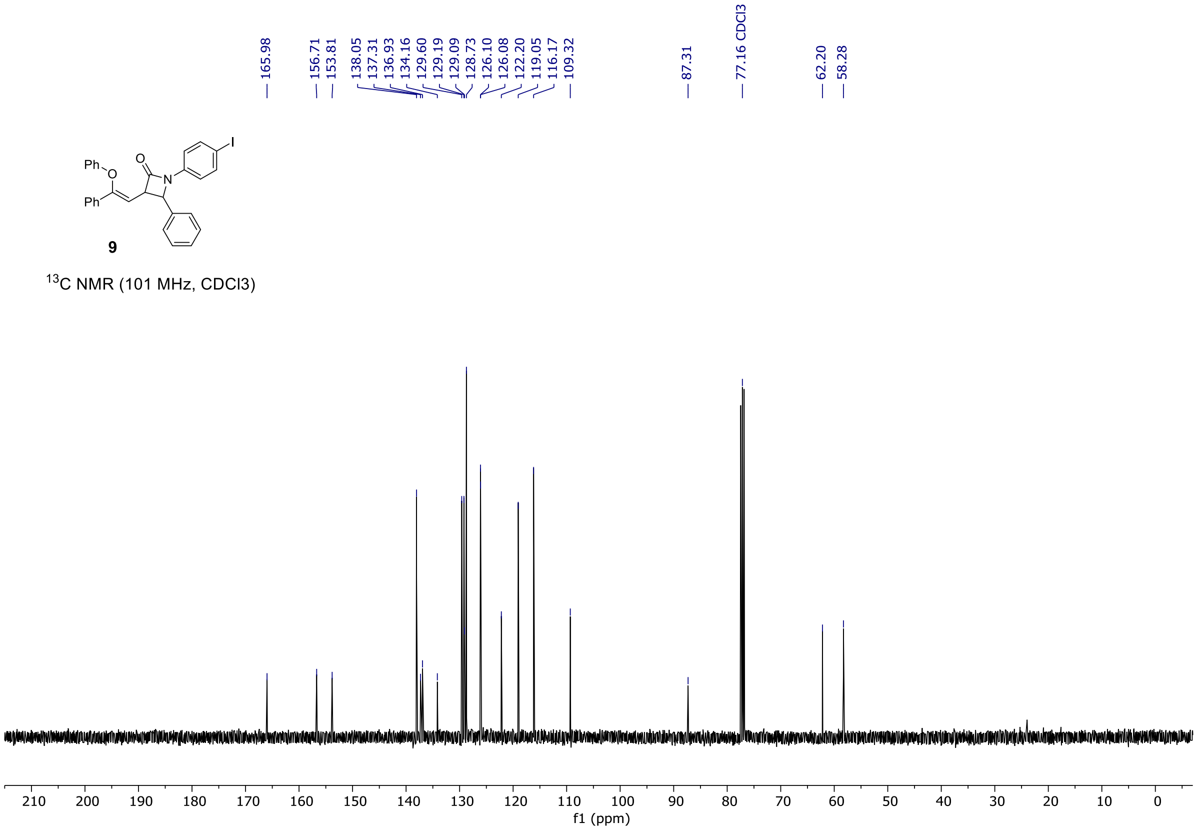
165.98
156.71
153.81
138.05
137.31
136.93
134.16
129.60
129.19
129.09
128.73
126.10
126.08
122.20
119.05
116.17
109.32
87.31
77.16 CDCl3
62.20
58.28
Ph
O
O
N
I
Ph
9
13C NMR (101 MHz, CDCl3)
210 200 190 180 170 160 150 140 130 120 110 100 90 80 70 60 50 40 30 20 10 0
f1 (ppm)

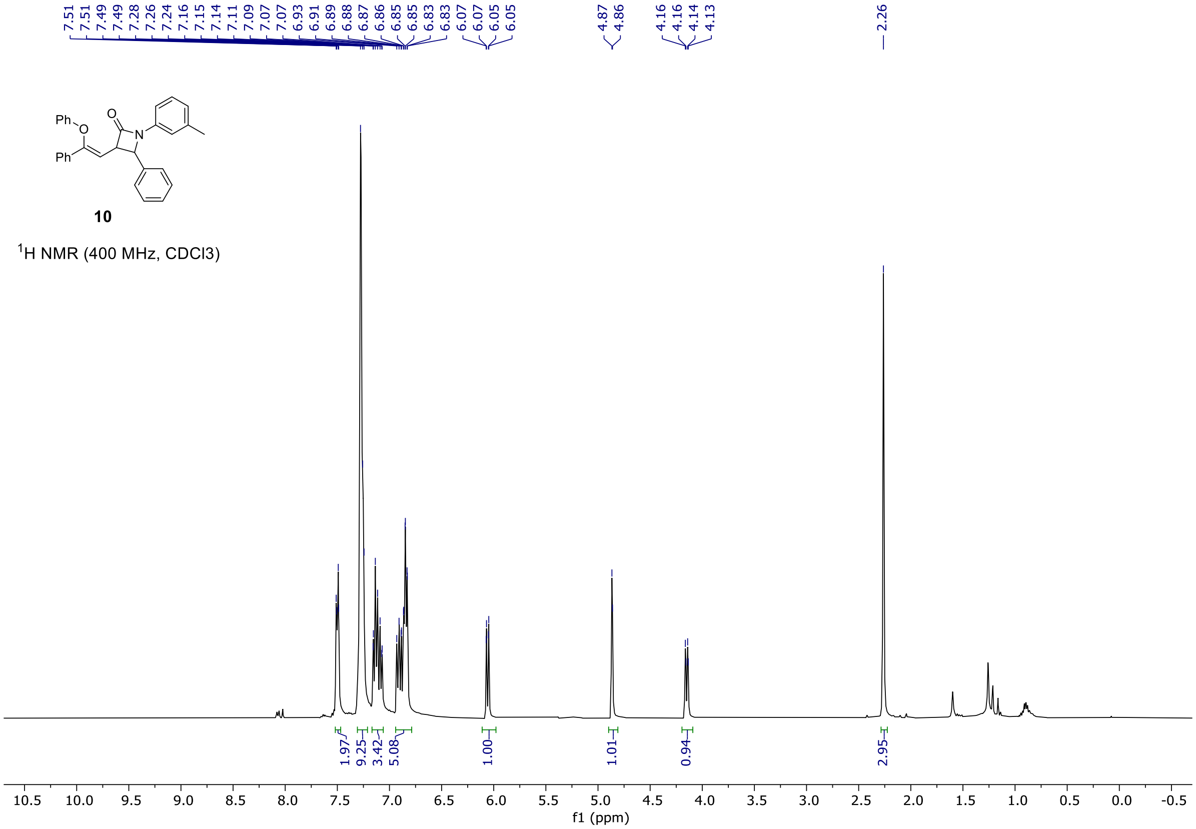
7.51 7.51 7.49 7.49 7.28 7.26 7.24 7.16 7.15 7.14 7.11 7.09 7.07 7.07 6.93 6.91 6.89 6.88 6.87 6.86 6.85 6.85 6.83 6.83 6.07 6.07 6.05 6.05
4.87 4.86
4.16 4.16 4.14 4.13
2.26
Ph
O
O
N
Ph
10
1H NMR (400 MHz, CDCl3)
1.97 9.25 3.42 5.08
1.00
1.01
0.94
2.95
f1 (ppm)

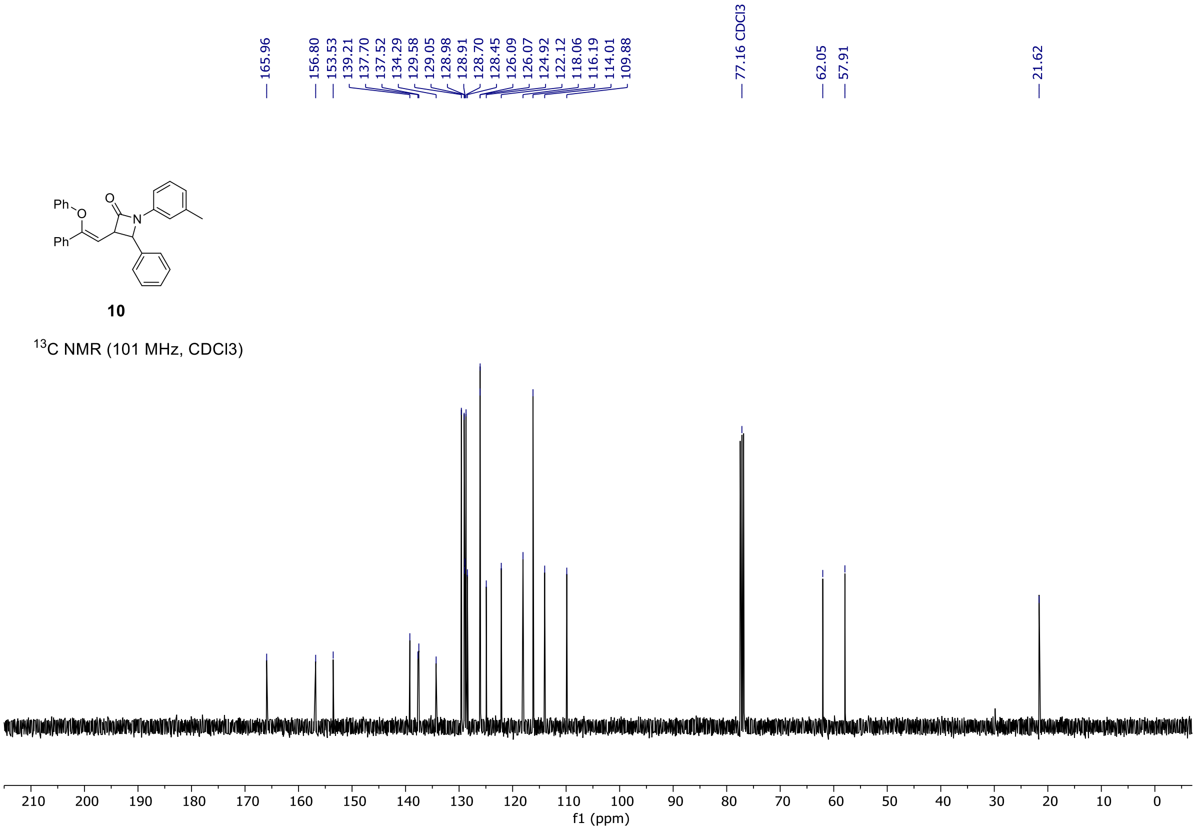
165.96
156.80 153.53 139.21 137.70 137.52 134.29 129.58 129.05 128.98 128.91 128.70 128.45 126.09 126.07 124.92 122.12 118.06 116.19 114.01 109.88
77.16 CDCl3
62.05 57.91
21.62
Ph
O
O
N
Ph
10
13C NMR (101 MHz, CDCl3)
f1 (ppm)

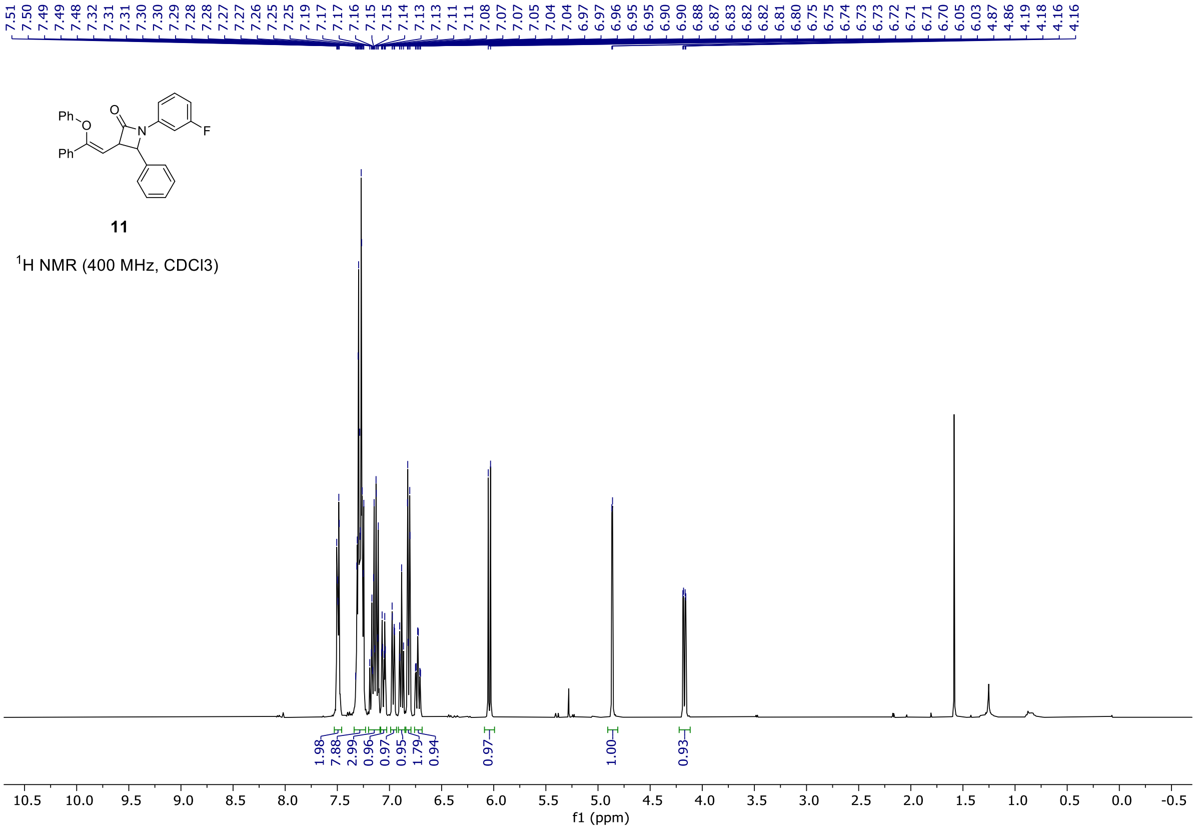
7.51 7.50 7.49 7.48 7.32 7.31 7.30 7.30 7.29 7.28 7.28 7.27 7.27 7.26 7.25 7.25 7.19 7.17 7.17 7.16 7.15 7.15 7.14 7.13 7.13 7.11 7.11 7.08 7.07 7.07 7.05 7.04 7.04 6.97 6.97 6.96 6.95 6.95 6.90 6.90 6.88 6.87 6.83 6.82 6.82 6.81 6.80 6.75 6.74 6.73 6.73 6.72 6.71 6.71 6.70 6.05 6.03 4.87 4.86 4.19 4.18 4.16 4.16
Ph
O
Ph
O
N
F
11
^{1}H NMR (400 MHz, CDCl3)
1.98 7.88 2.99 0.96 0.97 0.95 1.79 0.94 0.97 1.00 0.93
f1 (ppm)

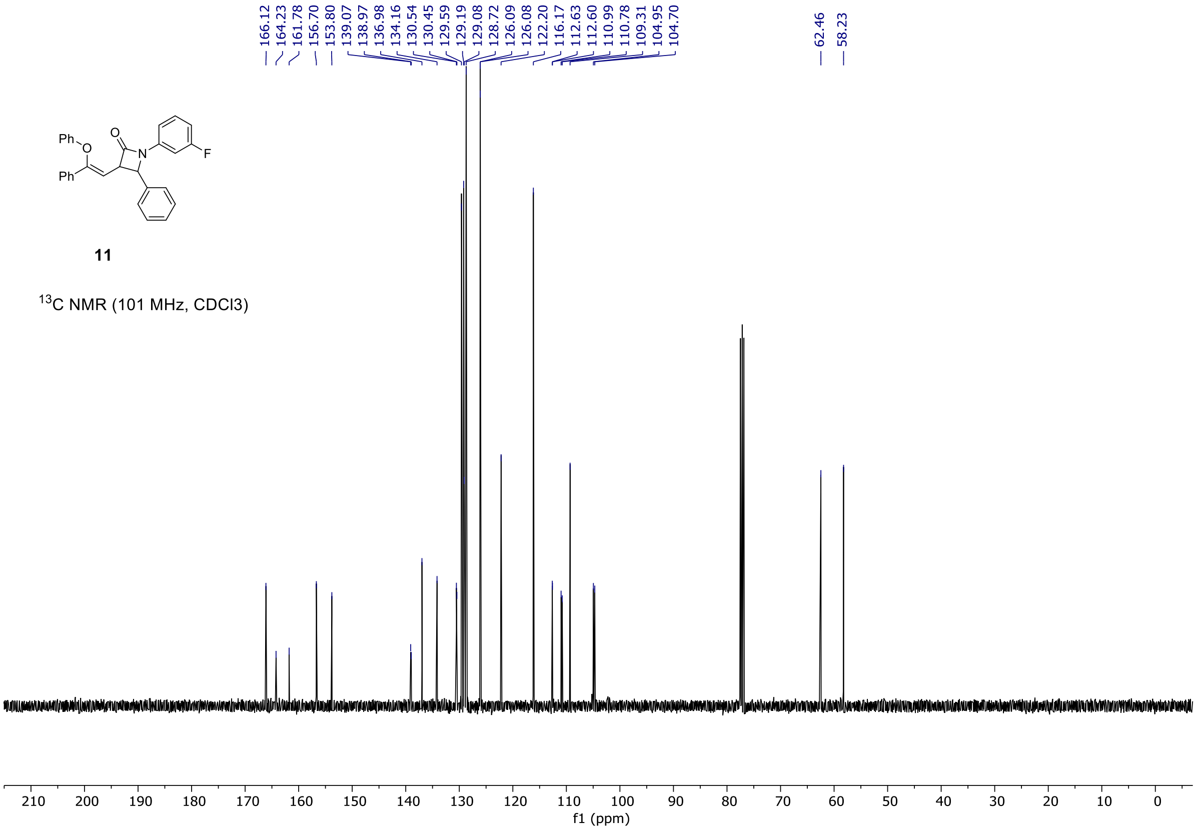
166.12 164.23 161.78 156.70 153.80 139.07 138.97 136.98 134.16 130.54 130.45 129.59 129.19 129.08 128.72 126.09 126.08 122.20 116.17 112.63 112.60 110.99 110.78 109.31 104.95 104.70
62.46 58.23
Ph
O
Ph
O
N
F
11
^{13}C NMR (101 MHz, CDCl3)
f1 (ppm)

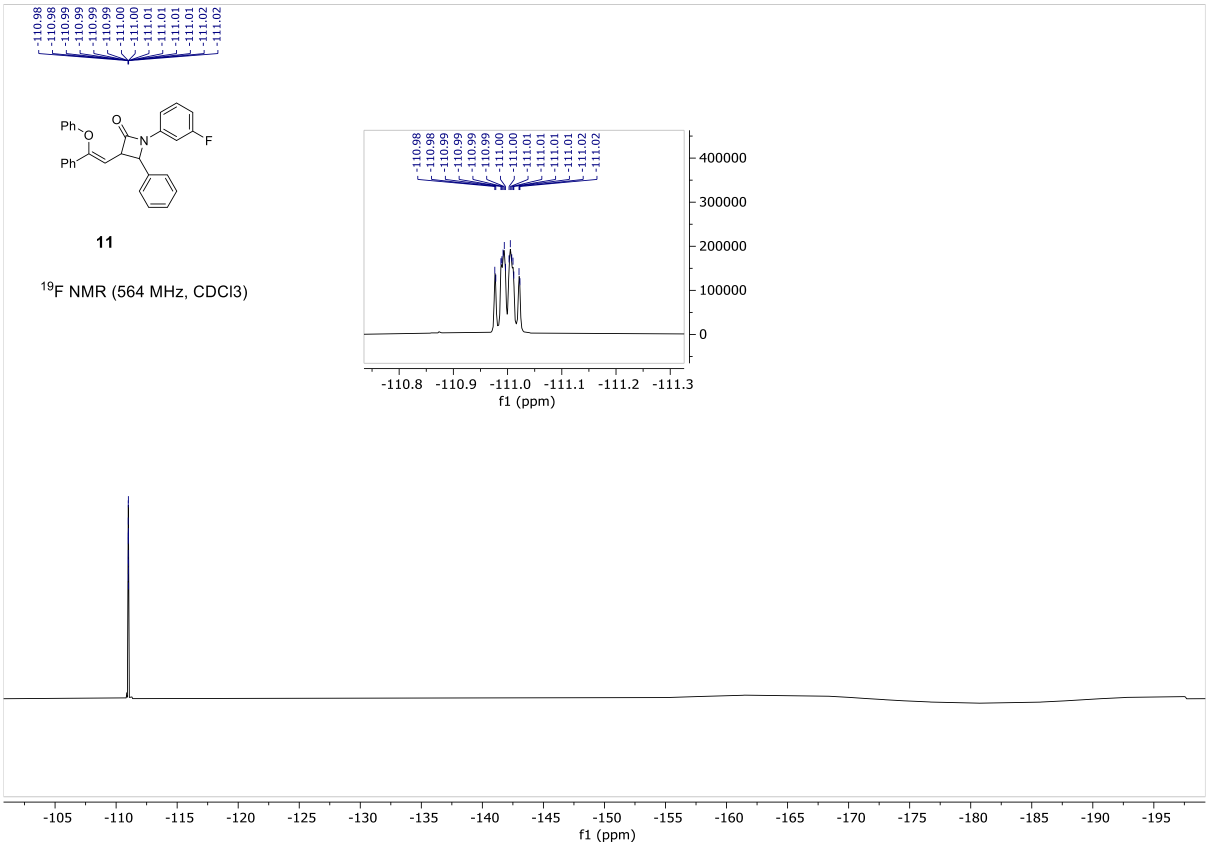

-110.98
-110.98
-110.99
-110.99
-110.99
-110.99
-111.00
-111.00
-111.01
-111.01
-111.01
-111.01
-111.02
-111.02
Ph
O
Ph
O
N
F
11
^{19}F NMR (564 MHz, CDCl3)
400000
300000
200000
100000
0
-110.8
-110.9
-111.0
-111.1
-111.2
-111.3
f1 (ppm)
-105
-110
-115
-120
-125
-130
-135
-140
-145
-150
-155
-160
-165
-170
-175
-180
-185
-190
-195
f1 (ppm)

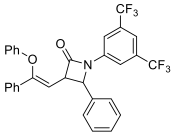

12

^{1}H NMR (400 MHz, CDCl3)

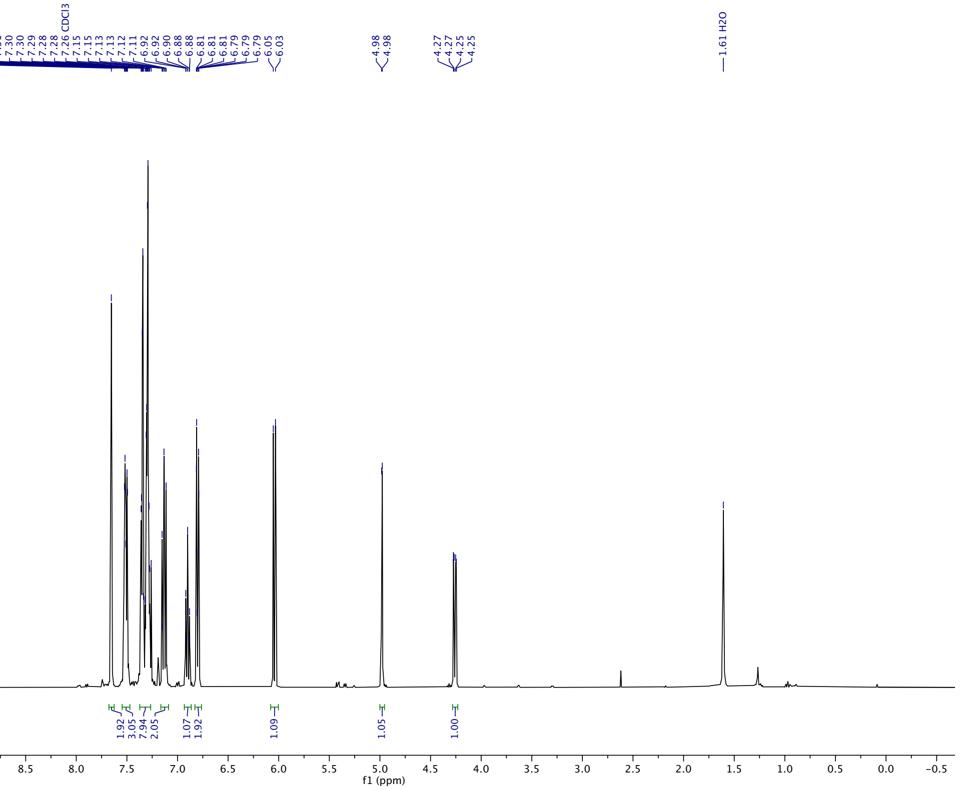

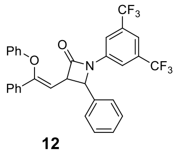

12

^{13}C NMR (101 MHz, CDCl3)

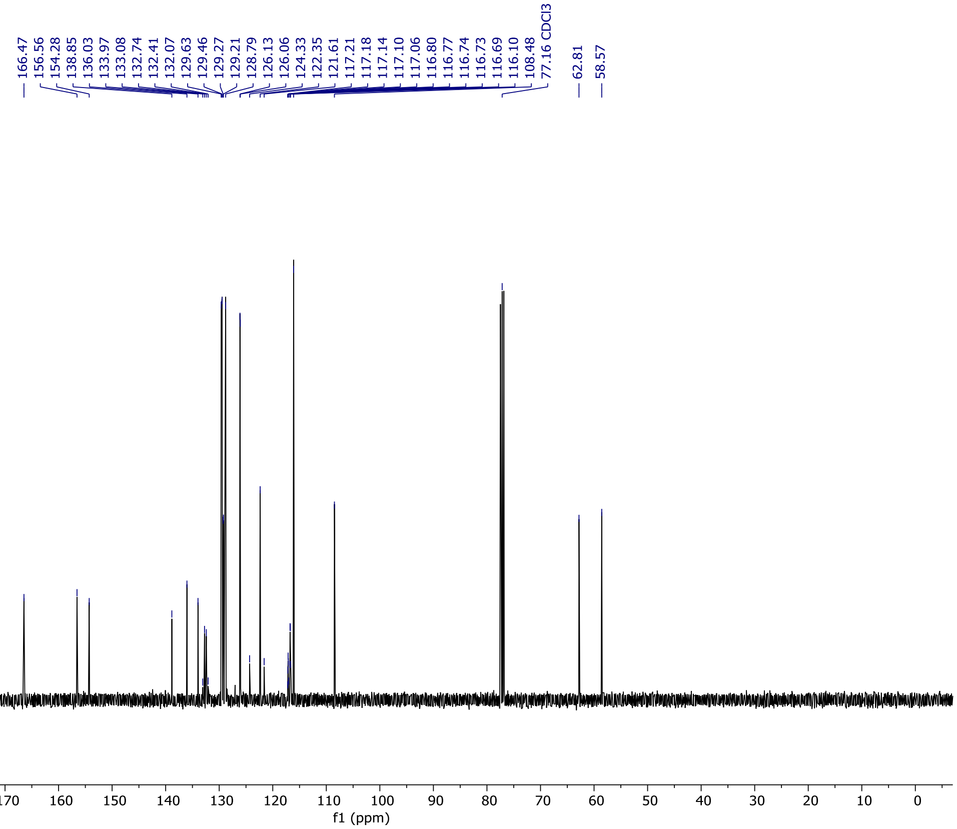

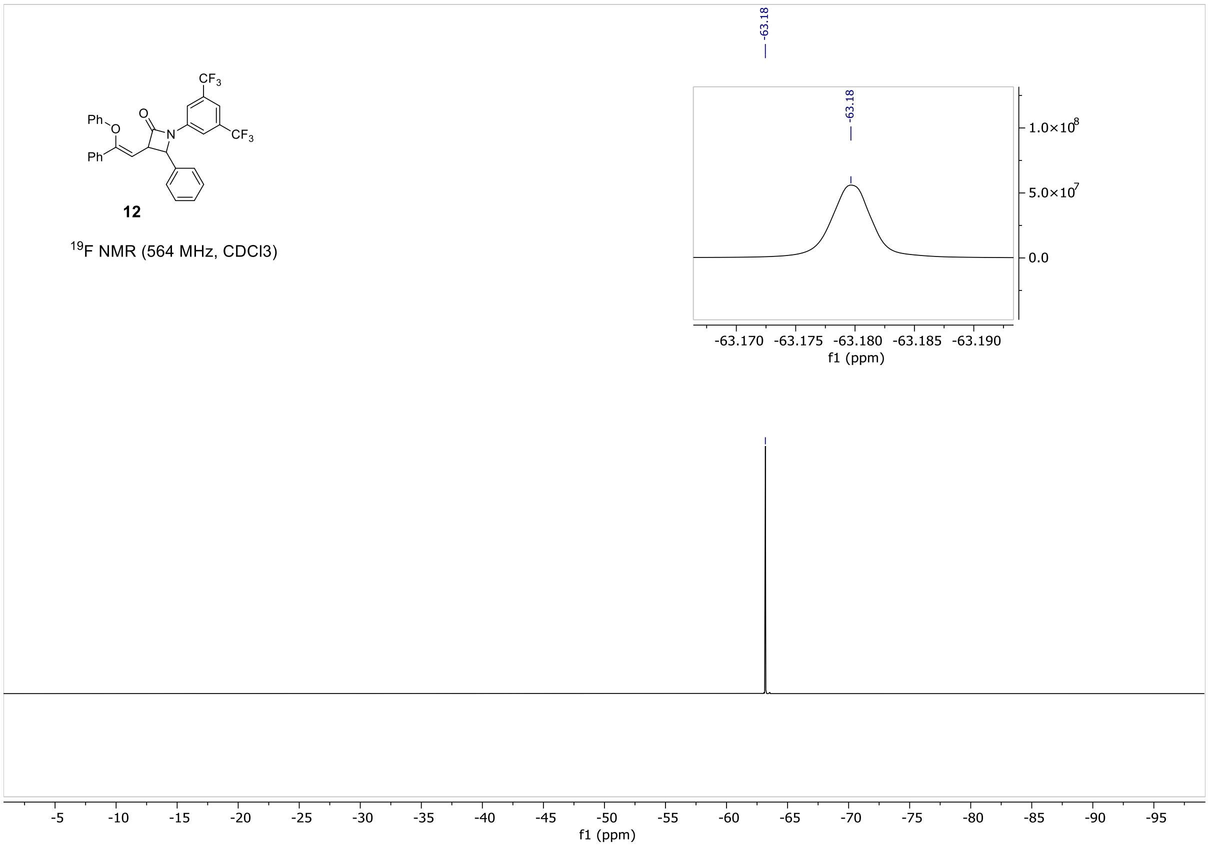
12
^{19}F NMR (564 MHz, CDCl3)
-63.18
f1 (ppm)

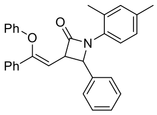

13

^{1}H NMR (400 MHz, CDCl3)

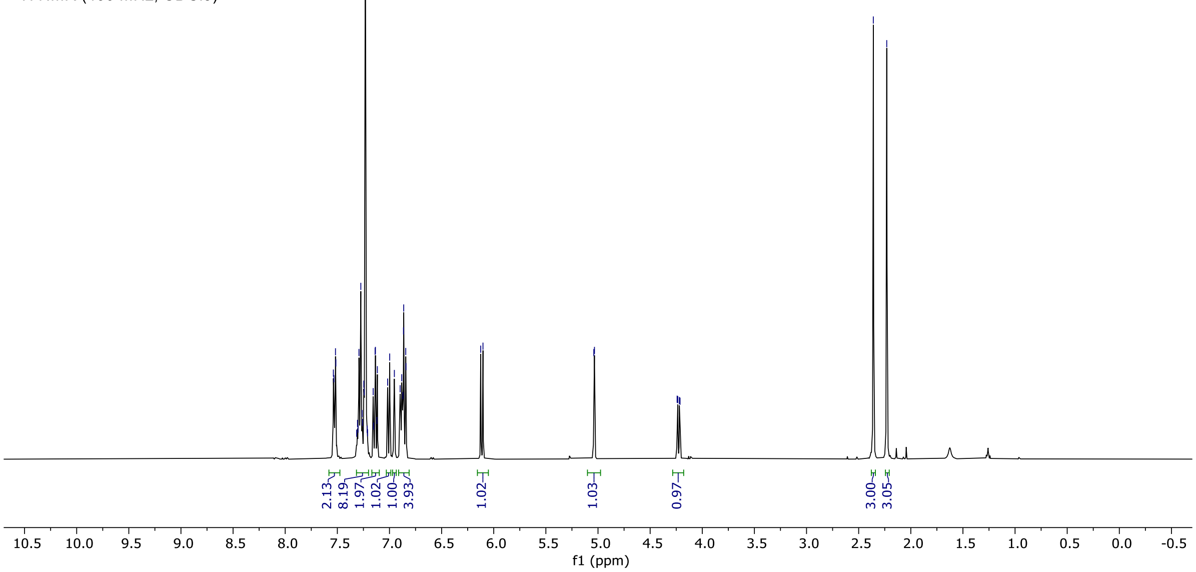

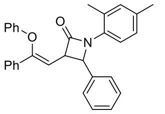

13

^{13}C NMR (101 MHz, CDCl3)

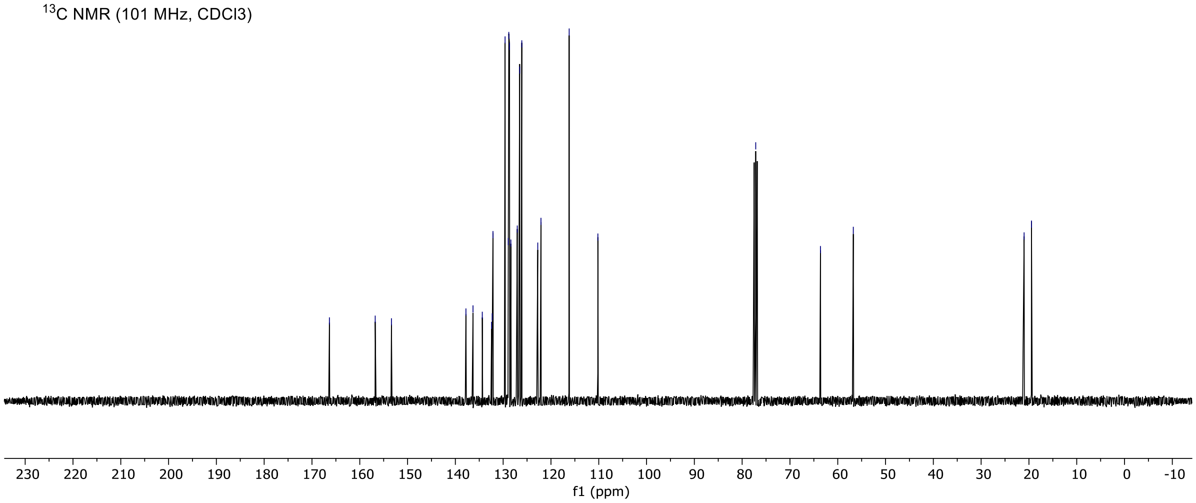

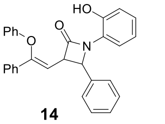

14

^{1}H NMR (400 MHz, CDCl3)

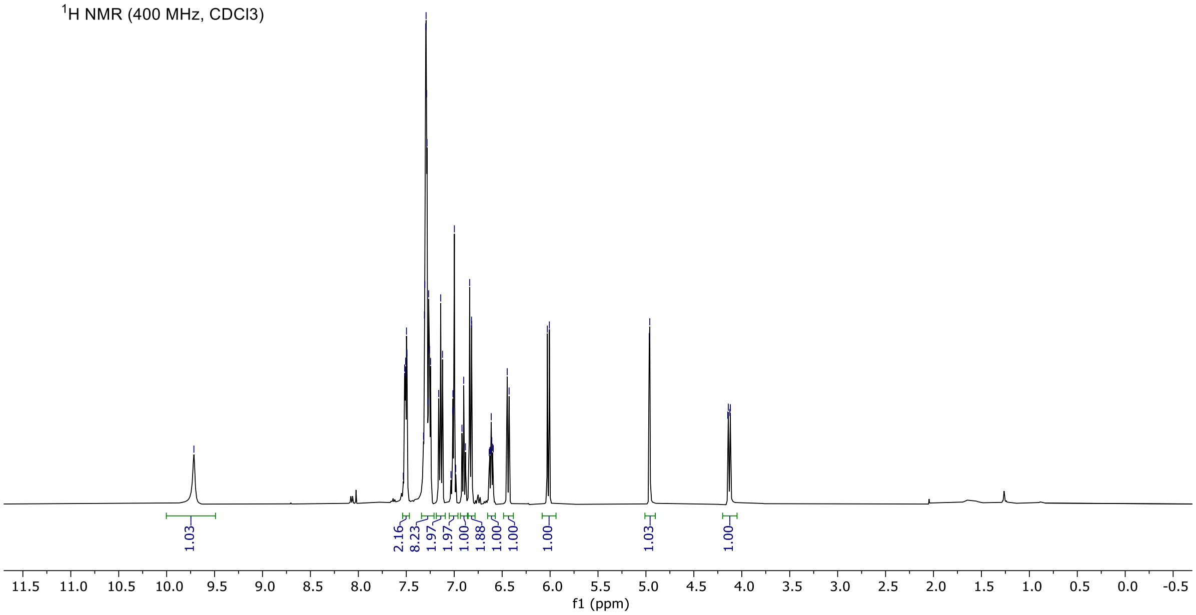

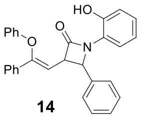

14

^{13}C NMR (101 MHz, CDCl3)

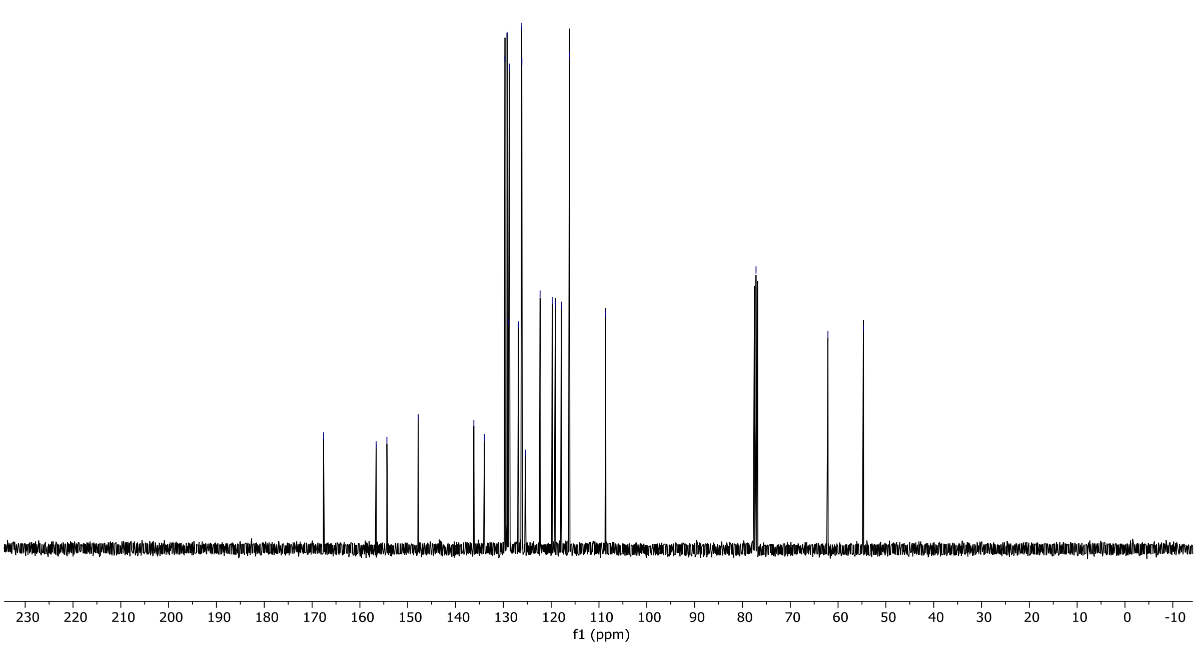

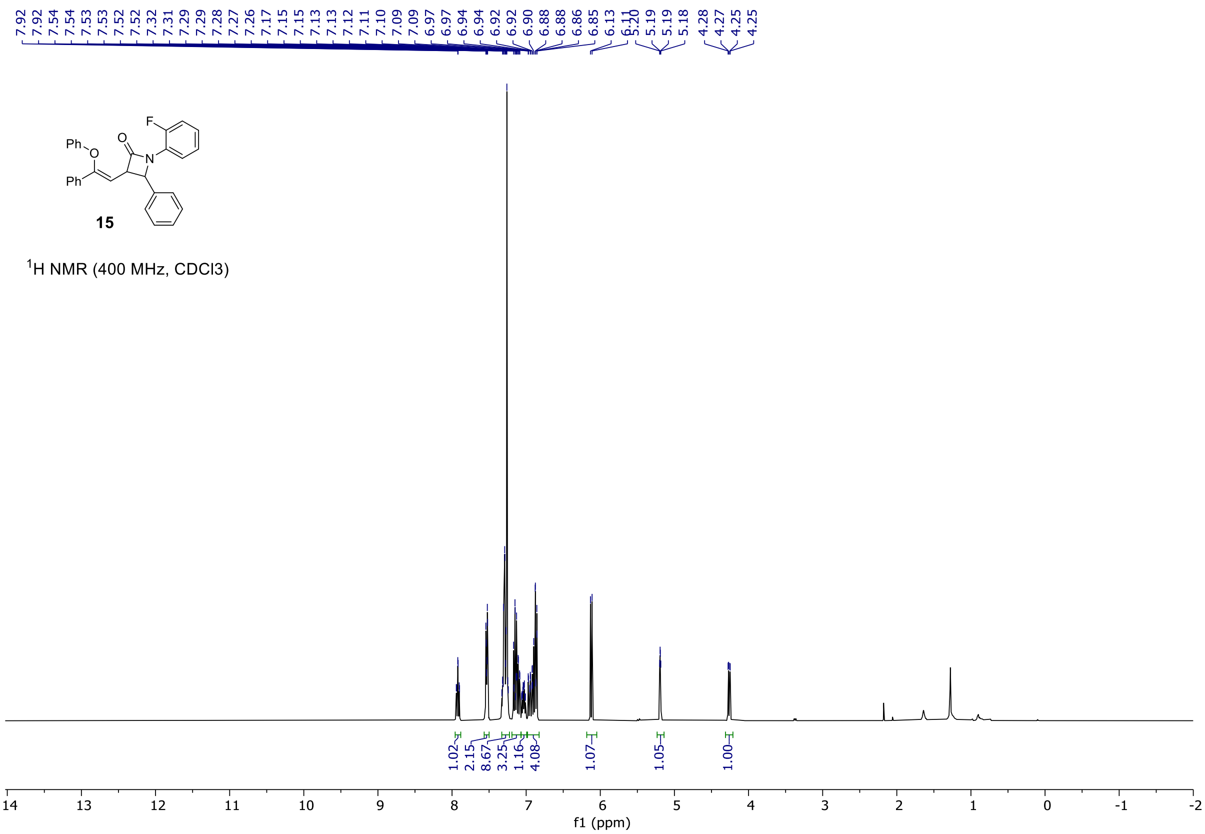
7.92 7.92 7.54 7.54 7.53 7.53 7.52 7.52 7.32 7.31 7.29 7.29 7.28 7.27 7.26 7.17 7.15 7.15 7.13 7.13 7.12 7.11 7.10 7.09 7.09 6.97 6.97 6.94 6.94 6.92 6.92 6.90 6.88 6.88 6.86 6.85 6.13 6.11 5.20 5.19 5.19 5.18 4.28 4.27 4.25 4.25
Ph
O
Ph
O
N
F
15
^{1}H NMR (400 MHz, CDCl3)
1.02 2.15 8.67 3.25 1.16 4.08 1.07 1.05 1.00
14 13 12 11 10 9 8 7 6 5 4 3 2 1 0 -1 -2
f1 (ppm)

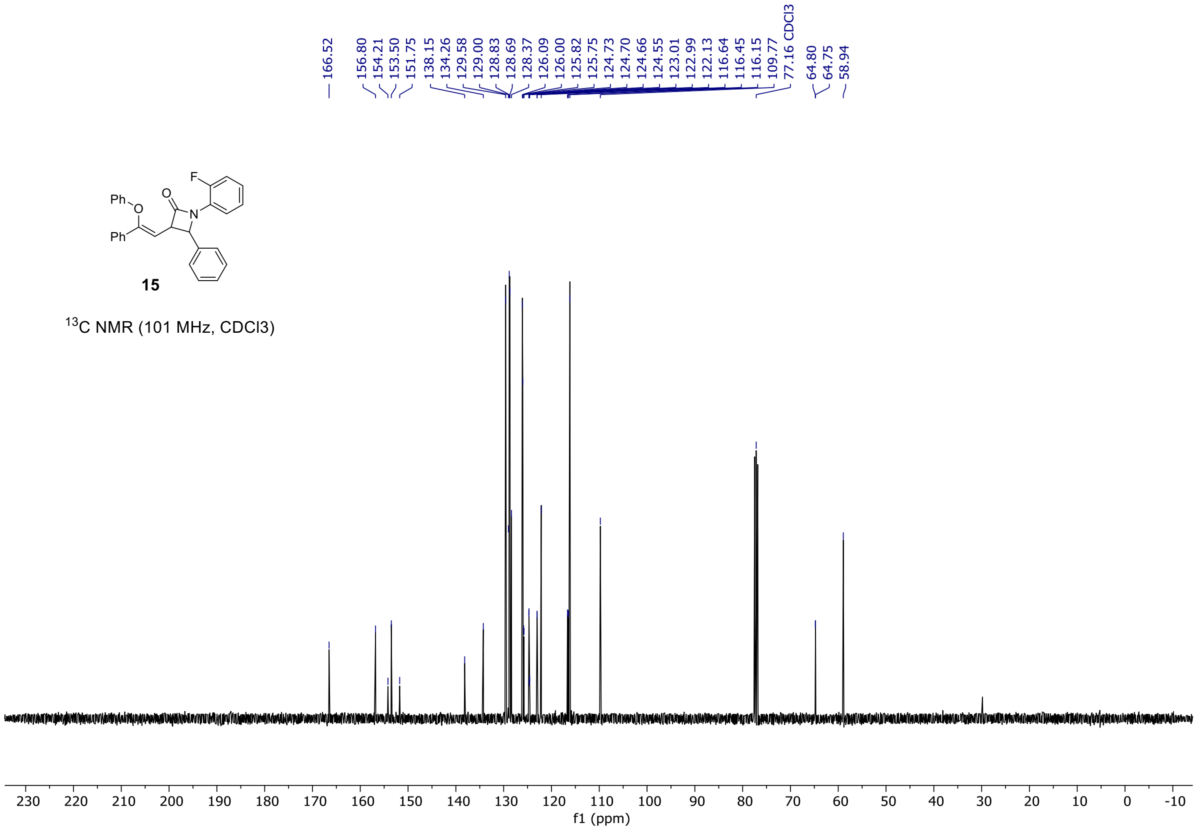
166.52 156.80 154.21 153.50 151.75 138.15 134.26 129.58 129.00 128.83 128.69 128.37 126.09 126.00 125.82 125.75 124.73 124.70 124.66 124.55 123.01 122.99 122.13 116.64 116.45 116.15 109.77 77.16 CDCl3 64.80 64.75 58.94
Ph
O
Ph
O
N
F
15
^{13}C NMR (101 MHz, CDCl3)
230 220 210 200 190 180 170 160 150 140 130 120 110 100 90 80 70 60 50 40 30 20 10 0 -10
f1 (ppm)

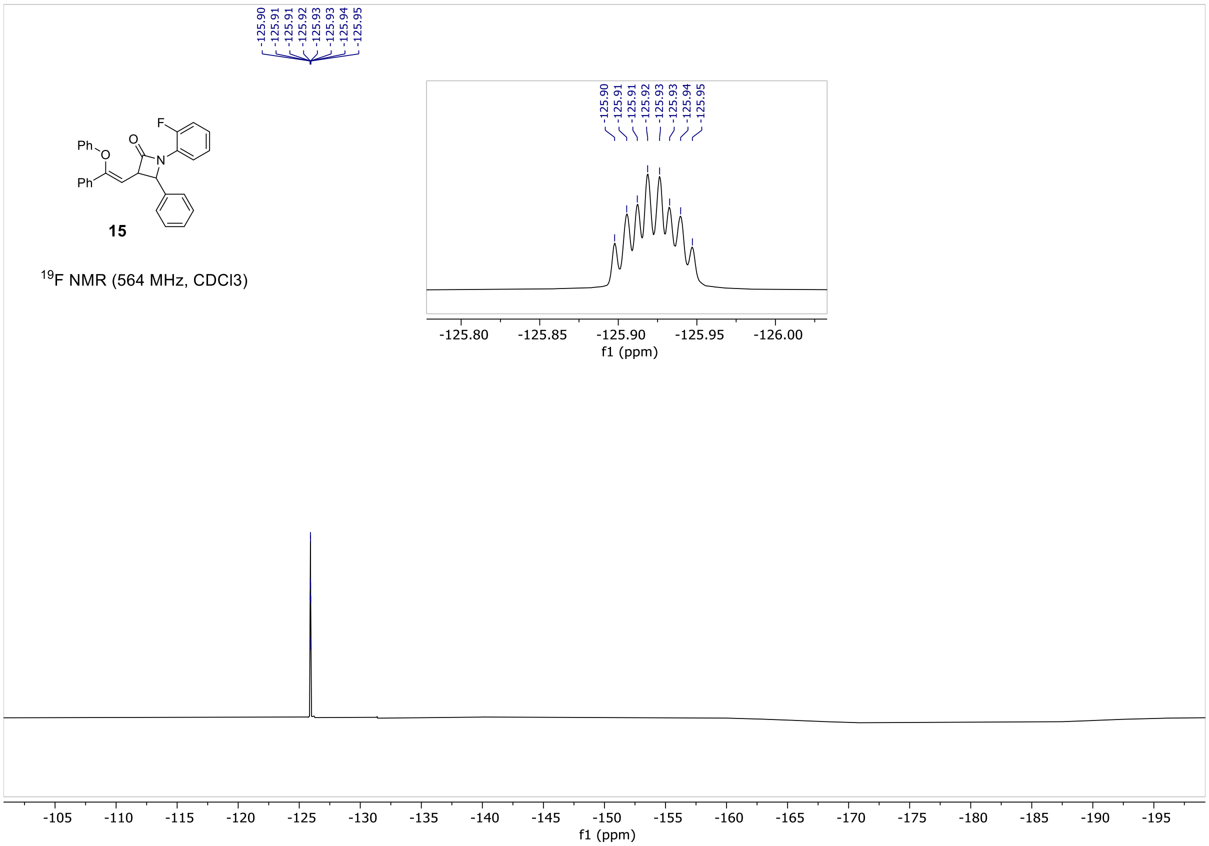

Ph
O
Ph
O
N
F
15
19F NMR (564 MHz, CDCl3)
-125.90
-125.91
-125.91
-125.92
-125.93
-125.93
-125.94
-125.95
-125.80
-125.85
-125.90
-125.95
-126.00
f1 (ppm)
-105
-110
-115
-120
-125
-130
-135
-140
-145
-150
-155
-160
-165
-170
-175
-180
-185
-190
-195
f1 (ppm)

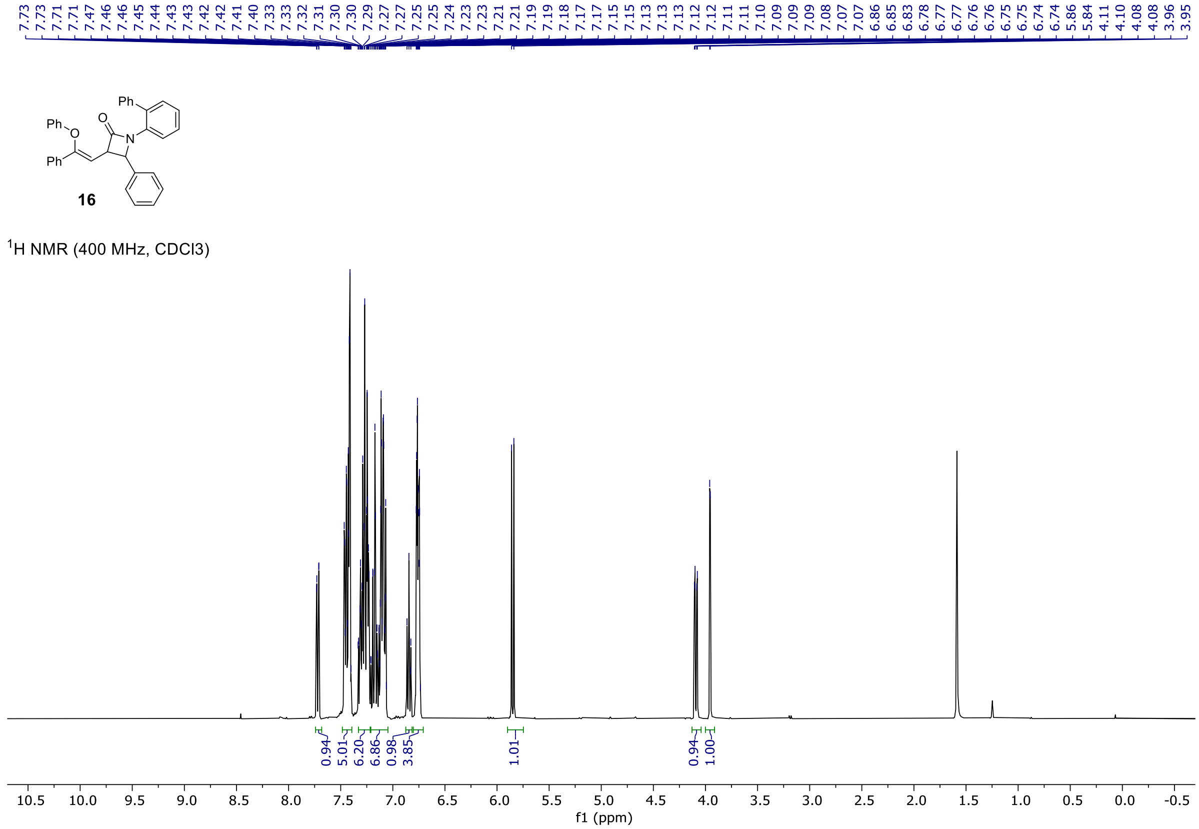
7.73 7.73 7.71 7.47 7.46 7.45 7.44 7.43 7.43 7.42 7.42 7.41 7.40 7.33 7.33 7.32 7.31 7.30 7.30 7.29 7.27 7.27 7.25 7.25 7.24 7.23 7.23 7.21 7.21 7.19 7.19 7.18 7.17 7.17 7.15 7.15 7.13 7.13 7.13 7.12 7.12 7.11 7.11 7.10 7.09 7.09 7.08 7.07 7.07 6.86 6.85 6.83 6.78 6.77 6.77 6.76 6.76 6.75 6.75 6.74 6.74 5.86 5.84 4.11 4.10 4.08 4.08 3.96 3.95
Ph
Ph
O
O
N
Ph
16
1H NMR (400 MHz, CDCl3)
0.94
5.01
6.20
6.86
0.98
3.85
1.01
0.94
1.00
10.5 10.0 9.5 9.0 8.5 8.0 7.5 7.0 6.5 6.0 5.5 5.0 4.5 4.0 3.5 3.0 2.5 2.0 1.5 1.0 0.5 0.0 -0.5
f1 (ppm)

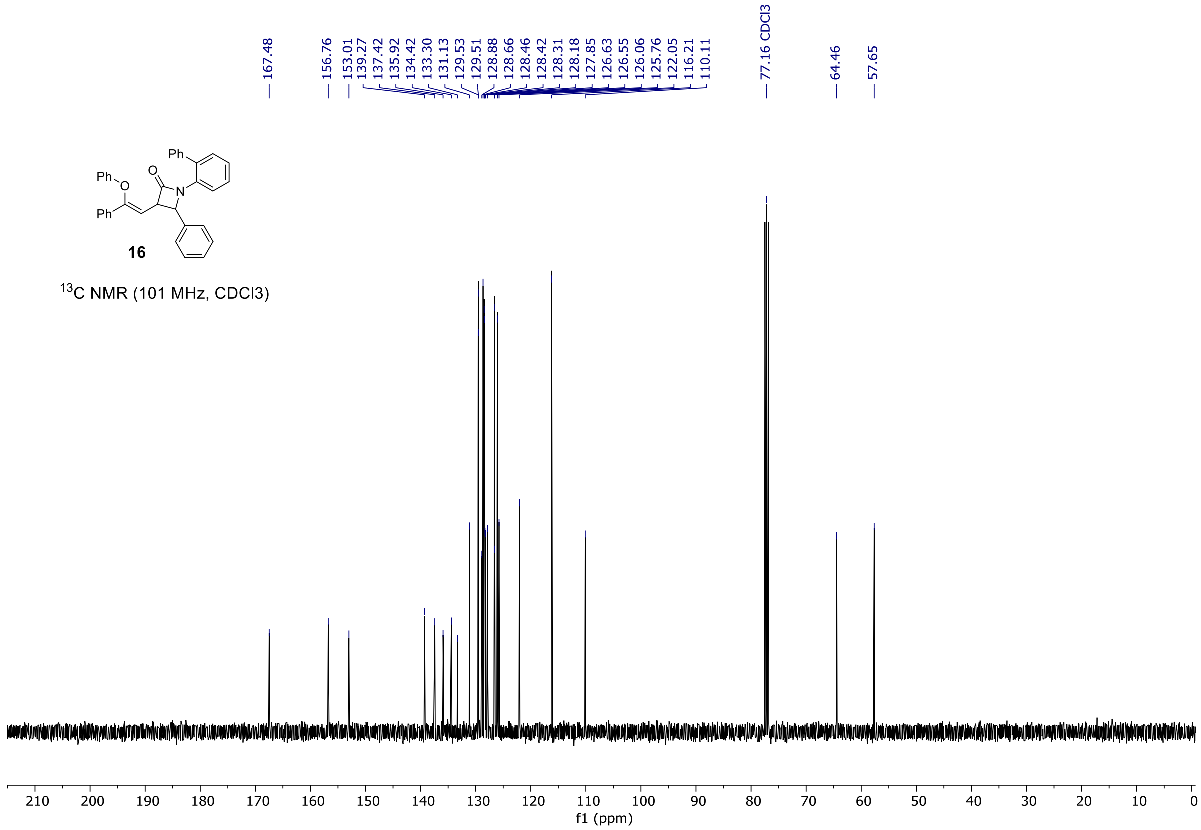
167.48
156.76 153.01 139.27 137.42 135.92 134.42 133.30 131.13 129.53 129.51 128.88 128.66 128.46 128.42 128.31 128.18 127.85 126.63 126.55 126.06 125.76 122.05 116.21 110.11
77.16 CDCl3
64.46
57.65
Ph
Ph
O
O
N
Ph
16
13C NMR (101 MHz, CDCl3)
210 200 190 180 170 160 150 140 130 120 110 100 90 80 70 60 50 40 30 20 10 0
f1 (ppm)

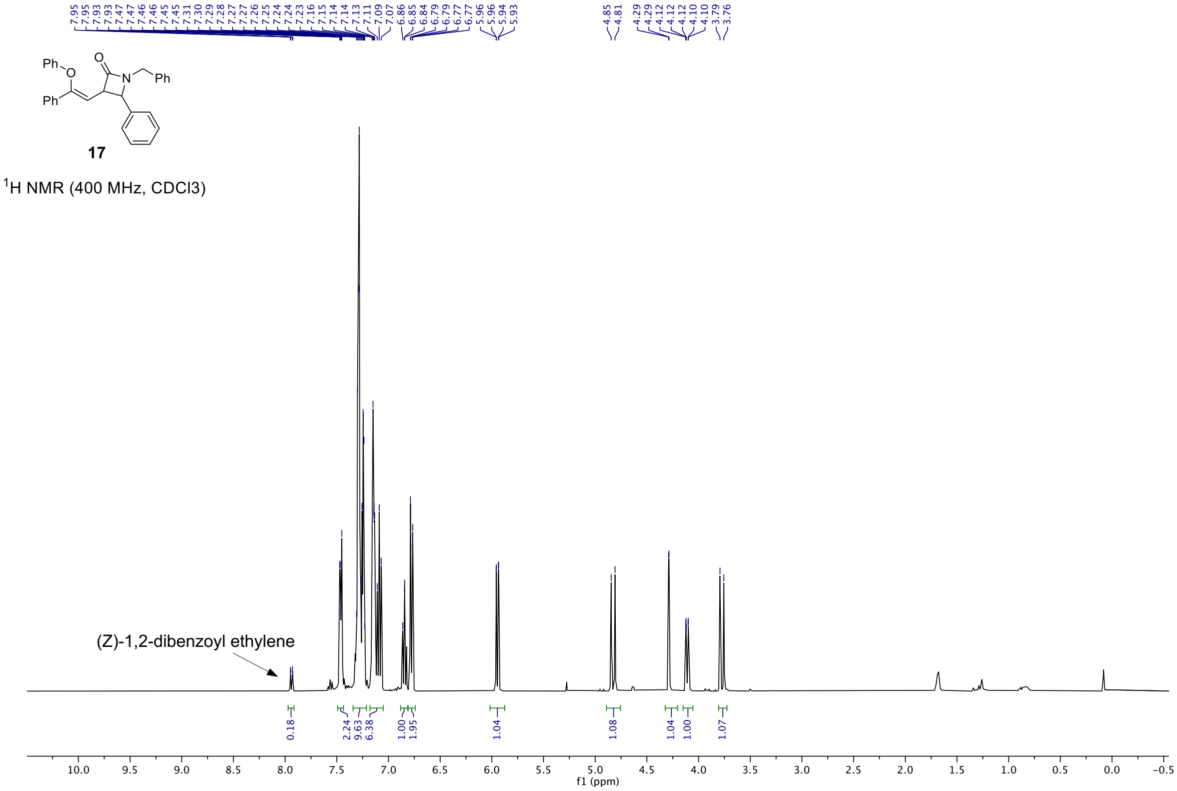

17

^{1}H NMR (400 MHz, CDCl3)

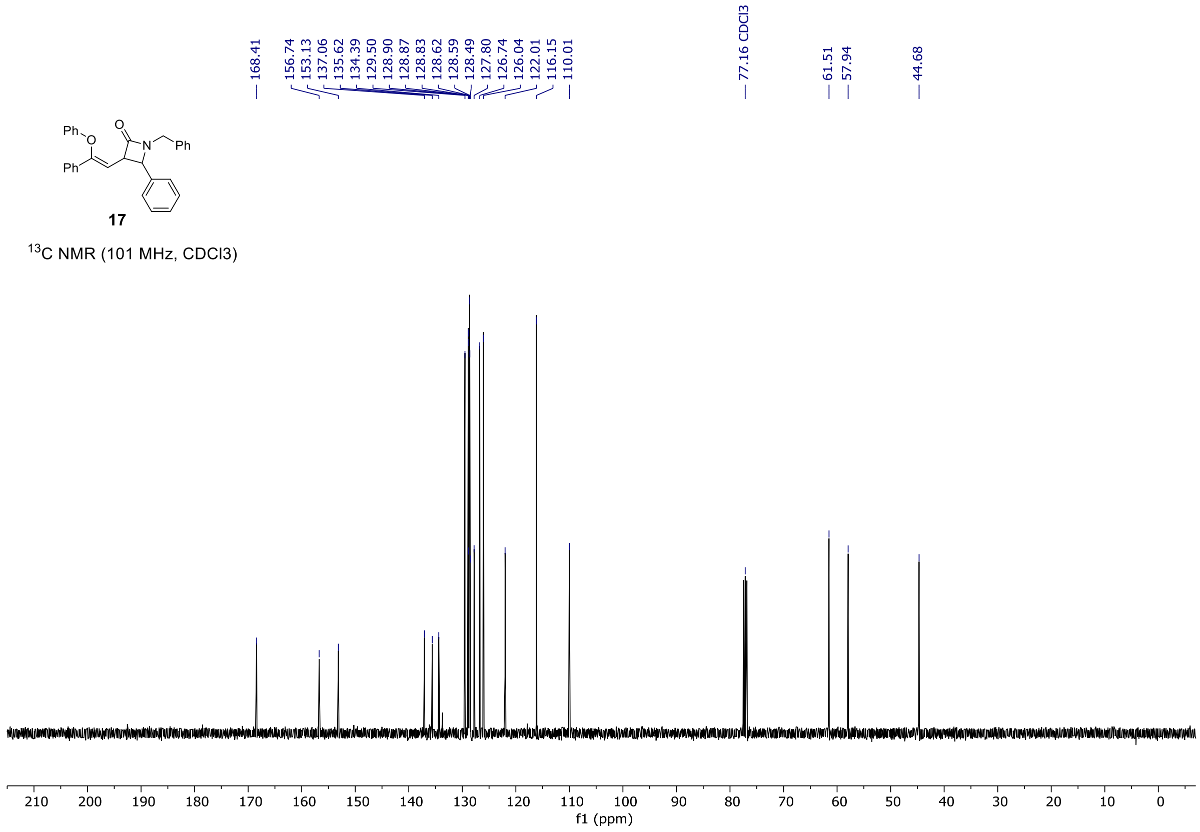

17

^{13}C NMR (101 MHz, CDCl3)

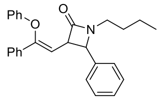

18

^{1}H NMR (400 MHz, CDCl3)

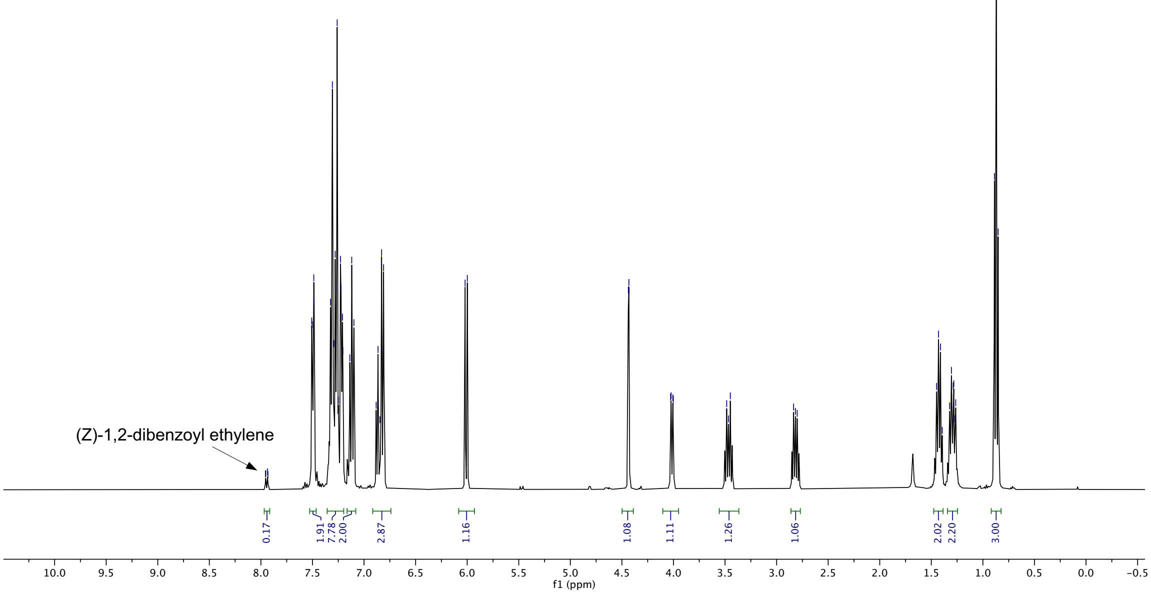

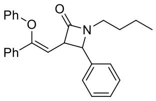

18

^{13}C NMR (101 MHz, CDCl3)

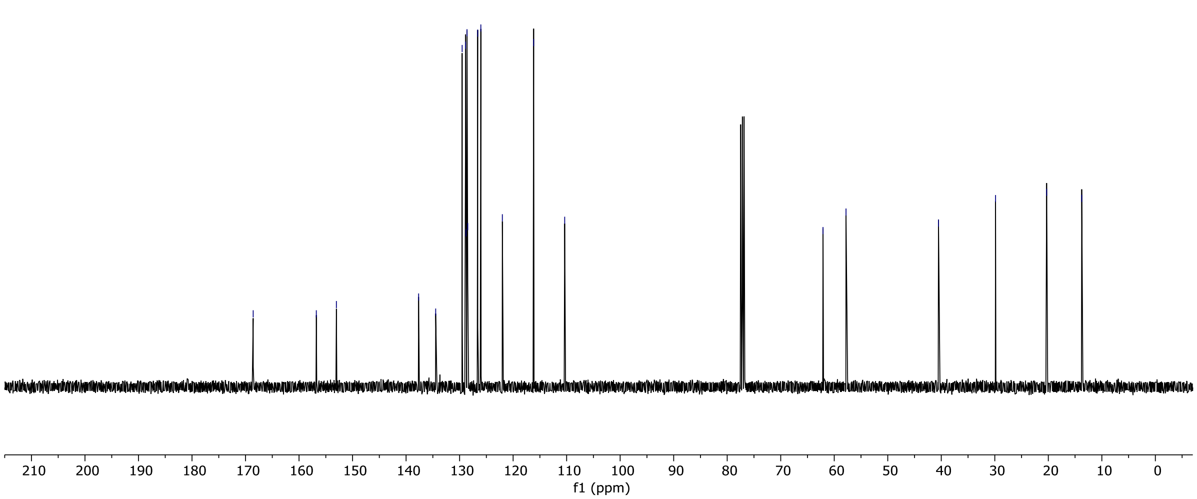

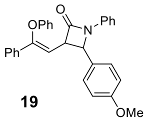

19

^{1}H NMR (400 MHz, CDCl3)

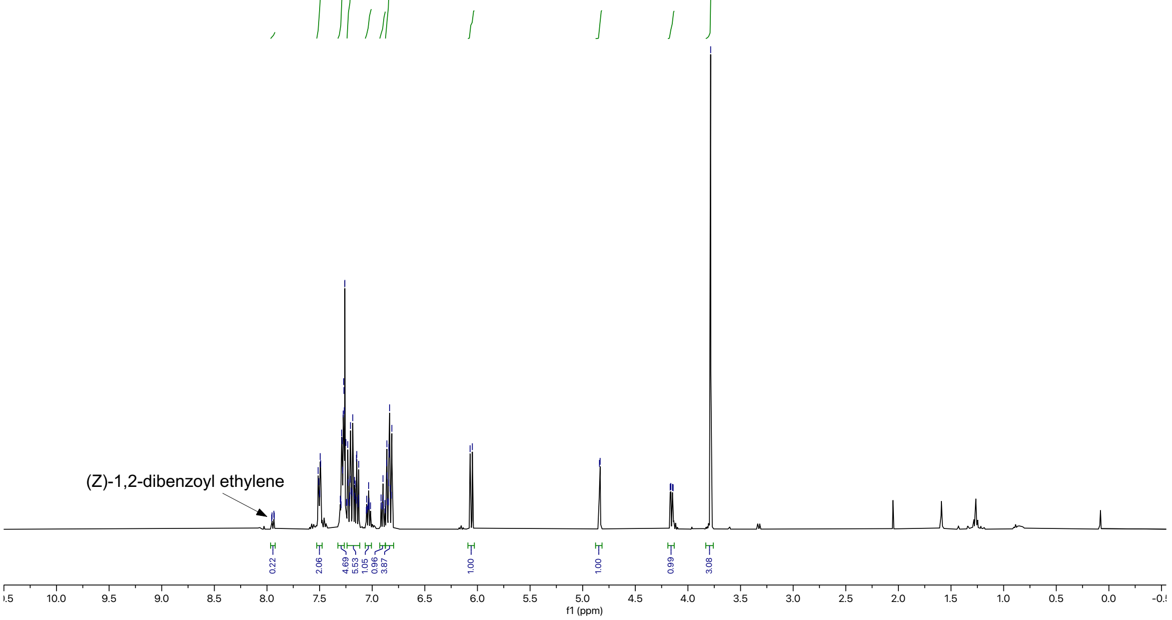

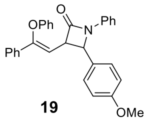

19

^{13}C NMR (101 MHz, CDCl3)

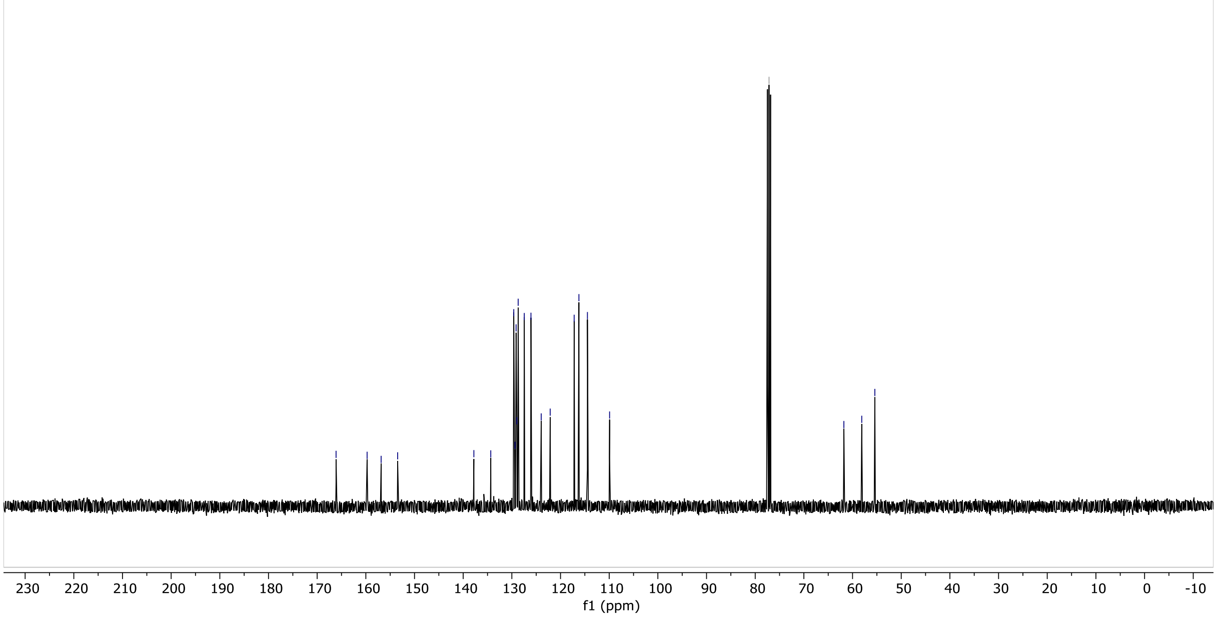

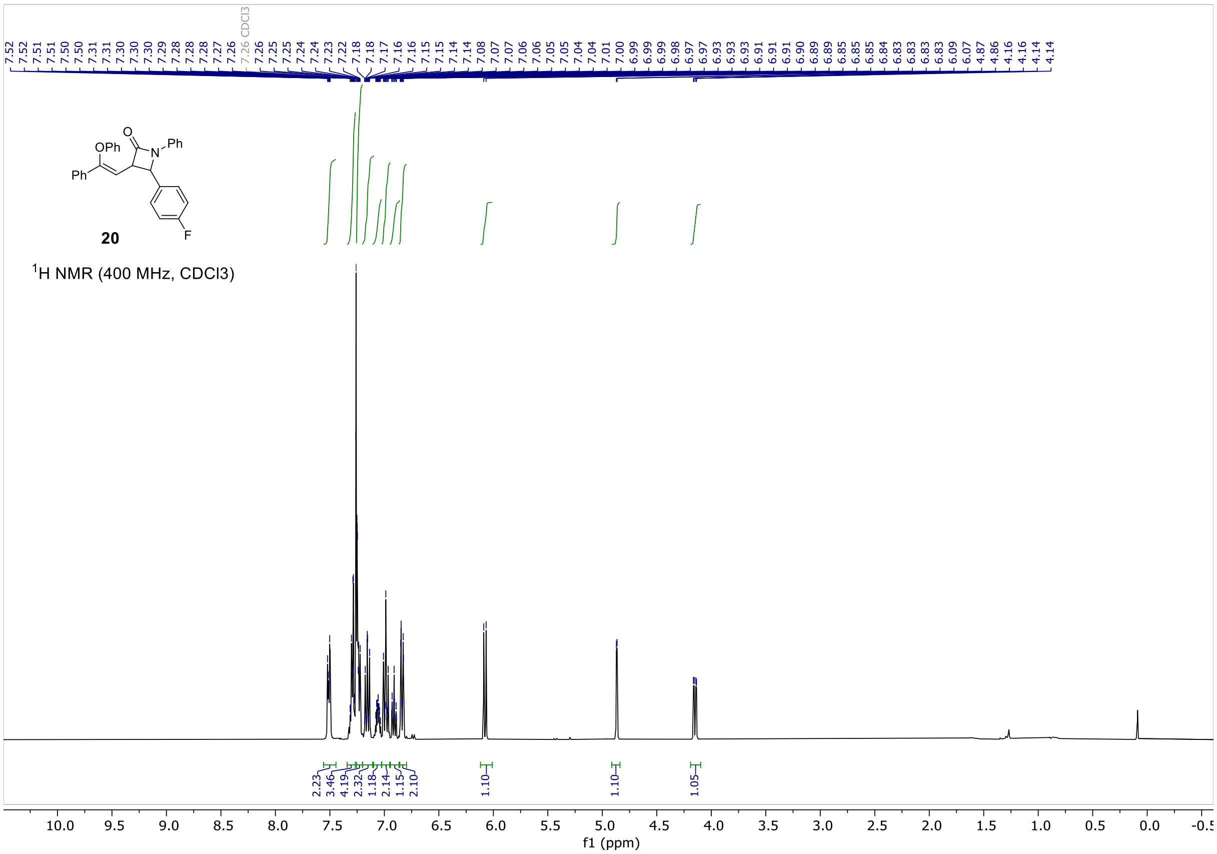

20

^{1}H NMR (400 MHz, CDCl3)

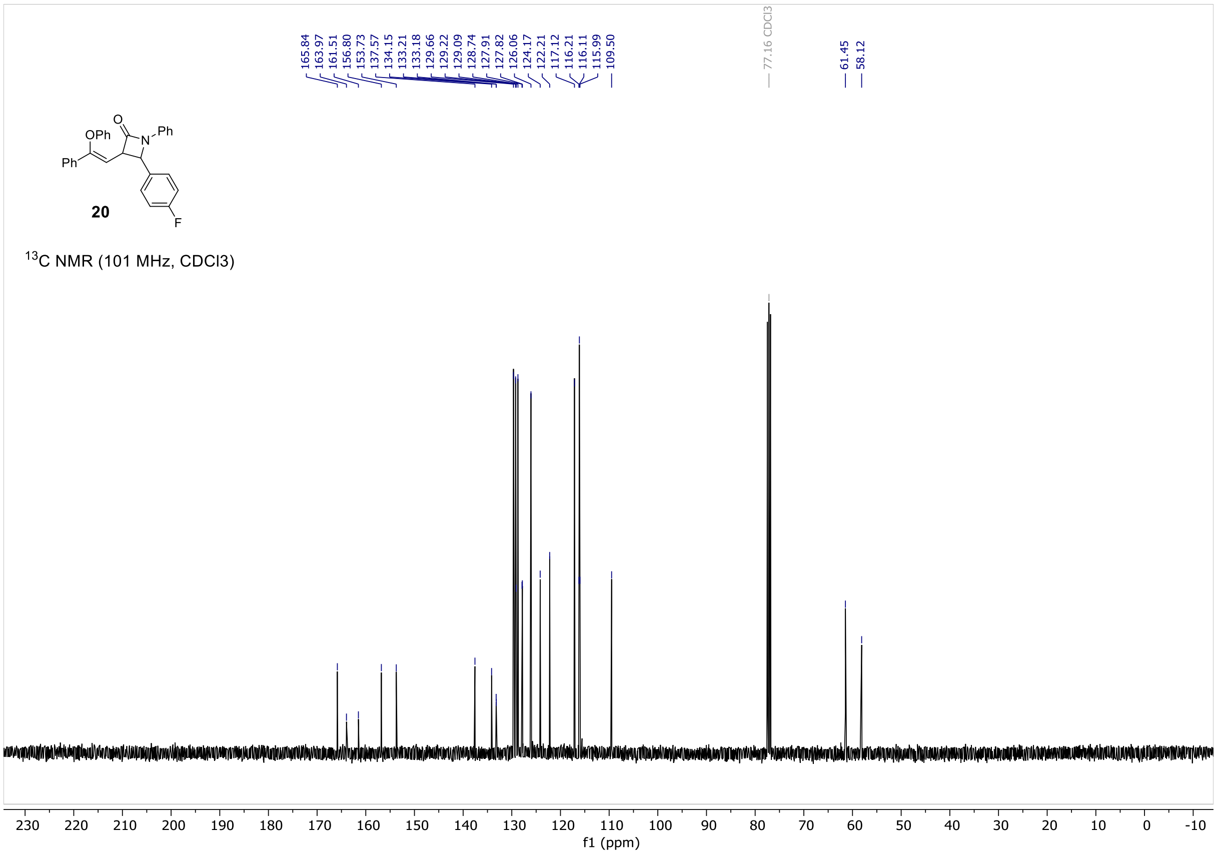

20

^{13}C NMR (101 MHz, CDCl3)

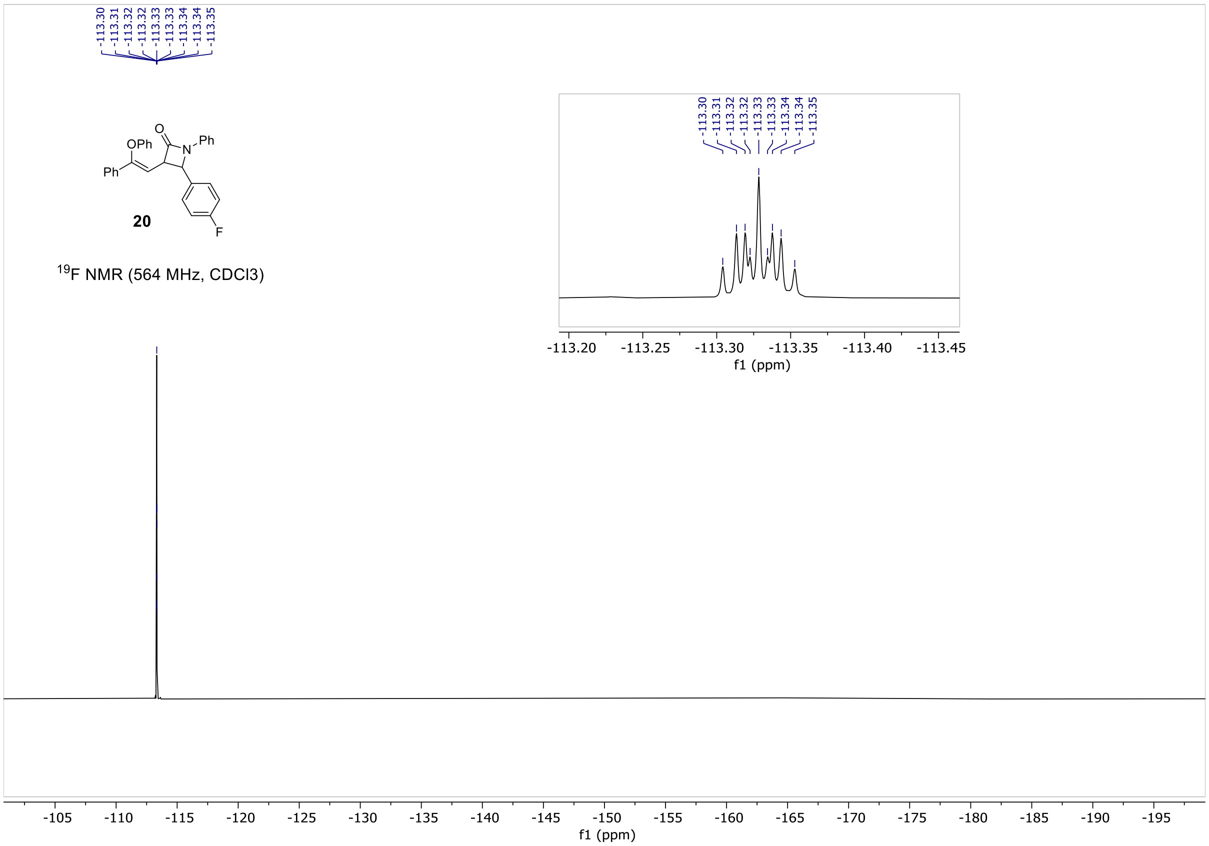

-113.30
-113.31
-113.32
-113.32
-113.33
-113.33
-113.34
-113.34
-113.35
OPh
O
N
Ph
Ph
20
F
19F NMR (564 MHz, CDCl3)
-113.20
-113.25
-113.30
-113.35
-113.40
-113.45
f1 (ppm)
-105
-110
-115
-120
-125
-130
-135
-140
-145
-150
-155
-160
-165
-170
-175
-180
-185
-190
-195
f1 (ppm)

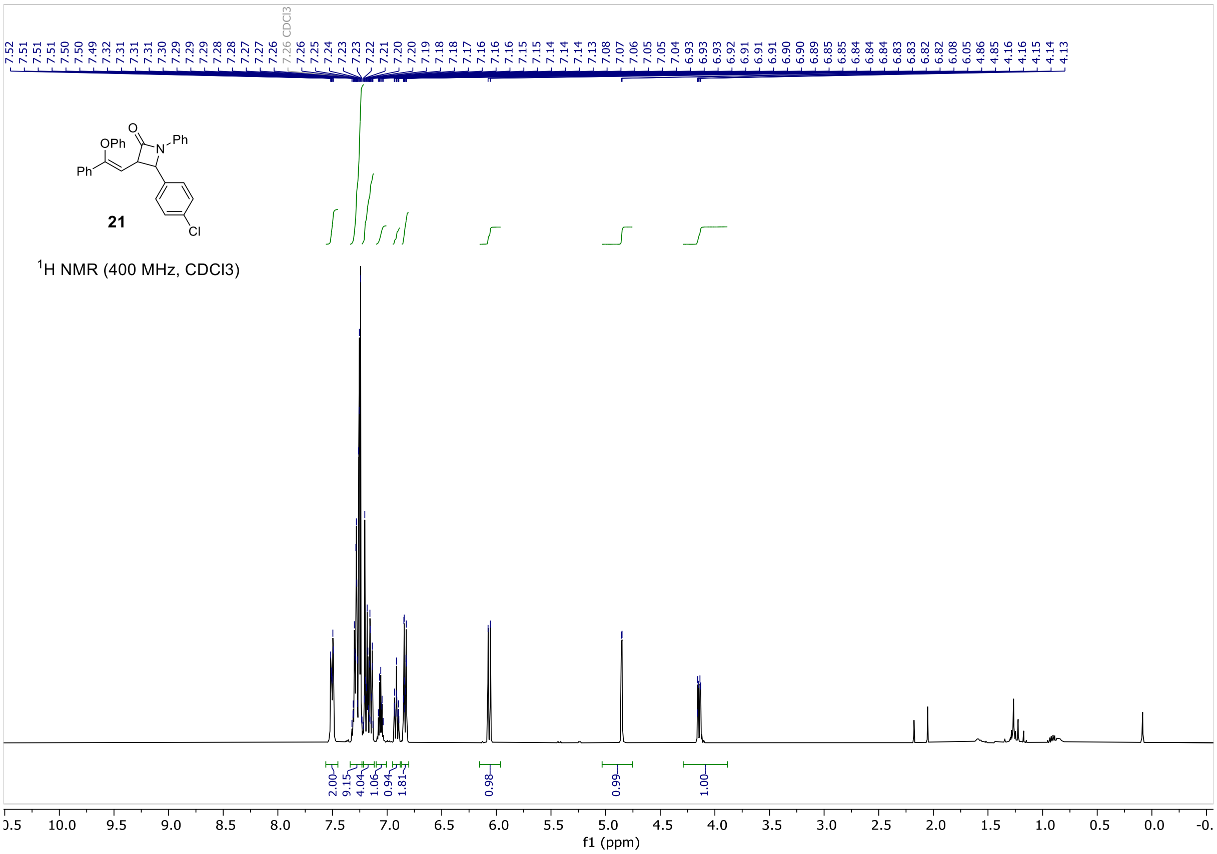

21
1H NMR (400 MHz, CDCl3)
f1 (ppm)

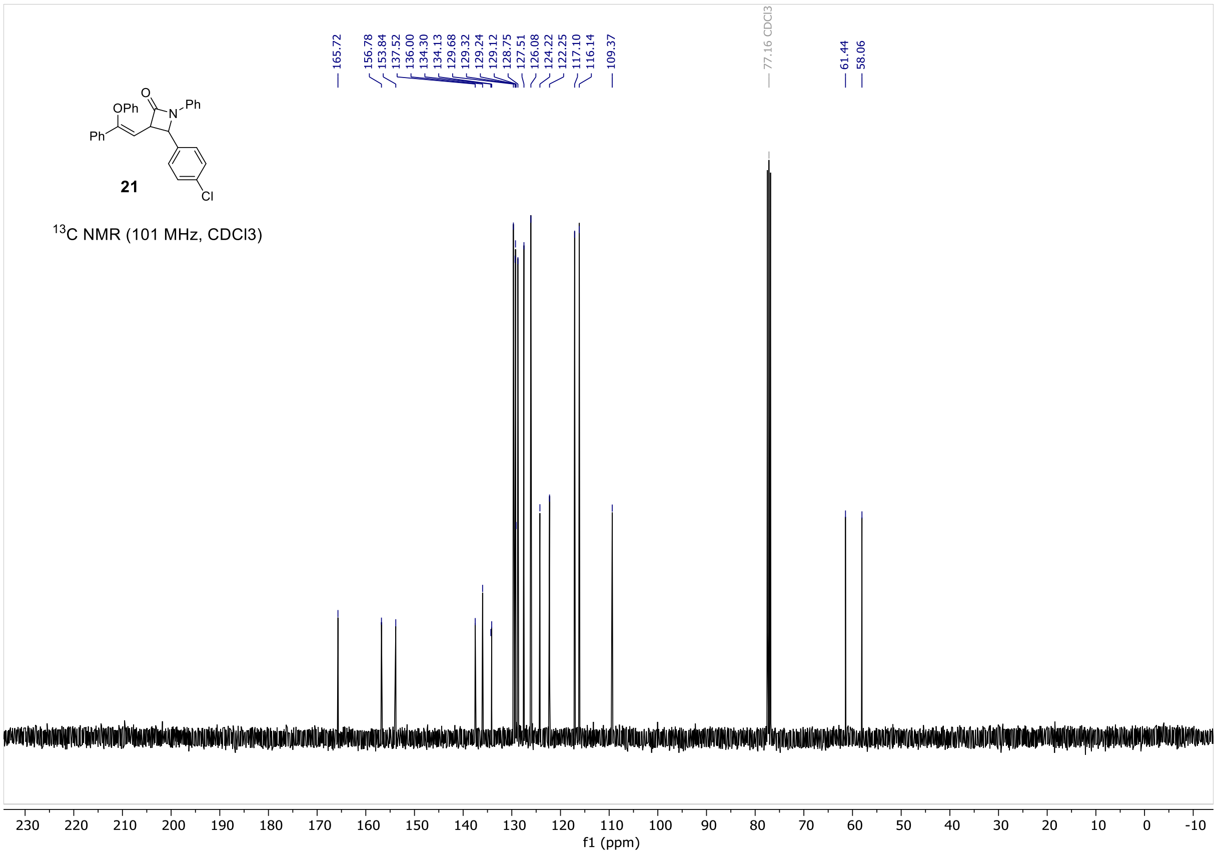

21
13C NMR (101 MHz, CDCl3)
165.72
156.78
153.84
137.52
136.00
134.30
134.13
129.68
129.32
129.24
129.12
128.75
127.51
126.08
124.22
122.25
117.10
116.14
109.37
77.16 CDCl3
61.44
58.06
f1 (ppm)

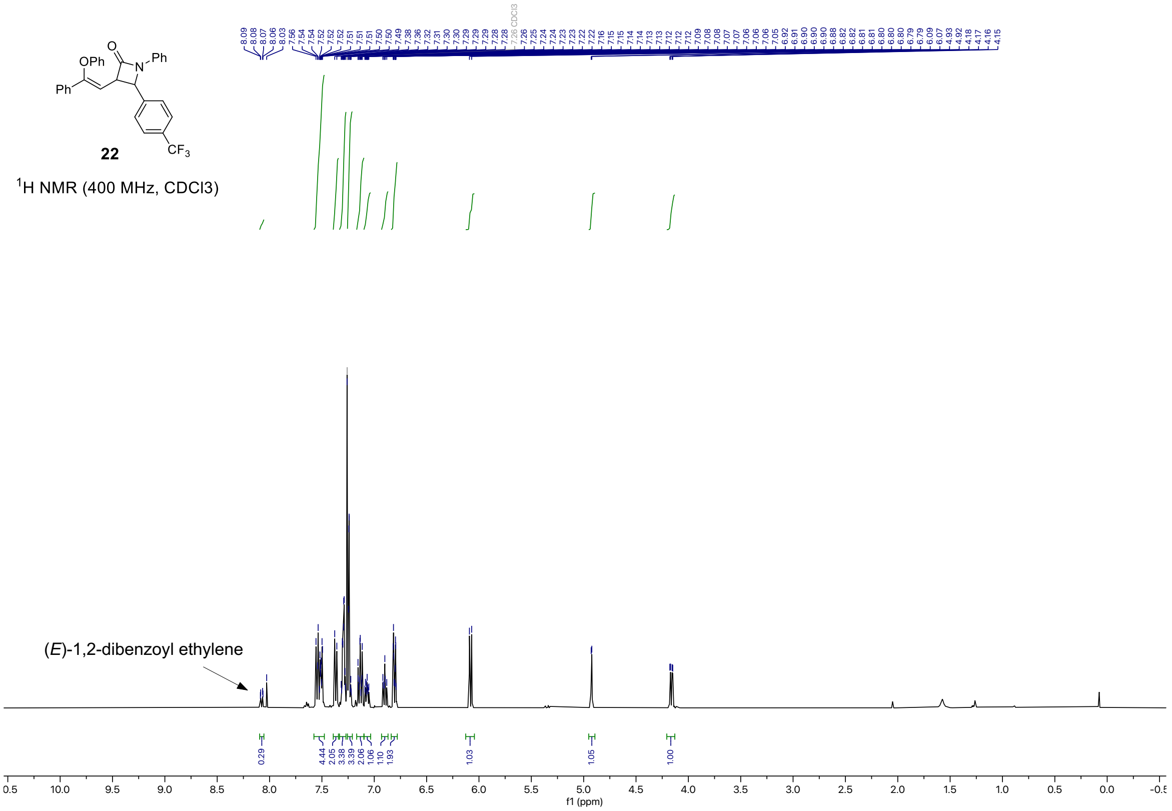

22

^{1}H NMR (400 MHz, CDCl3)

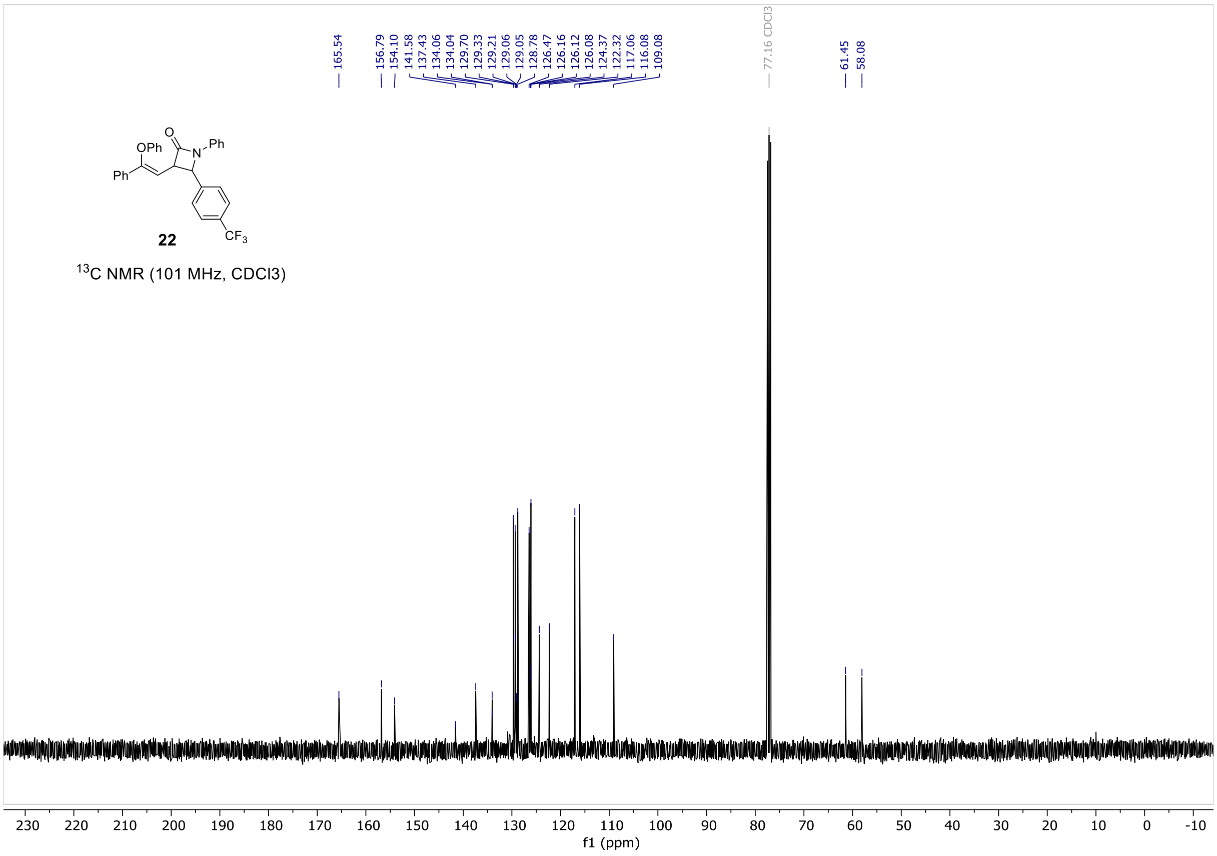

22

^{13}C NMR (101 MHz, CDCl3)

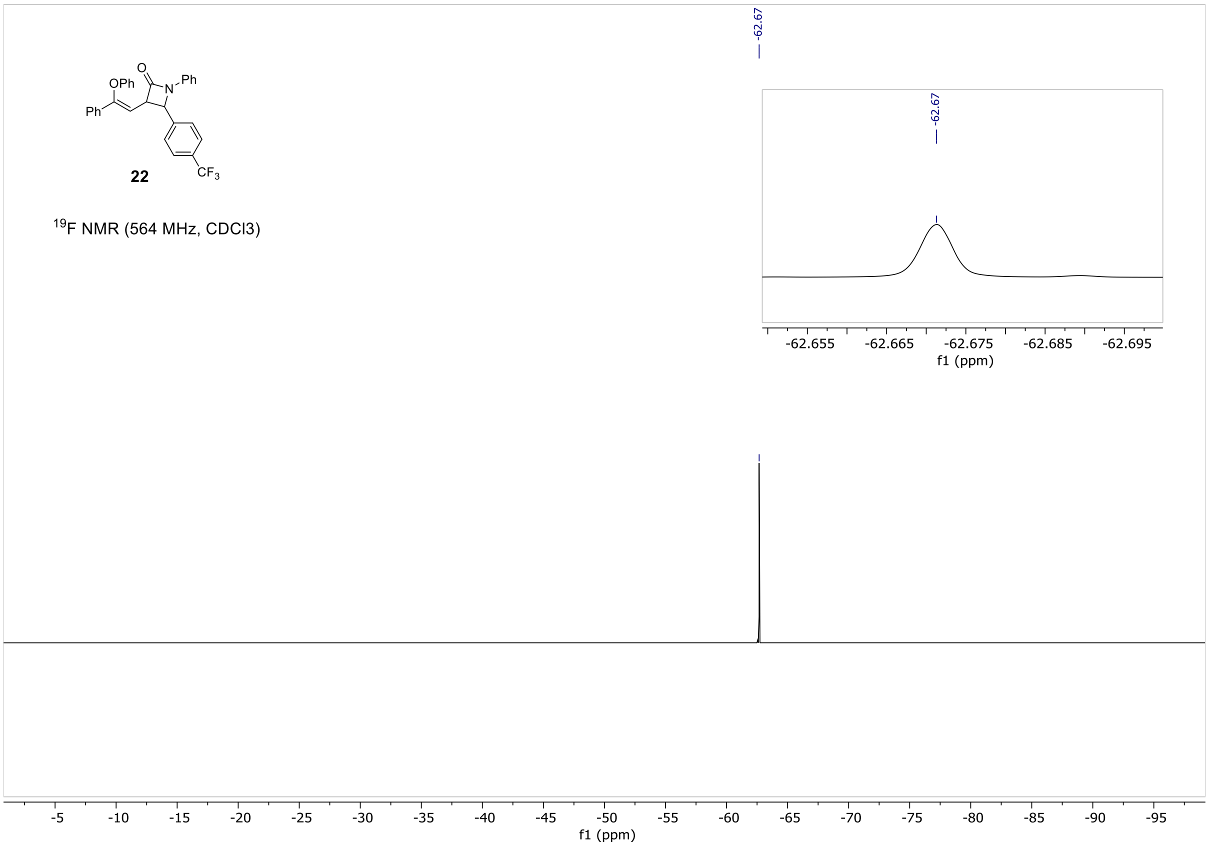

OPh
O
N
Ph
Ph
CF3
22
19F NMR (564 MHz, CDCl3)
-62.67
-62.655
-62.665
-62.675
-62.685
-62.695
f1 (ppm)
-5
-10
-15
-20
-25
-30
-35
-40
-45
-50
-55
-60
-65
-70
-75
-80
-85
-90
-95
f1 (ppm)

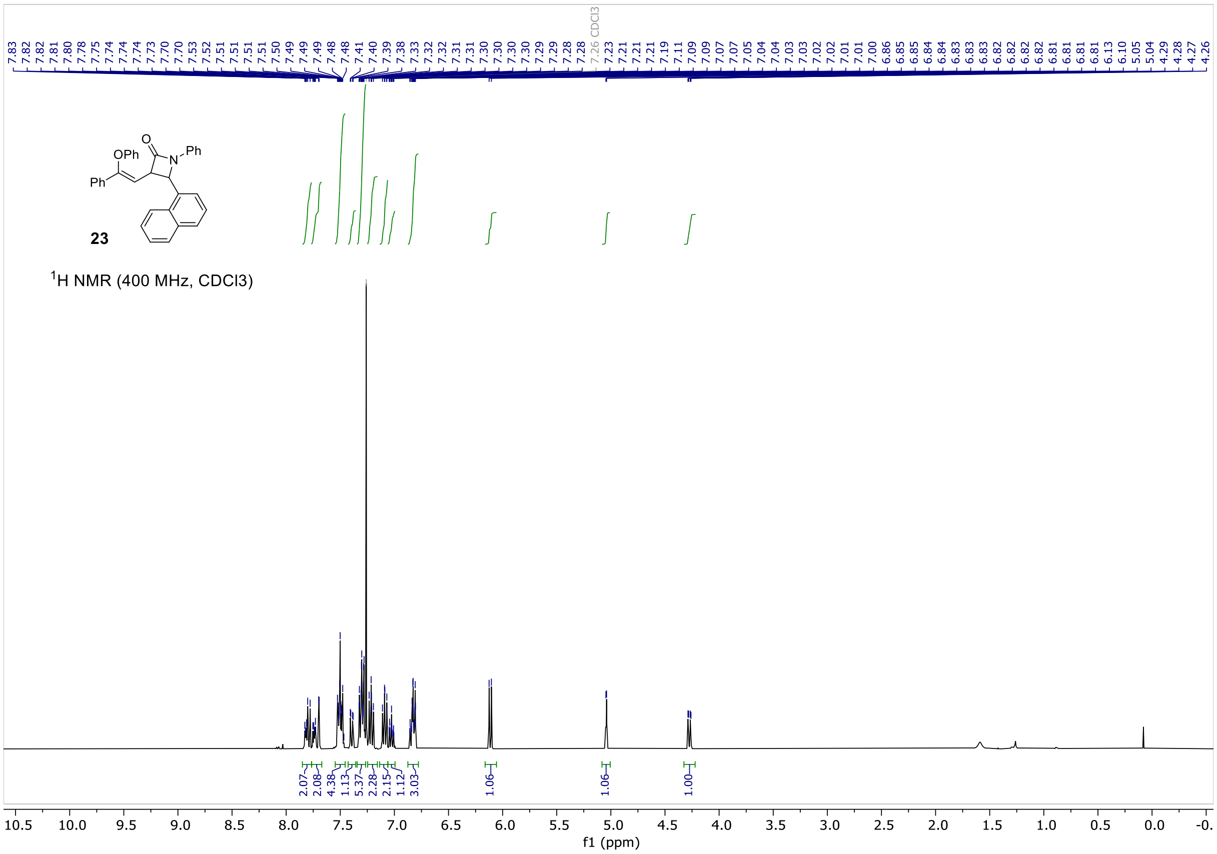
7.83 7.82 7.81 7.80 7.78 7.75 7.74 7.74 7.73 7.70 7.70 7.53 7.52 7.51 7.51 7.51 7.50 7.49 7.49 7.49 7.48 7.41 7.40 7.39 7.38 7.33 7.32 7.32 7.31 7.30 7.30 7.30 7.30 7.29 7.29 7.28 7.28 7.26 CDCl3 7.23 7.21 7.21 7.19 7.11 7.09 7.09 7.07 7.07 7.05 7.04 7.04 7.03 7.03 7.02 7.02 7.01 7.01 7.00 6.86 6.85 6.85 6.84 6.83 6.83 6.83 6.82 6.82 6.82 6.81 6.81 6.81 6.81 6.13 6.10 5.05 5.04 4.29 4.28 4.27 4.26
OPh
O
N
Ph
Ph
23
1H NMR (400 MHz, CDCl3)
2.07 2.08 4.38 1.13 5.37 2.28 2.15 1.12 3.03 1.06 1.06 1.00
f1 (ppm)

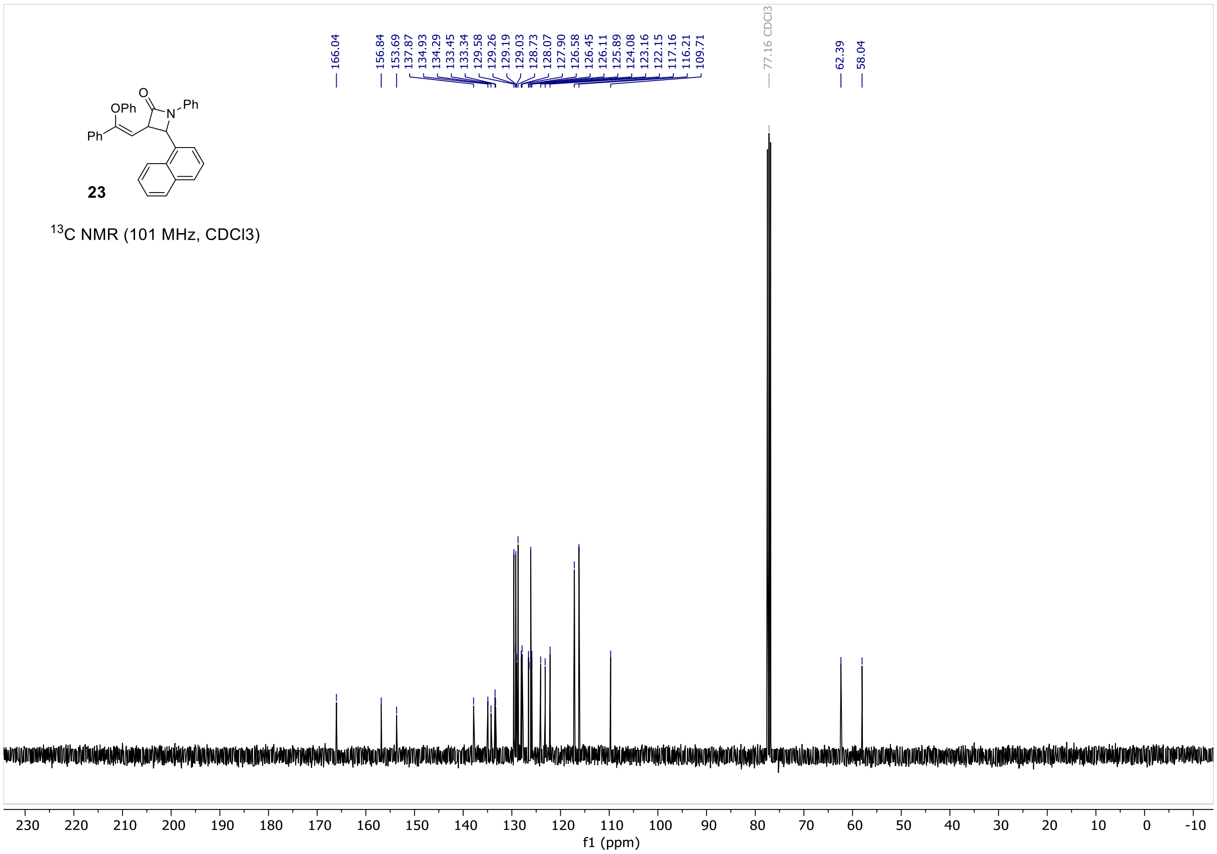
166.04 156.84 153.69 137.87 134.93 134.29 133.45 133.34 129.58 129.26 129.19 129.03 128.73 128.07 127.90 126.58 126.45 126.11 125.89 124.08 123.16 122.15 117.16 116.21 109.71 77.16 CDCl3 62.39 58.04
OPh
O
N
Ph
Ph
23
13C NMR (101 MHz, CDCl3)
f1 (ppm)

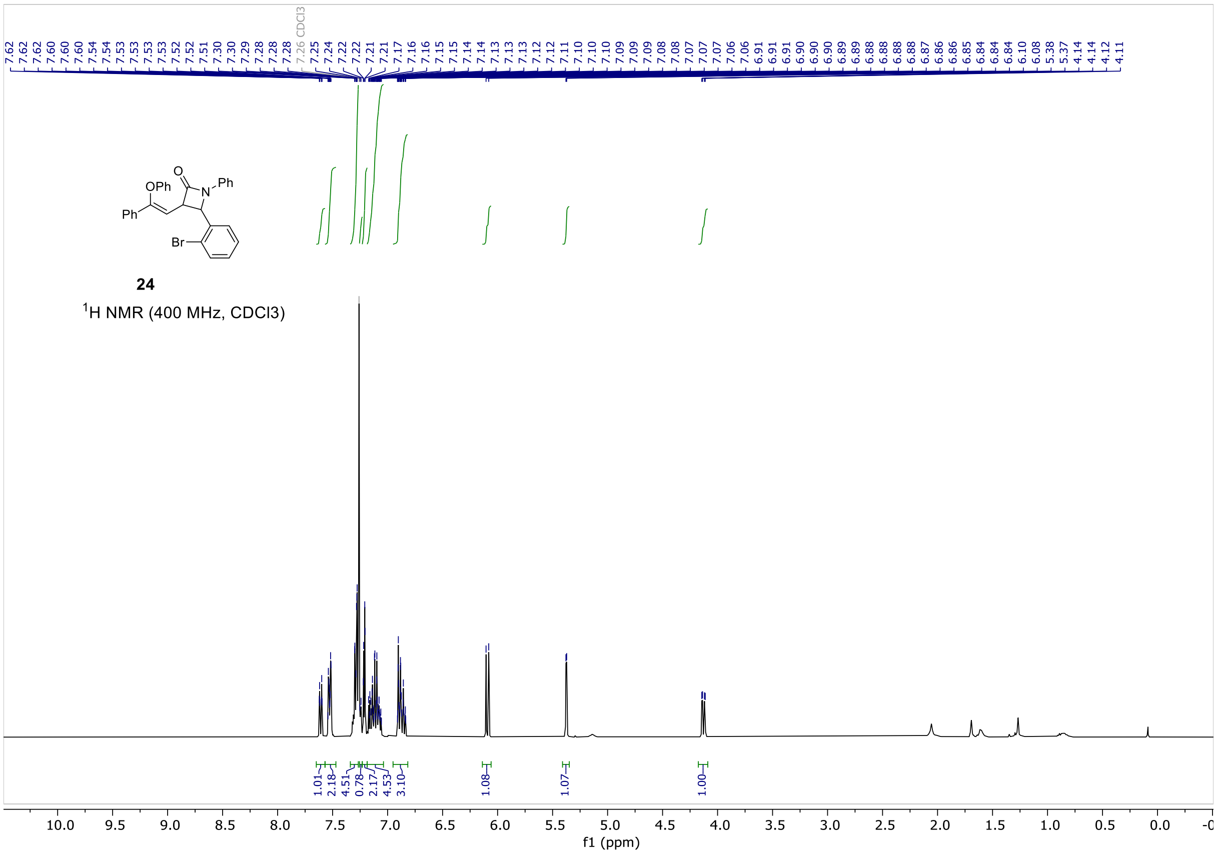

24

^{1}H NMR (400 MHz, CDCl3)

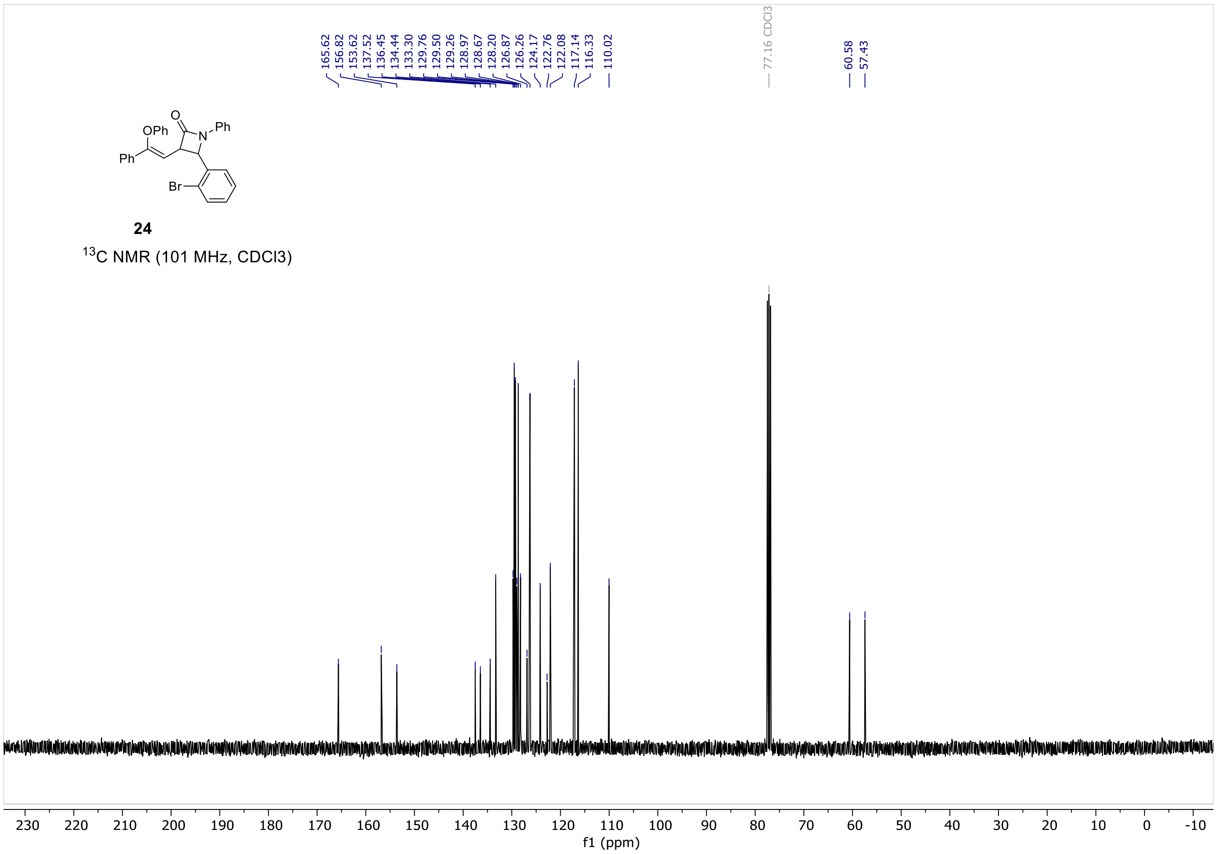

24

^{13}C NMR (101 MHz, CDCl3)

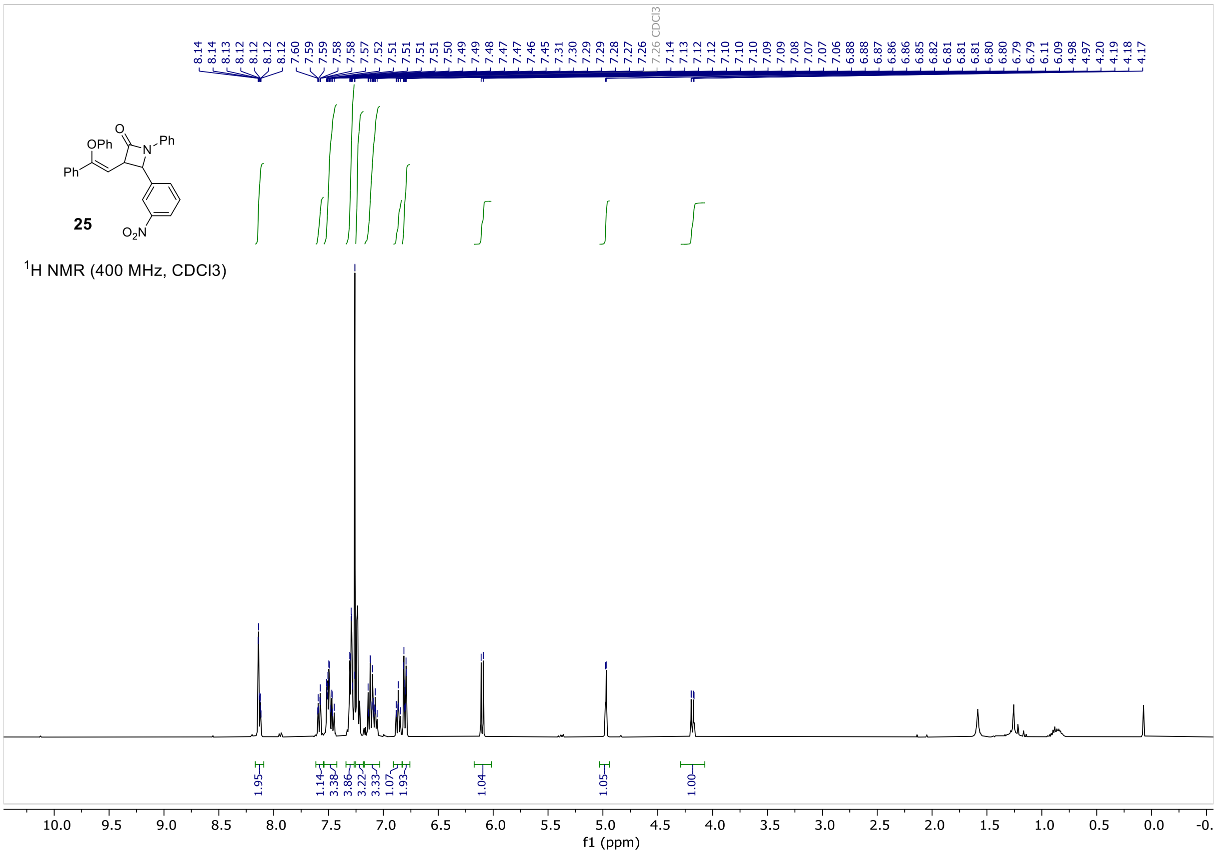
8.14 8.14 8.13 8.12 8.12 8.12 8.12 7.60 7.59 7.59 7.58 7.58 7.57 7.52 7.51 7.51 7.51 7.51 7.50 7.49 7.49 7.48 7.47 7.47 7.46 7.45 7.31 7.30 7.29 7.29 7.28 7.27 7.26 7.26 CDCl3 7.14 7.13 7.12 7.12 7.10 7.10 7.10 7.09 7.09 7.08 7.07 7.07 7.06 6.88 6.88 6.87 6.86 6.86 6.85 6.82 6.81 6.81 6.81 6.80 6.80 6.79 6.79 6.11 6.09 4.98 4.97 4.20 4.19 4.18 4.17
OPh
O
N
Ph
Ph
25
O_2N
^{1}H NMR (400 MHz, CDCl3)
1.95 1.14 3.38 3.86 3.22 3.33 1.07 1.93 1.04 1.05 1.00
f1 (ppm)

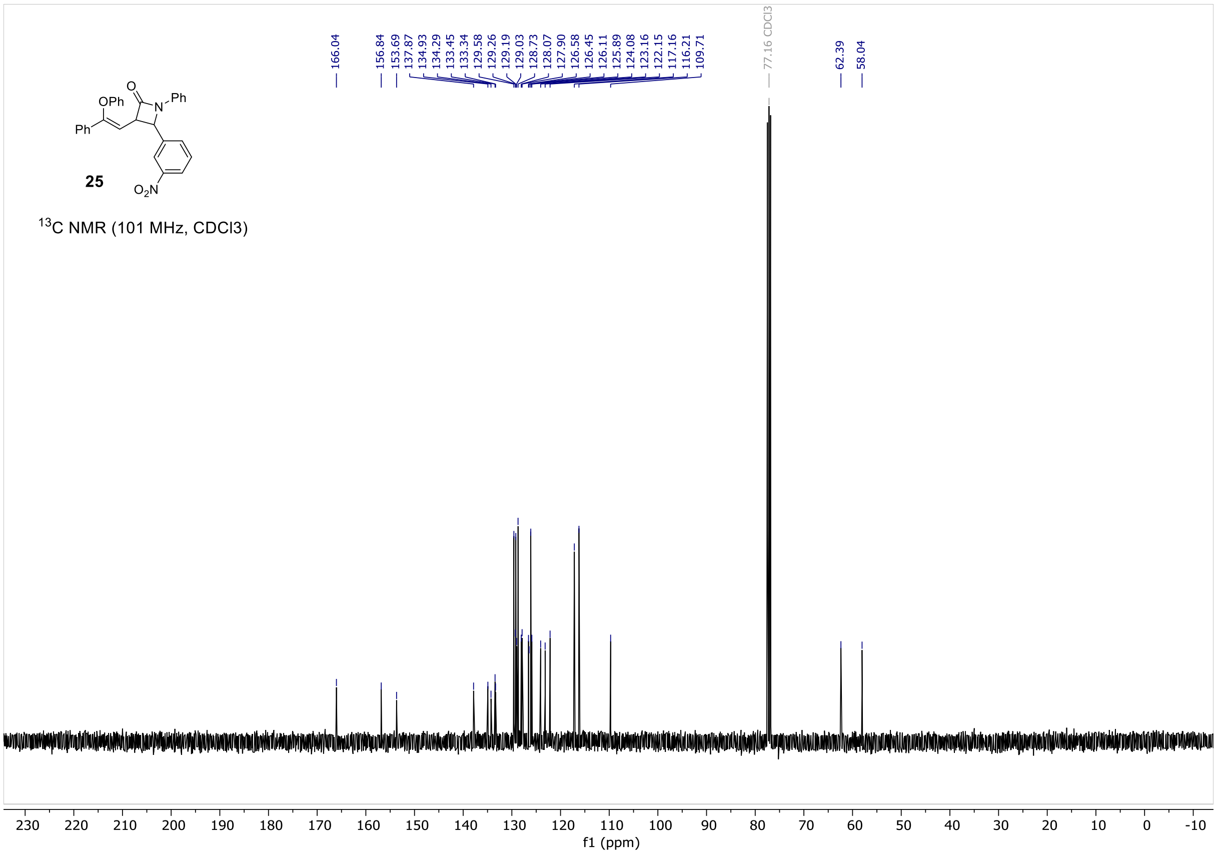
166.04 156.84 153.69 137.87 134.93 134.29 133.45 133.34 129.58 129.26 129.19 129.03 128.73 128.07 127.90 126.58 126.45 126.11 125.89 124.08 123.16 122.15 117.16 116.21 109.71 77.16 CDCl3 62.39 58.04
OPh
O
N
Ph
Ph
25
O_2N
^{13}C NMR (101 MHz, CDCl3)
f1 (ppm)

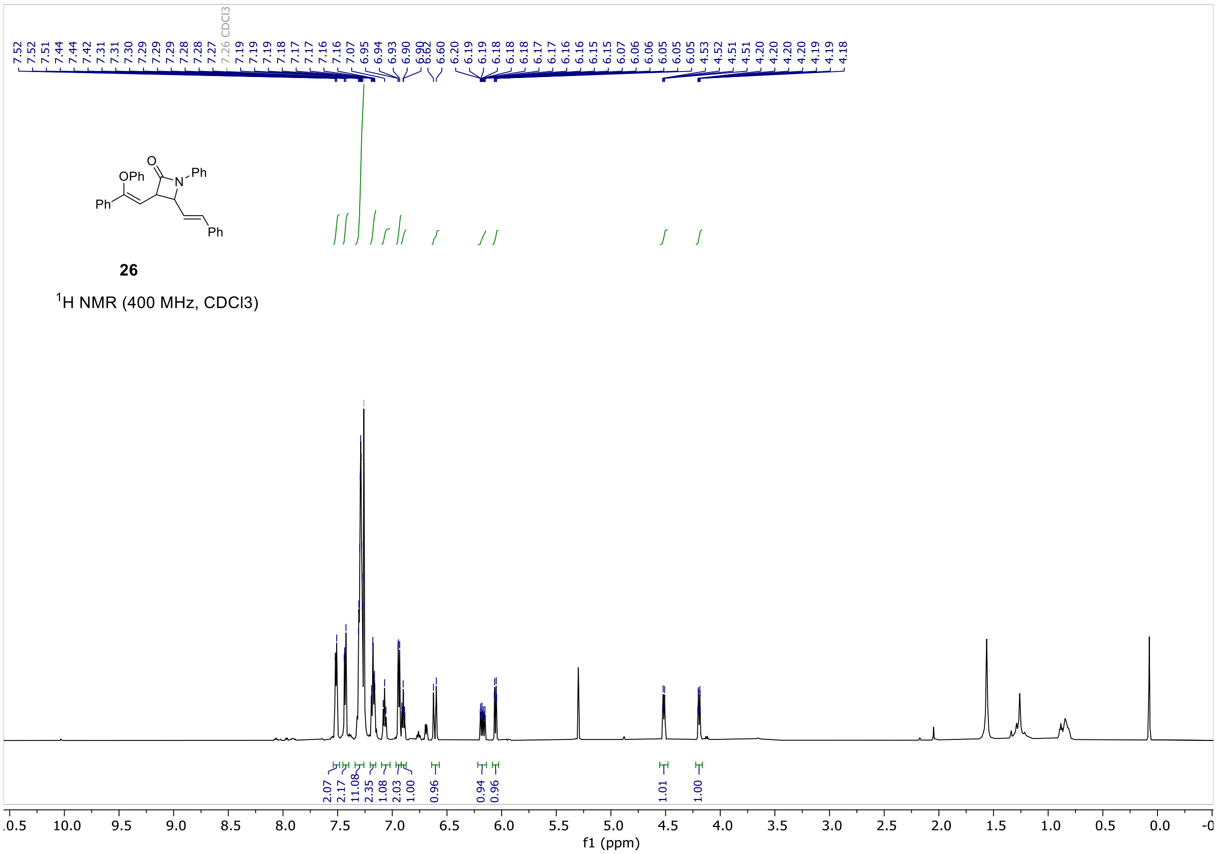

26
^{1}H NMR (400 MHz, CDCl3)
f1 (ppm)

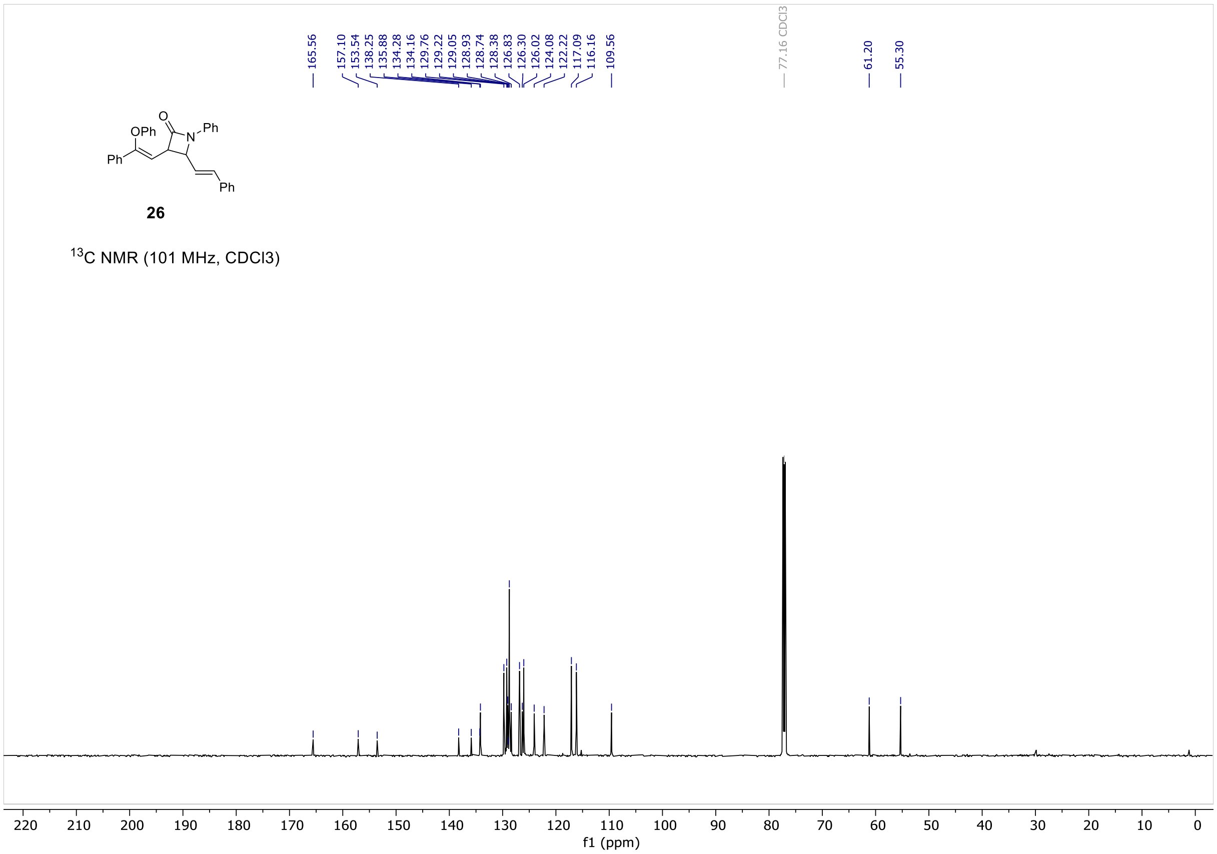

26
^{13}C NMR (101 MHz, CDCl3)
f1 (ppm)

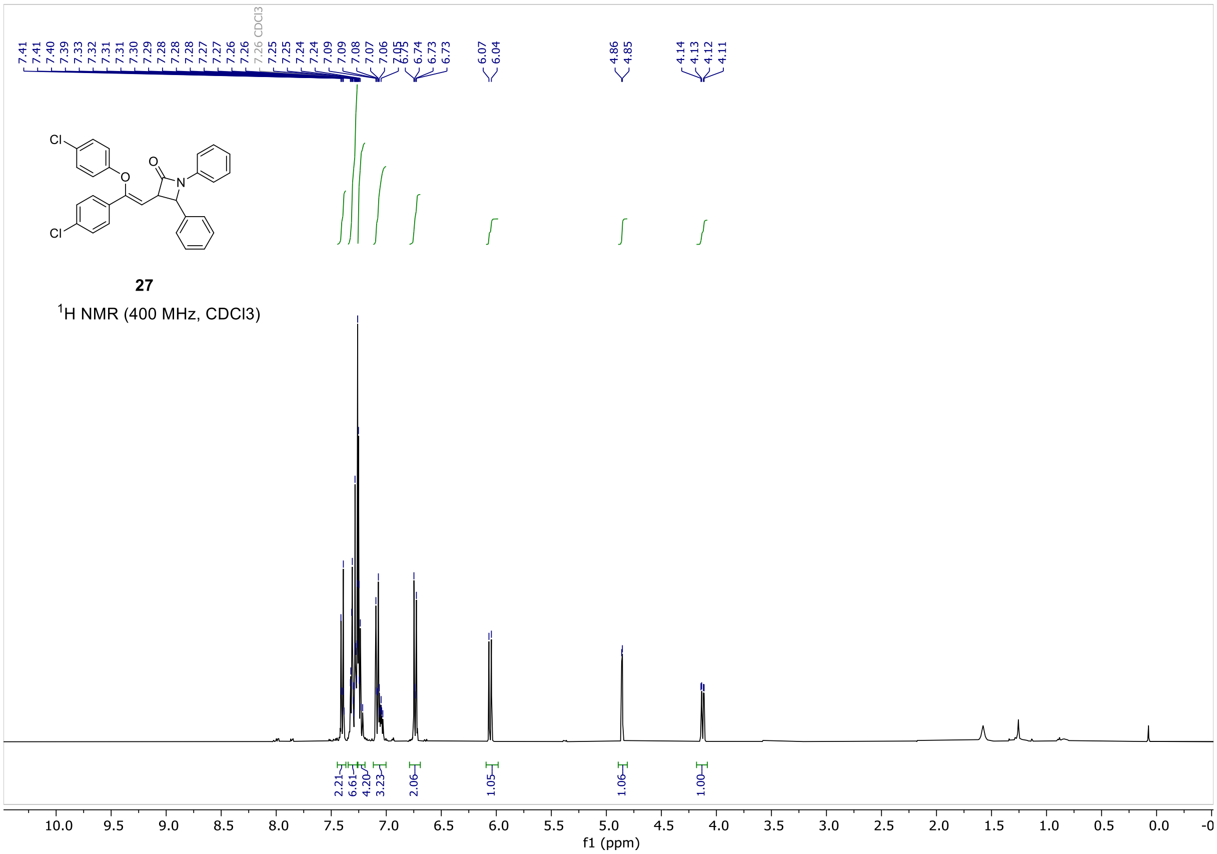
27
1H NMR (400 MHz, CDCl3)
f1 (ppm)

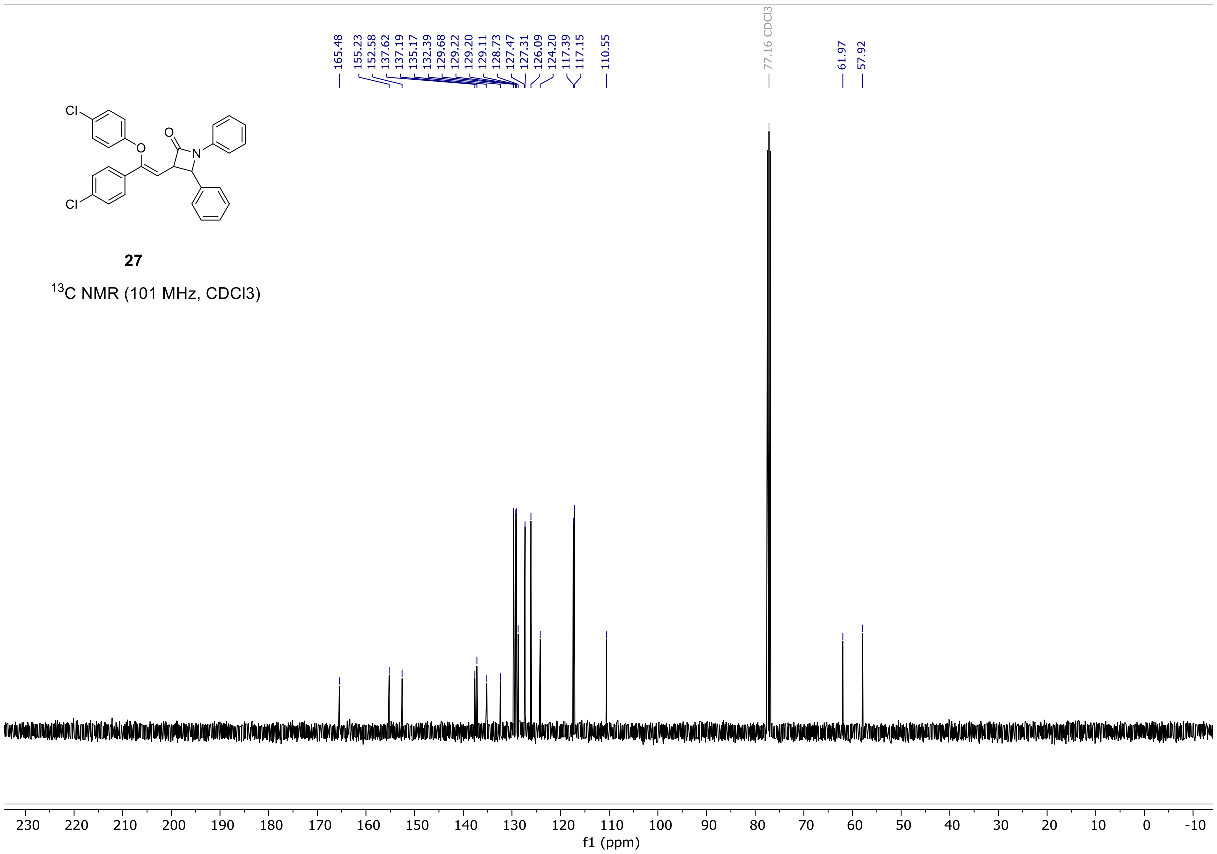
27
13C NMR (101 MHz, CDCl3)
f1 (ppm)

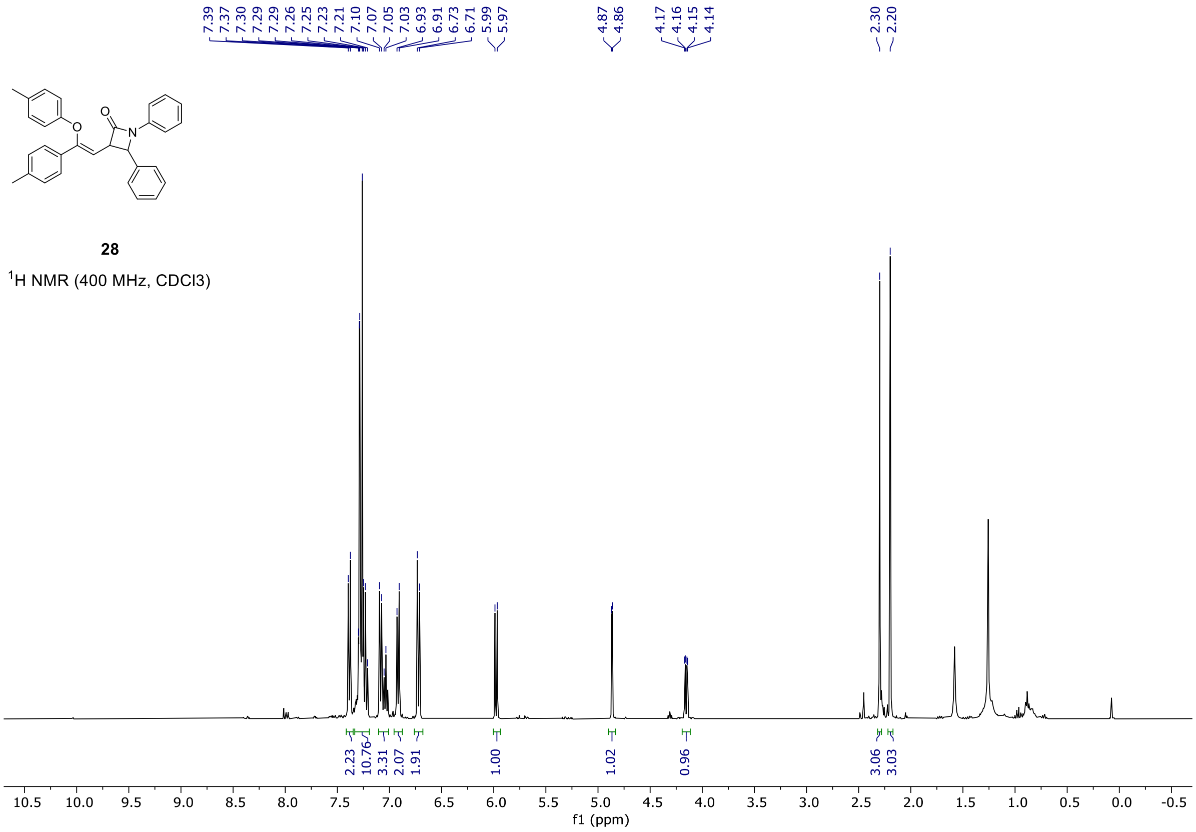
7.39 7.37 7.30 7.29 7.29 7.26 7.25 7.23 7.21 7.10 7.07 7.05 7.03 6.93 6.91 6.73 6.71 5.99 5.97
4.87 4.86
4.17 4.16 4.15 4.14
2.30 2.20
O
N
O
28
1H NMR (400 MHz, CDCl3)
2.23 10.76 3.31 2.07 1.91
1.00
1.02
0.96
3.06 3.03
10.5 10.0 9.5 9.0 8.5 8.0 7.5 7.0 6.5 6.0 5.5 5.0 4.5 4.0 3.5 3.0 2.5 2.0 1.5 1.0 0.5 0.0 -0.5
f1 (ppm)

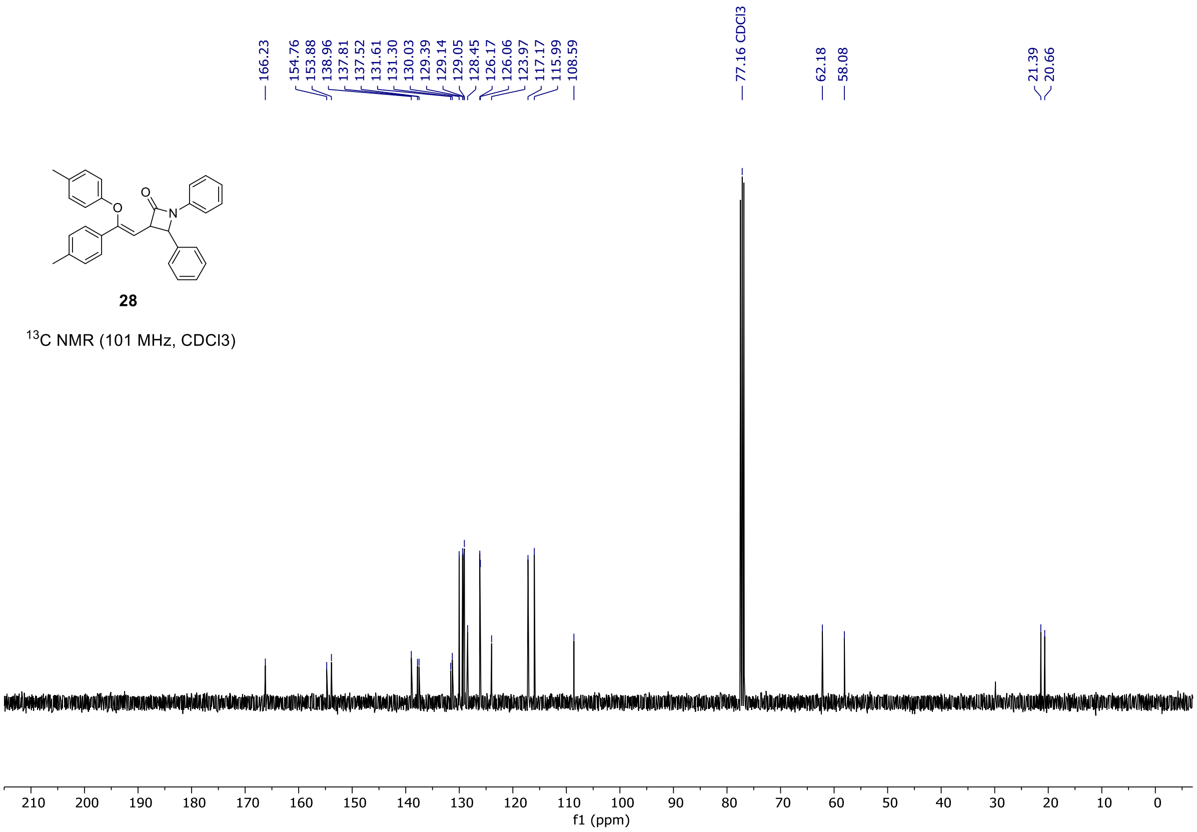
166.23
154.76 153.88 138.96 137.81 137.52 131.61 131.30 130.03 129.39 129.14 129.05 128.45 126.17 126.06 123.97 117.17 115.99 108.59
77.16 CDCl3
62.18 58.08
21.39 20.66
O
N
O
28
13C NMR (101 MHz, CDCl3)
210 200 190 180 170 160 150 140 130 120 110 100 90 80 70 60 50 40 30 20 10 0
f1 (ppm)

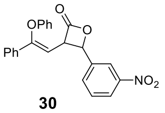

30

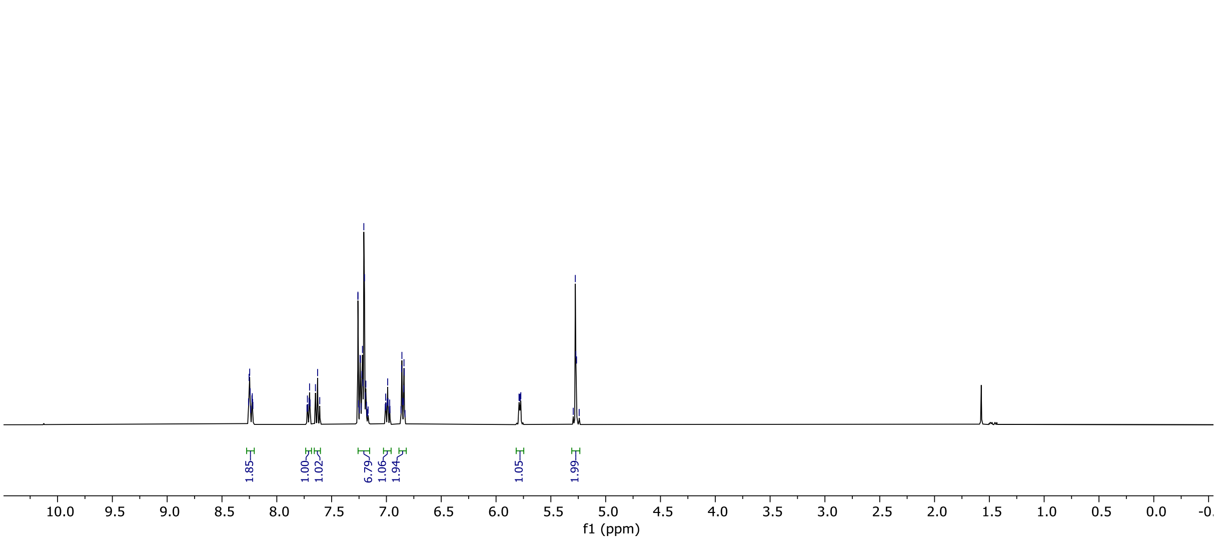

^{1}H NMR (400 MHz, CDCl3)

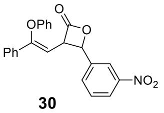

30

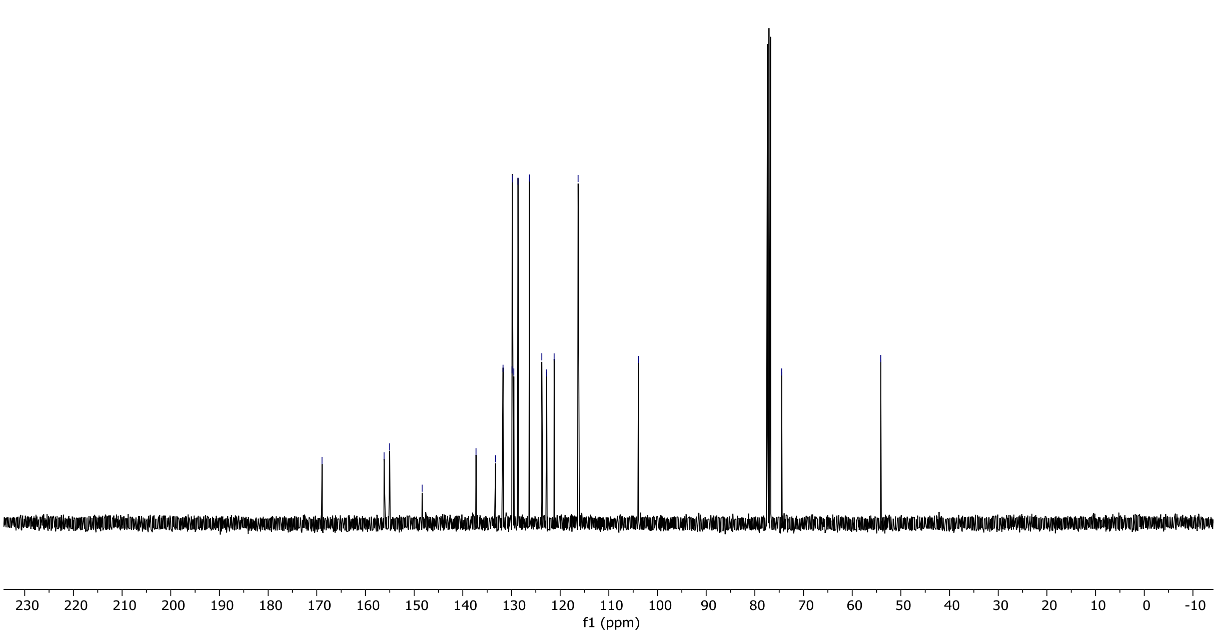

^{13}C NMR (101 MHz, CDCl3)

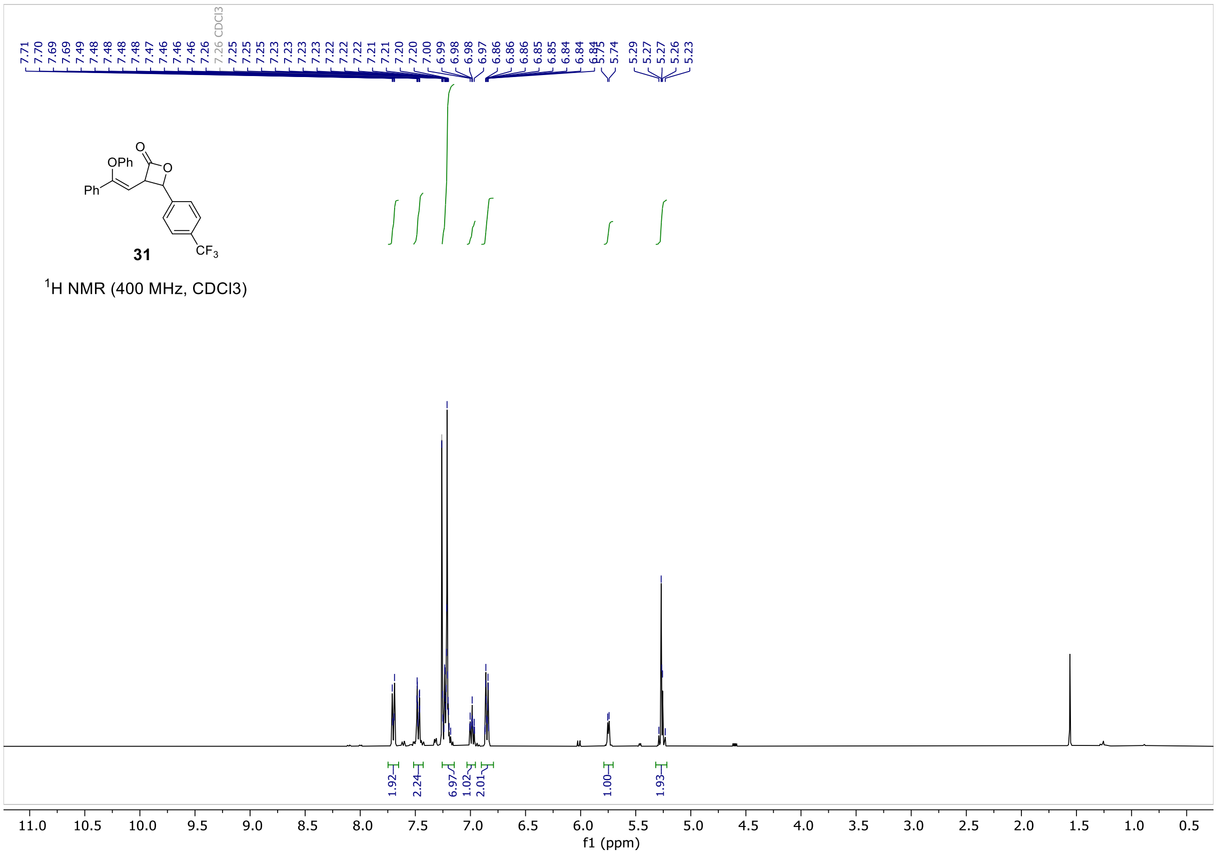
31
1H NMR (400 MHz, CDCl3)
f1 (ppm)

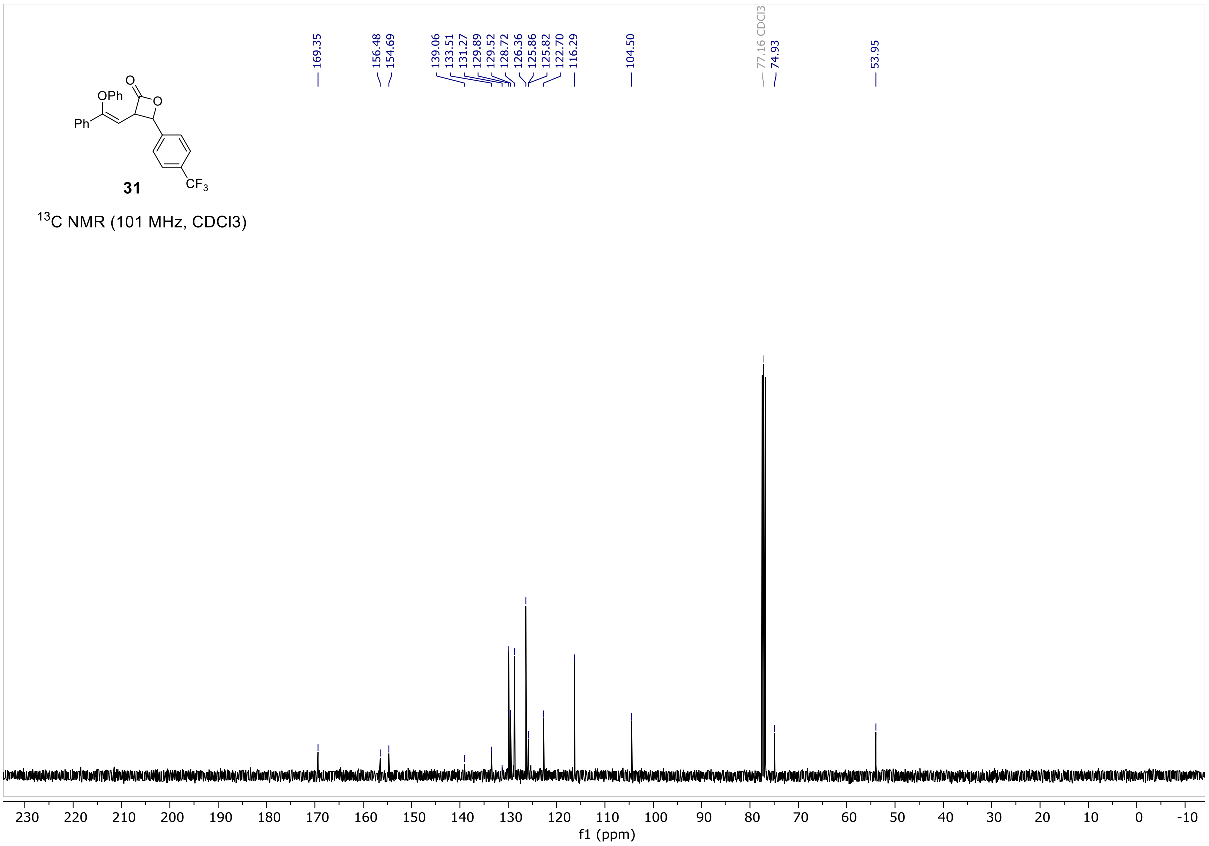
31
13C NMR (101 MHz, CDCl3)
f1 (ppm)

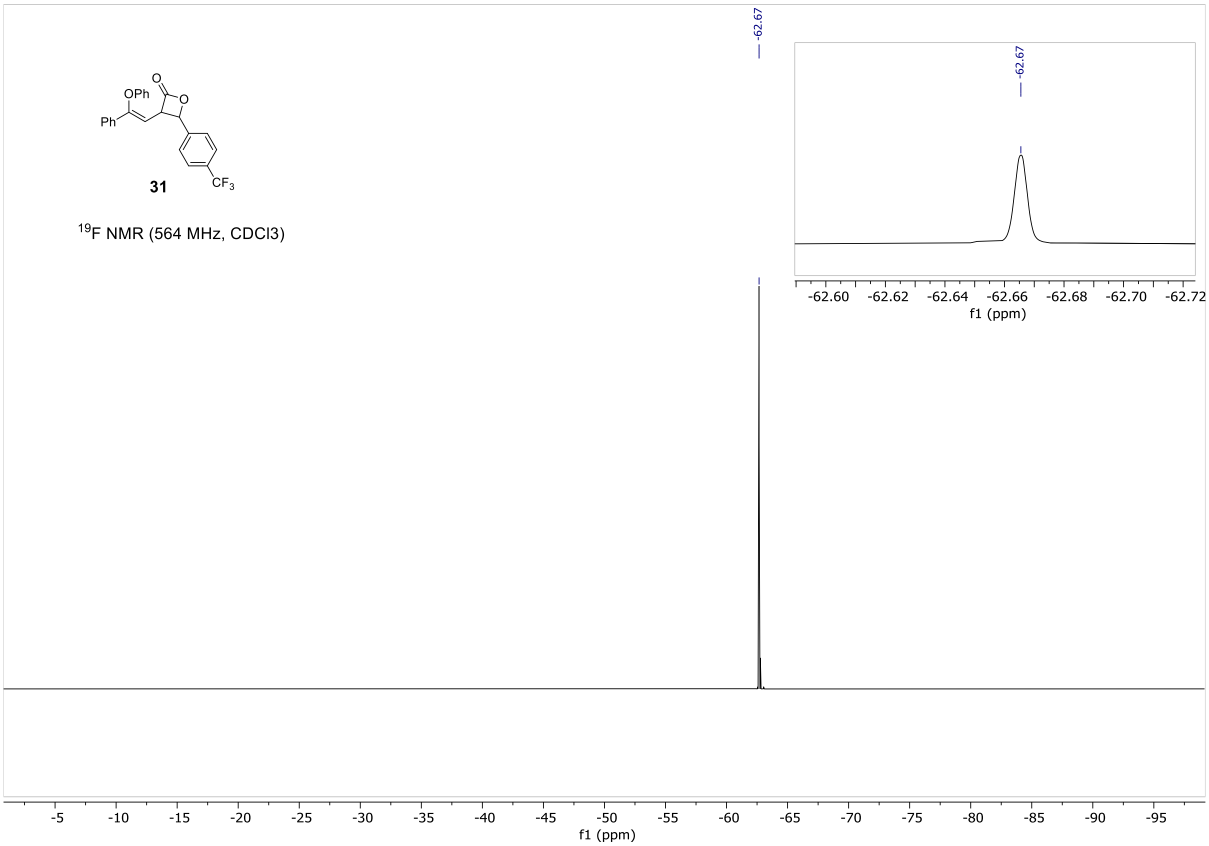

-62.67
OPh
O
O
Ph
31
CF3
19F NMR (564 MHz, CDCl3)
-62.67
-62.60
-62.62
-62.64
-62.66
-62.68
-62.70
-62.72
f1 (ppm)
-5
-10
-15
-20
-25
-30
-35
-40
-45
-50
-55
-60
-65
-70
-75
-80
-85
-90
-95
f1 (ppm)

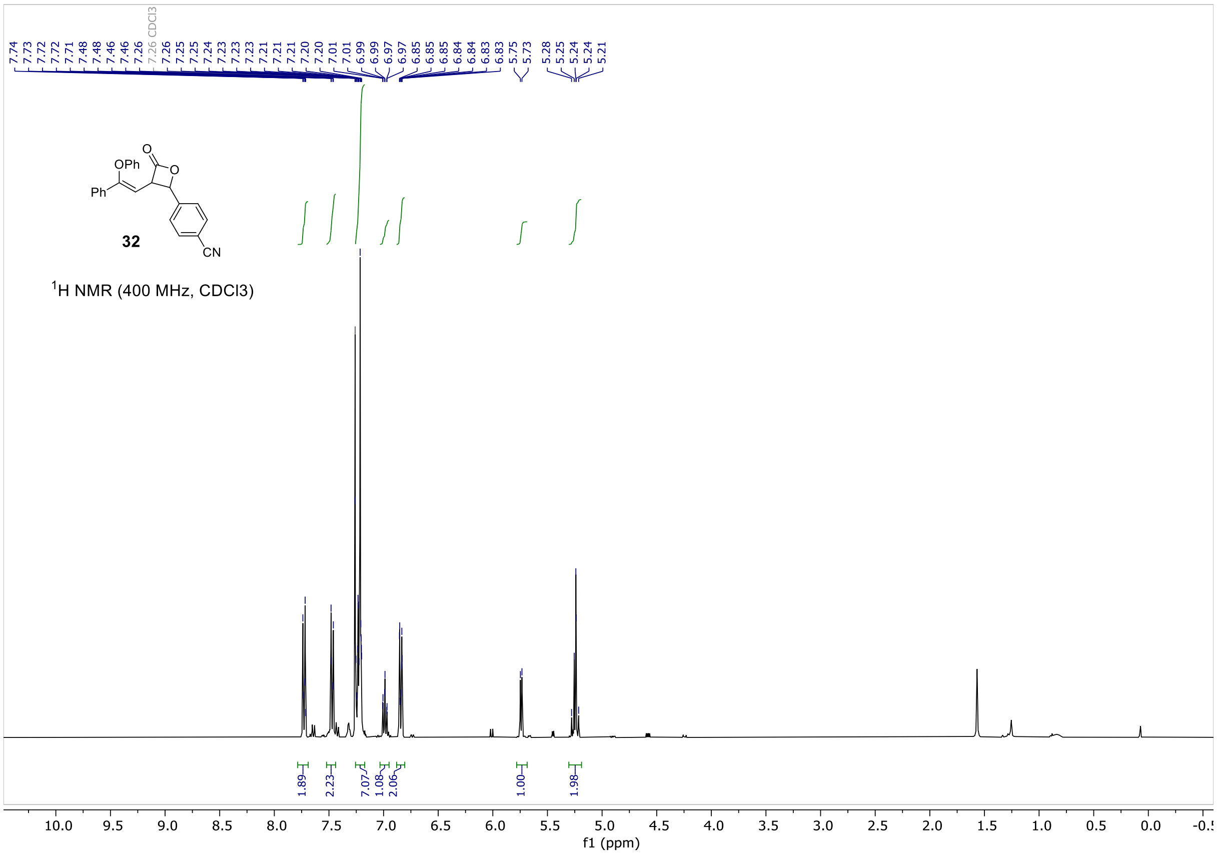
7.74
7.73
7.72
7.72
7.71
7.48
7.48
7.46
7.46
7.26
7.26 CDCl3
7.26
7.25
7.25
7.24
7.23
7.23
7.23
7.21
7.21
7.21
7.20
7.20
7.01
7.01
6.99
6.99
6.97
6.97
6.85
6.85
6.85
6.84
6.84
6.83
6.83
5.75
5.73
5.28
5.25
5.24
5.24
5.21
OPh
O
O
Ph
32
CN
1H NMR (400 MHz, CDCl3)
1.89
2.23
7.07
1.08
2.06
1.00
1.98
10.0
9.5
9.0
8.5
8.0
7.5
7.0
6.5
6.0
5.5
5.0
4.5
4.0
3.5
3.0
2.5
2.0
1.5
1.0
0.5
0.0
f1 (ppm)

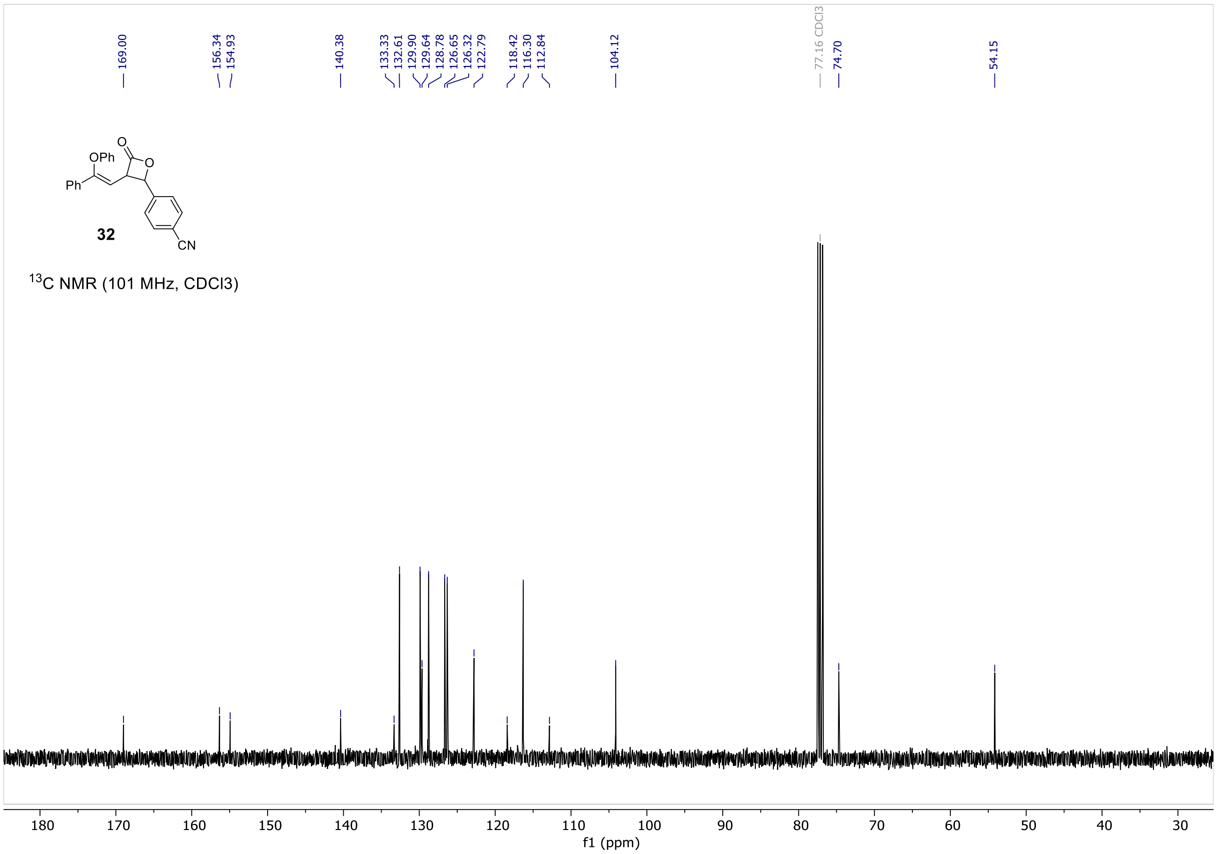
169.00
156.34
154.93
140.38
133.33
132.61
129.90
129.64
128.78
126.65
126.32
122.79
118.42
116.30
112.84
104.12
77.16 CDCl3
74.70
54.15
OPh
O
O
Ph
32
CN
13C NMR (101 MHz, CDCl3)
180
170
160
150
140
130
120
110
100
90
80
70
60
50
40
30
f1 (ppm)

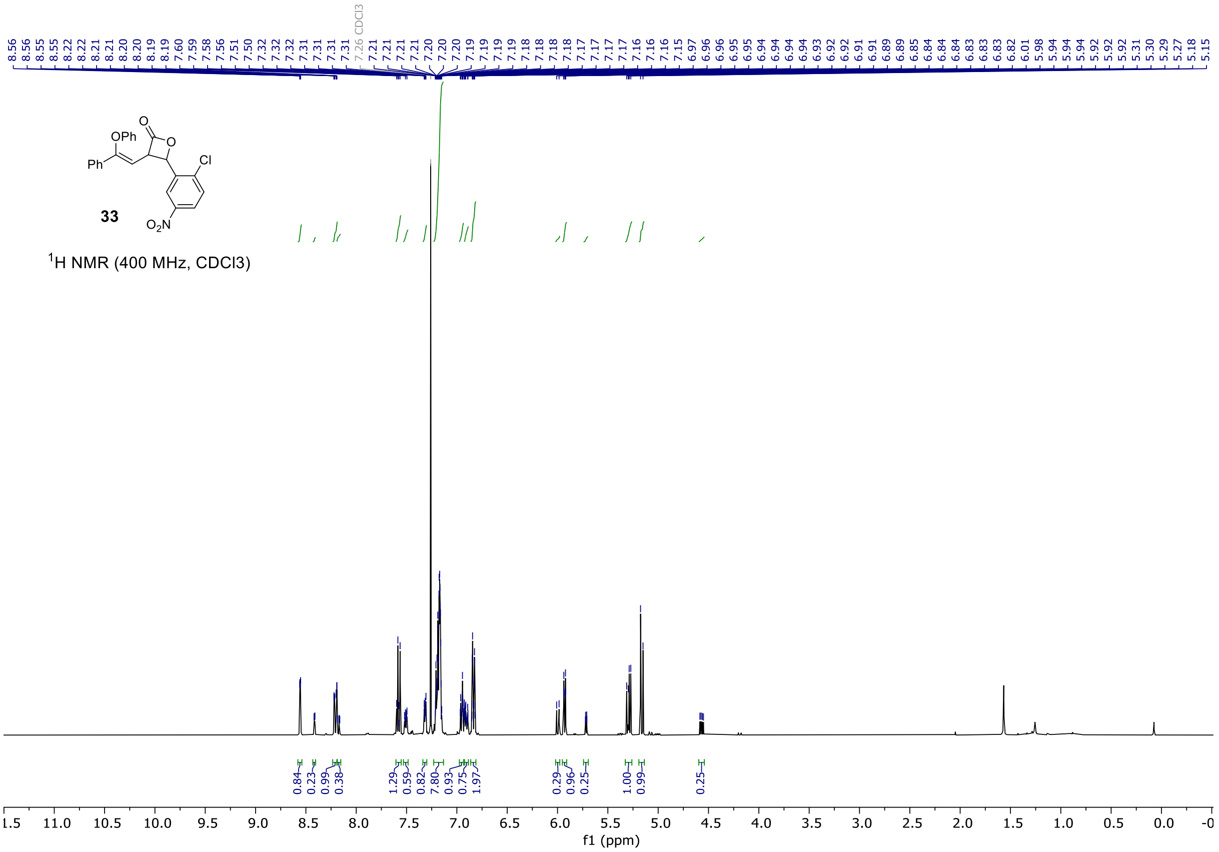
8.56 8.56 8.55 8.22 8.21 8.21 8.20 8.19 8.19 7.60 7.59 7.58 7.56 7.51 7.50 7.32 7.32 7.32 7.31 7.31 7.31 7.31 7.26 CDCl3 7.21 7.21 7.21 7.21 7.20 7.20 7.20 7.19 7.19 7.19 7.19 7.18 7.18 7.18 7.17 7.17 7.17 7.17 7.16 7.16 7.15 6.97 6.96 6.96 6.95 6.95 6.94 6.94 6.94 6.94 6.93 6.92 6.92 6.92 6.91 6.91 6.89 6.85 6.84 6.84 6.84 6.83 6.83 6.83 6.82 6.01 5.98 5.94 5.94 5.94 5.92 5.92 5.92 5.31 5.30 5.29 5.27 5.18 5.15
OPh
Ph
O
Cl
33
O_2N
^{1}H NMR (400 MHz, CDCl3)
0.84 0.23 0.99 0.38 1.29 0.59 0.82 7.80 0.93 0.75 1.97 0.29 0.96 0.25 1.00 0.99 0.25
f1 (ppm)

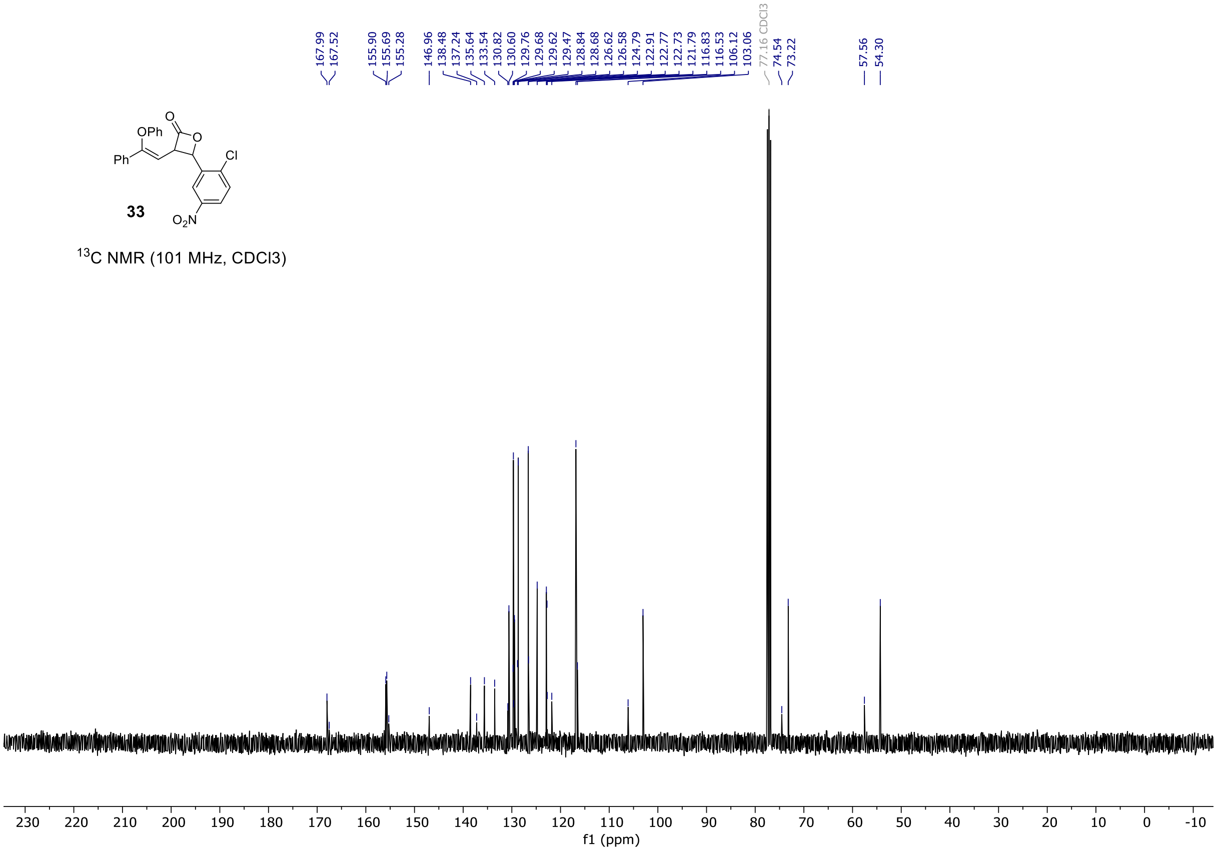
167.99 167.52 155.90 155.69 155.28 146.96 138.48 137.24 135.64 133.54 130.82 130.60 129.76 129.68 129.62 129.47 128.84 128.68 126.62 126.58 124.79 122.91 122.77 122.73 121.79 116.83 116.53 106.12 103.06 77.16 CDCl3 74.54 73.22 57.56 54.30
OPh
Ph
O
Cl
33
O_2N
^{13}C NMR (101 MHz, CDCl3)
f1 (ppm)

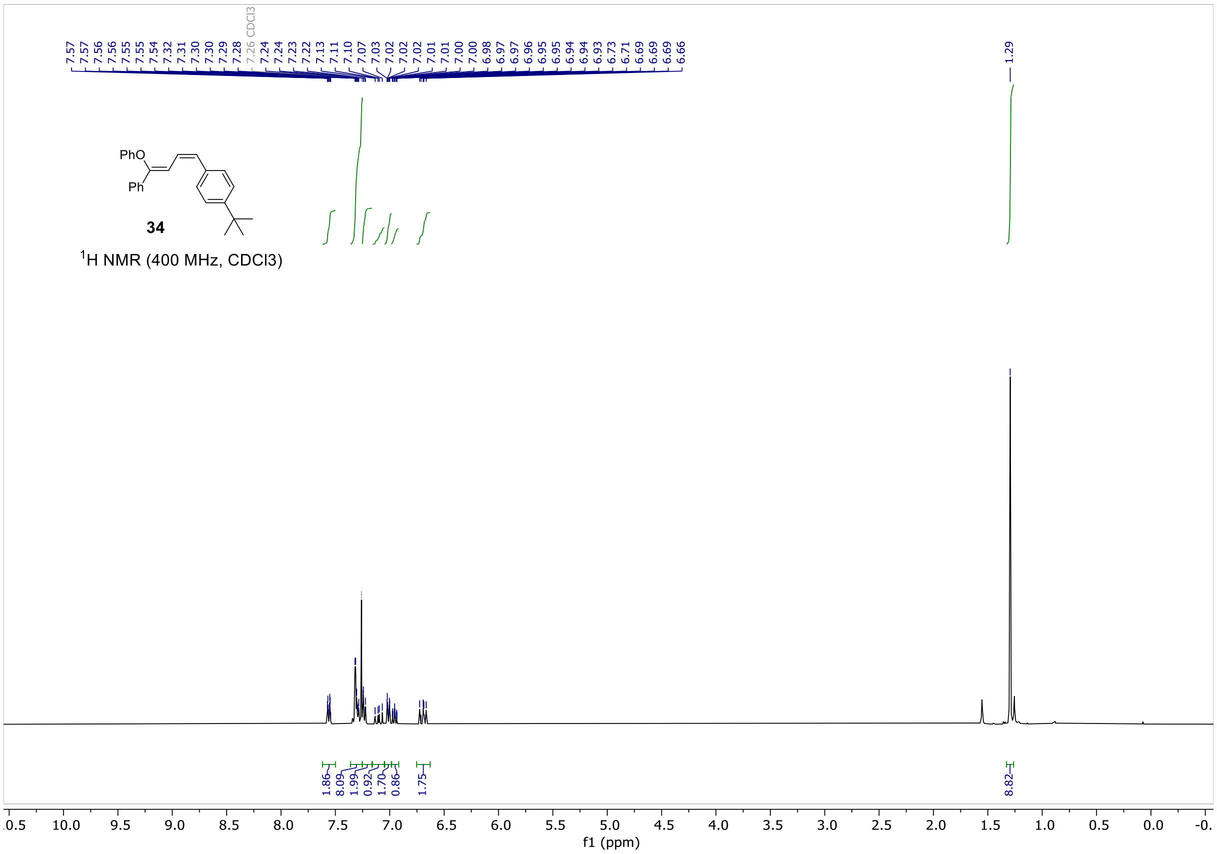

PhO
Ph
34
^{1}H NMR (400 MHz, CDCl3)
7.57 7.57 7.56 7.56 7.55 7.55 7.54 7.32 7.31 7.30 7.30 7.29 7.28 7.26 CDCl3 7.24 7.24 7.23 7.22 7.13 7.11 7.10 7.07 7.03 7.02 7.02 7.02 7.01 7.01 7.00 6.98 6.97 6.97 6.96 6.95 6.95 6.94 6.94 6.93 6.73 6.71 6.69 6.69 6.69 6.66
1.29
1.86 8.09 1.99 0.92 1.70 0.86 1.75
8.82
f1 (ppm)

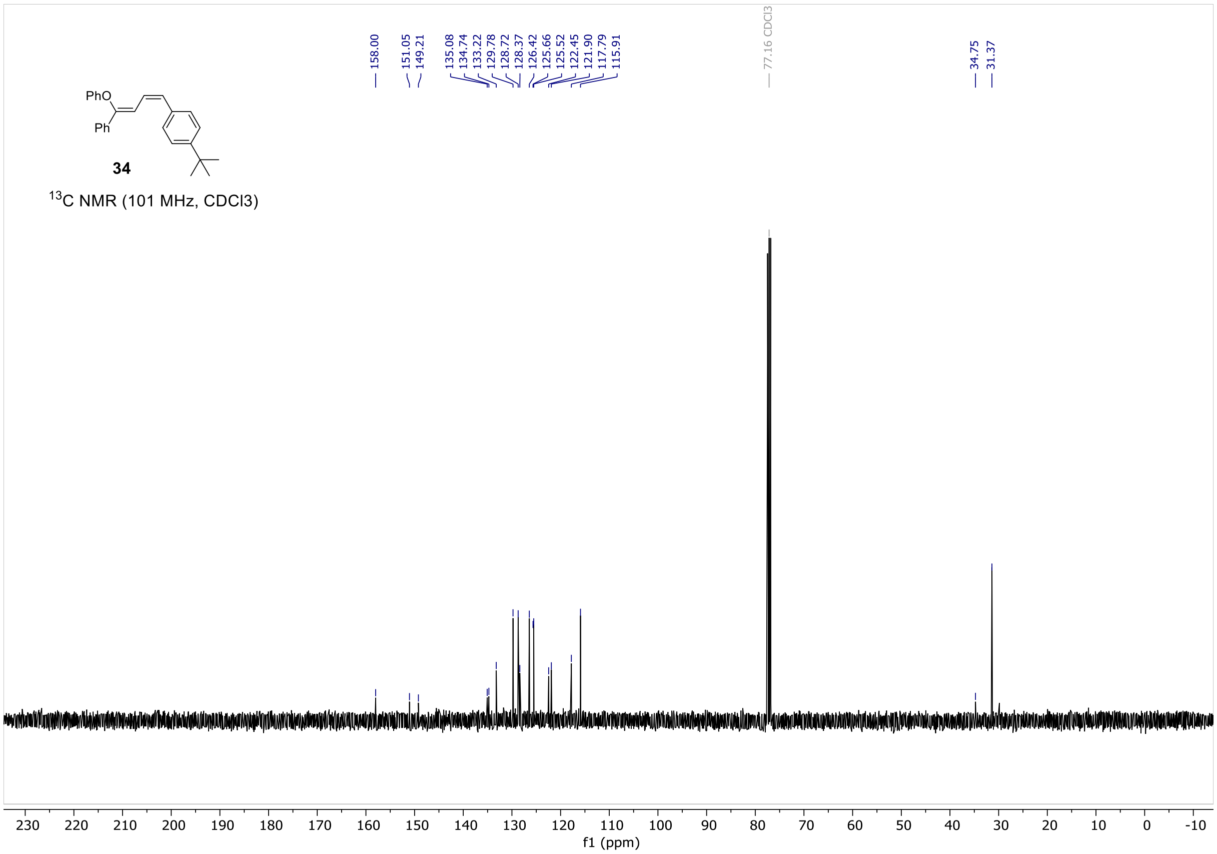

PhO
Ph
34
^{13}C NMR (101 MHz, CDCl3)
158.00 151.05 149.21 135.08 134.74 133.22 129.78 128.72 128.37 126.42 125.66 125.52 122.45 121.90 117.79 115.91
77.16 CDCl3
34.75 31.37
f1 (ppm)

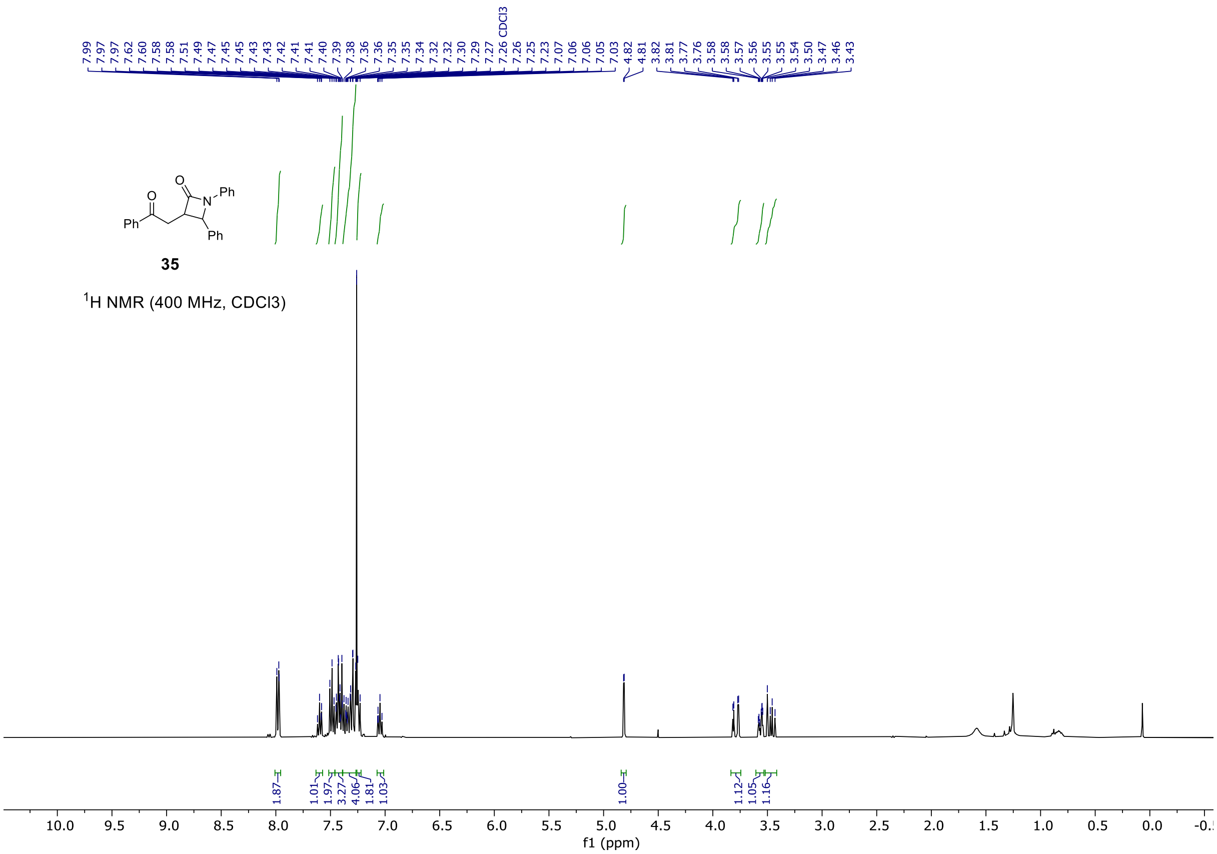
35
1H NMR (400 MHz, CDCl3)
f1 (ppm)

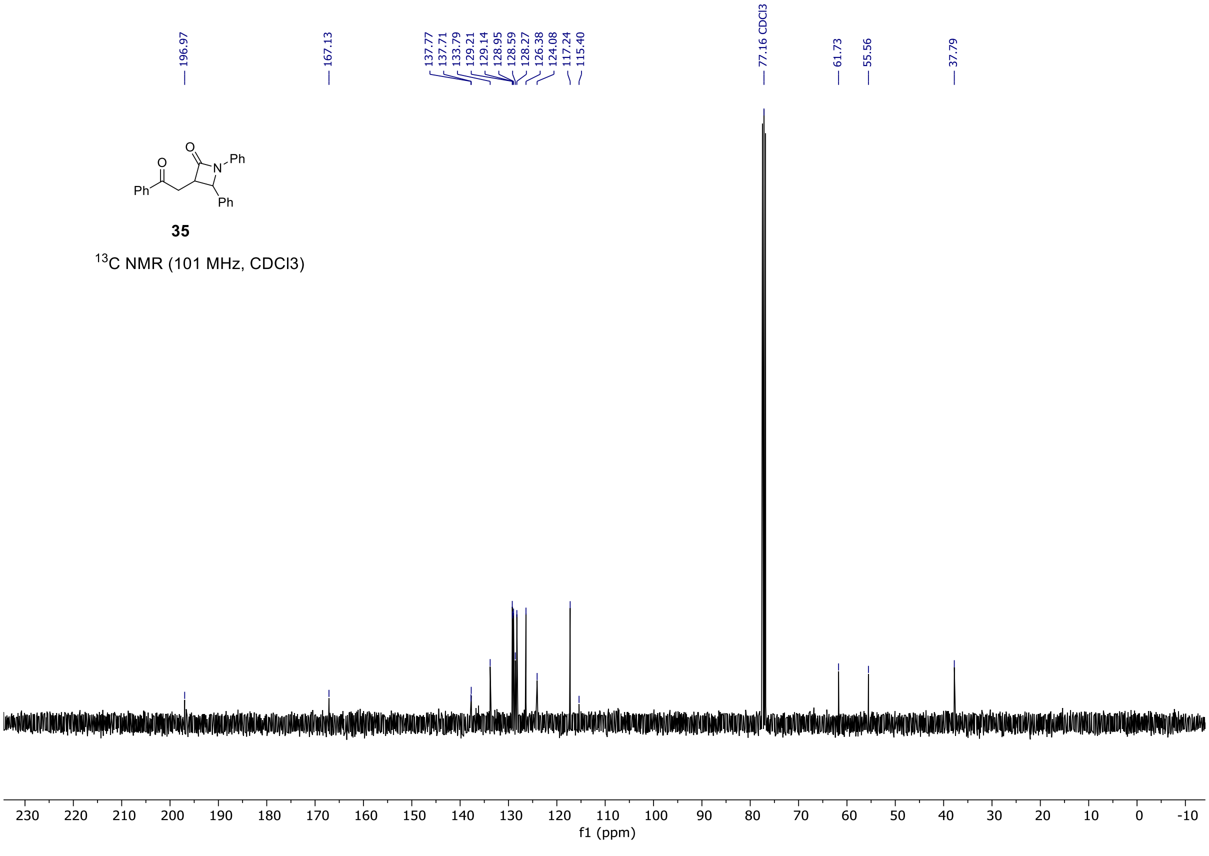
35
13C NMR (101 MHz, CDCl3)
196.97
167.13
137.77
137.71
133.79
129.21
129.14
128.95
128.59
128.27
126.38
124.08
117.24
115.40
77.16 CDCl3
61.73
55.56
37.79
f1 (ppm)

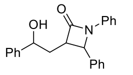

36

^{1}H NMR (400 MHz, CDCl3)

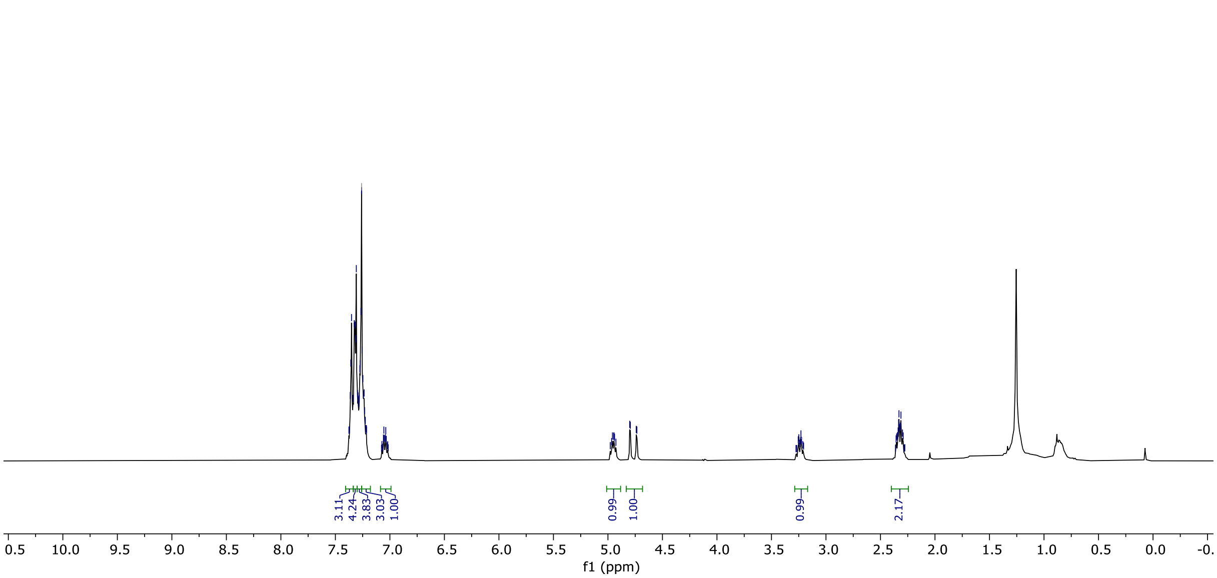

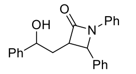

36

^{13}C NMR (101 MHz, CDCl3)

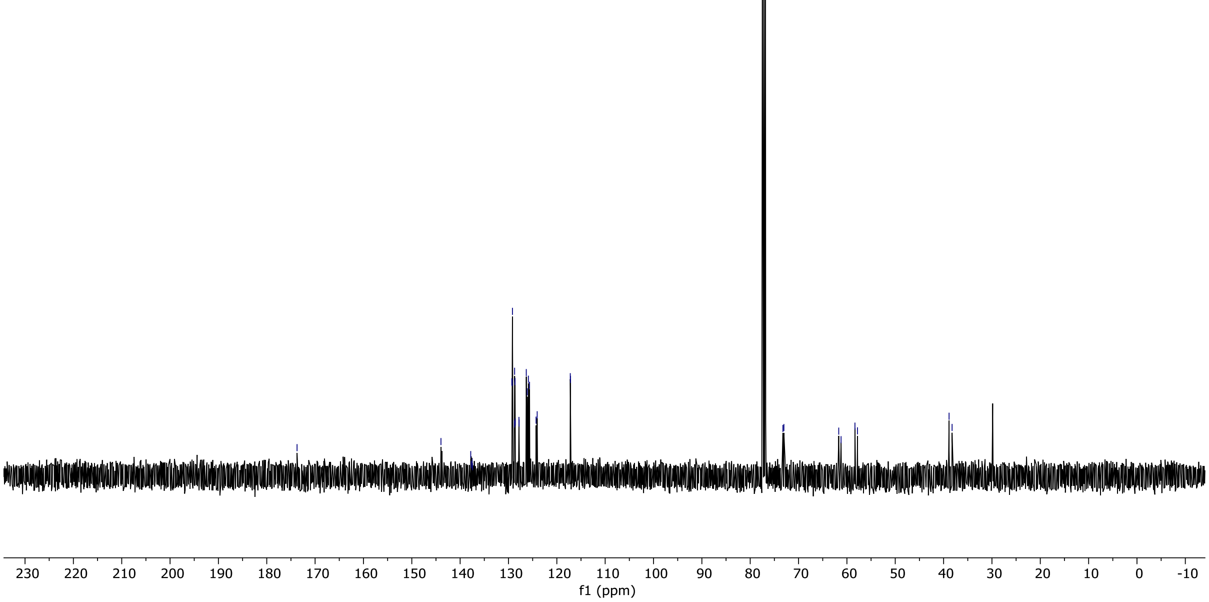

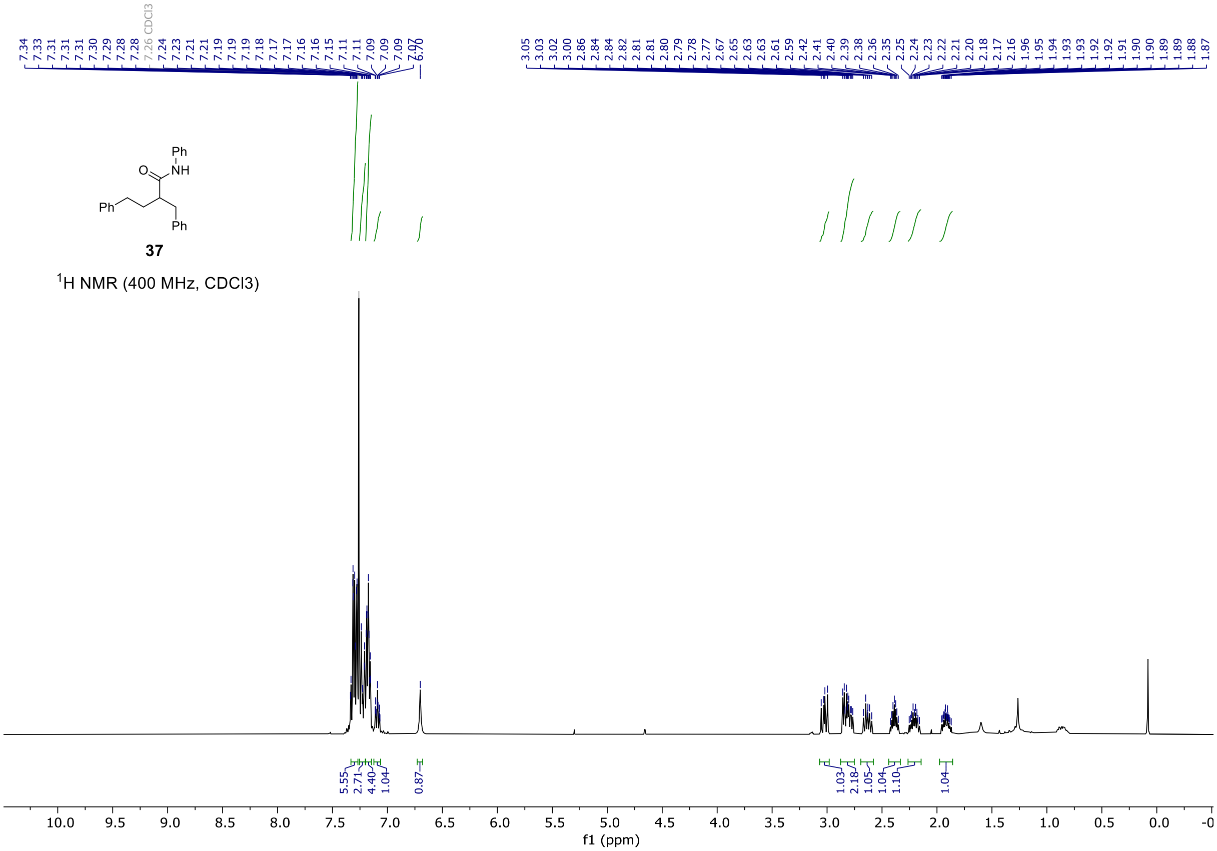
7.34 7.33 7.31 7.31 7.30 7.29 7.28 7.28 7.26 CDCl3 7.24 7.23 7.21 7.21 7.19 7.19 7.18 7.17 7.17 7.16 7.16 7.15 7.11 7.11 7.09 7.09 7.07 6.70
3.05 3.03 3.02 3.00 2.86 2.84 2.84 2.82 2.81 2.81 2.80 2.79 2.78 2.77 2.67 2.65 2.63 2.63 2.61 2.59 2.42 2.41 2.40 2.39 2.38 2.36 2.35 2.25 2.24 2.23 2.22 2.21 2.20 2.18 2.17 2.16 1.96 1.95 1.94 1.93 1.93 1.92 1.92 1.91 1.90 1.89 1.88 1.87
Ph
O
NH
Ph
Ph
37
1H NMR (400 MHz, CDCl3)
5.55 2.71 4.40 1.04 0.87
1.03 2.18 1.05 1.04 1.10 1.04
10.0 9.5 9.0 8.5 8.0 7.5 7.0 6.5 6.0 5.5 5.0 4.5 4.0 3.5 3.0 2.5 2.0 1.5 1.0 0.5 0.0
f1 (ppm)

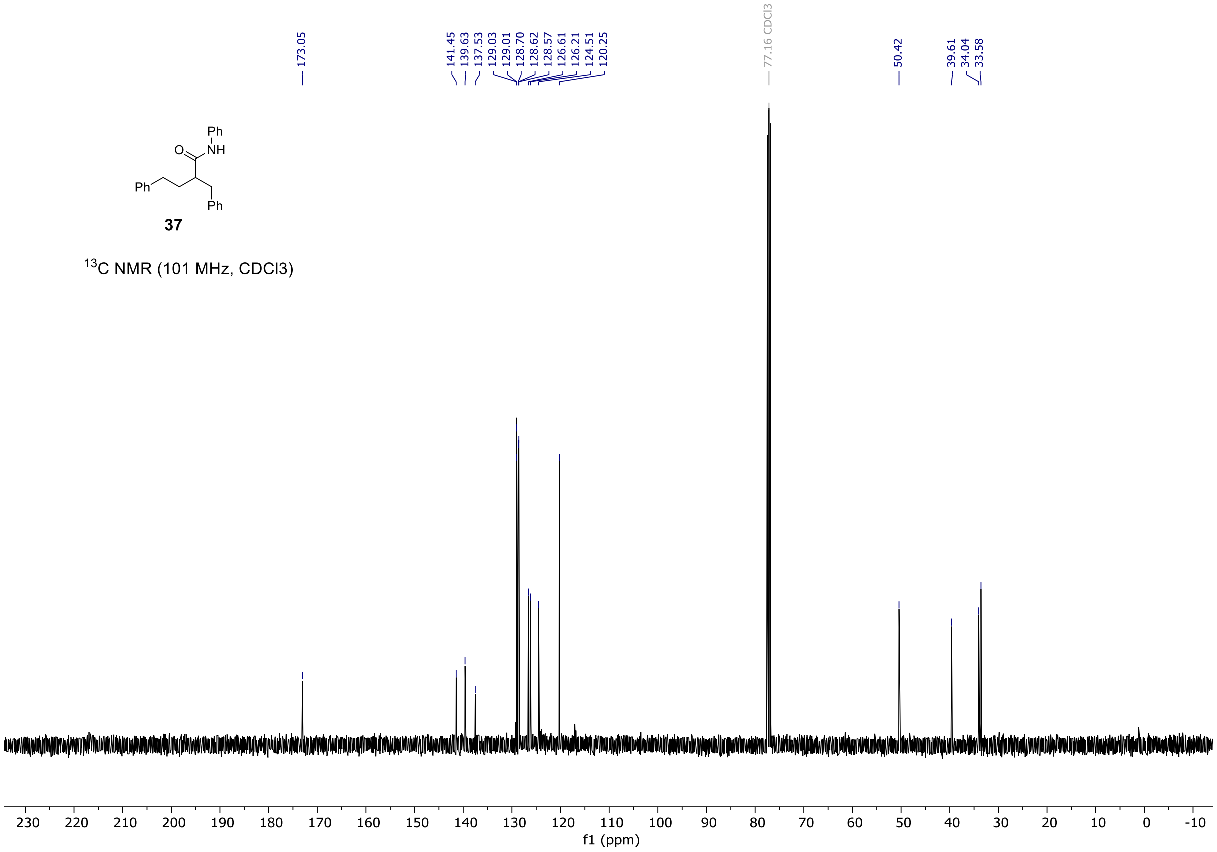
173.05
141.45 139.63 137.53 129.03 129.01 128.70 128.62 128.57 126.61 126.21 124.51 120.25
77.16 CDCl3
50.42
39.61 34.04 33.58
Ph
O
NH
Ph
Ph
37
13C NMR (101 MHz, CDCl3)
230 220 210 200 190 180 170 160 150 140 130 120 110 100 90 80 70 60 50 40 30 20 10 0 -10
f1 (ppm)

References

[1] G.M. Sheldrick. Crystal structure refinement with SHELXL. Acta Crystallogr. C Struct. Chem., 71 (2015), pp. 3-8, 10.1107/S2053229614024218

[2] O.V. Dolomanov, L.J. Bourhis, R.J. Gildea, J.A.K. Howard, H. Puschmann. OLEX2: a complete structure solution, refinement and analysis program. J. Appl. Crystallogr., 42 (2009), pp. 339-341, 10.1107/S0021889808042726

[3] Zimmerman, H. E.; Durr, H. G.; Givens, R. S.; Lewis, R. G. The Photochemistry of Dibenzoylethylenes. Mechanistic and Exploratory Organic Photochemistry. XXII. J. Am. Chem. Soc. 1967, 89 (8), 1863–1874, 10.1021/ja00984a019
